# Supplementary material for: Hominoid-specific transposable elements reshaped neural crest migration in craniofacial development
Source: Mol Syst Biol. 2025 Sep 22;21(12):1731–47. doi: 10.1038/s44320-025-00151-z (PMC12673149; doi:10.1038/s44320-025-00151-z)
Supplement: Supplementary file 1 — Appendix [file 44320_2025_151_MOESM1_ESM.pdf]

Appendix – Table of contents

Table of contents	1
Appendix Figure S1	2
Appendix Figure S2	3
Appendix Figure S3	4
Appendix Figure S4	5
Appendix Figure S5	6
Appendix Figure S6	7
Appendix Figure S7	8

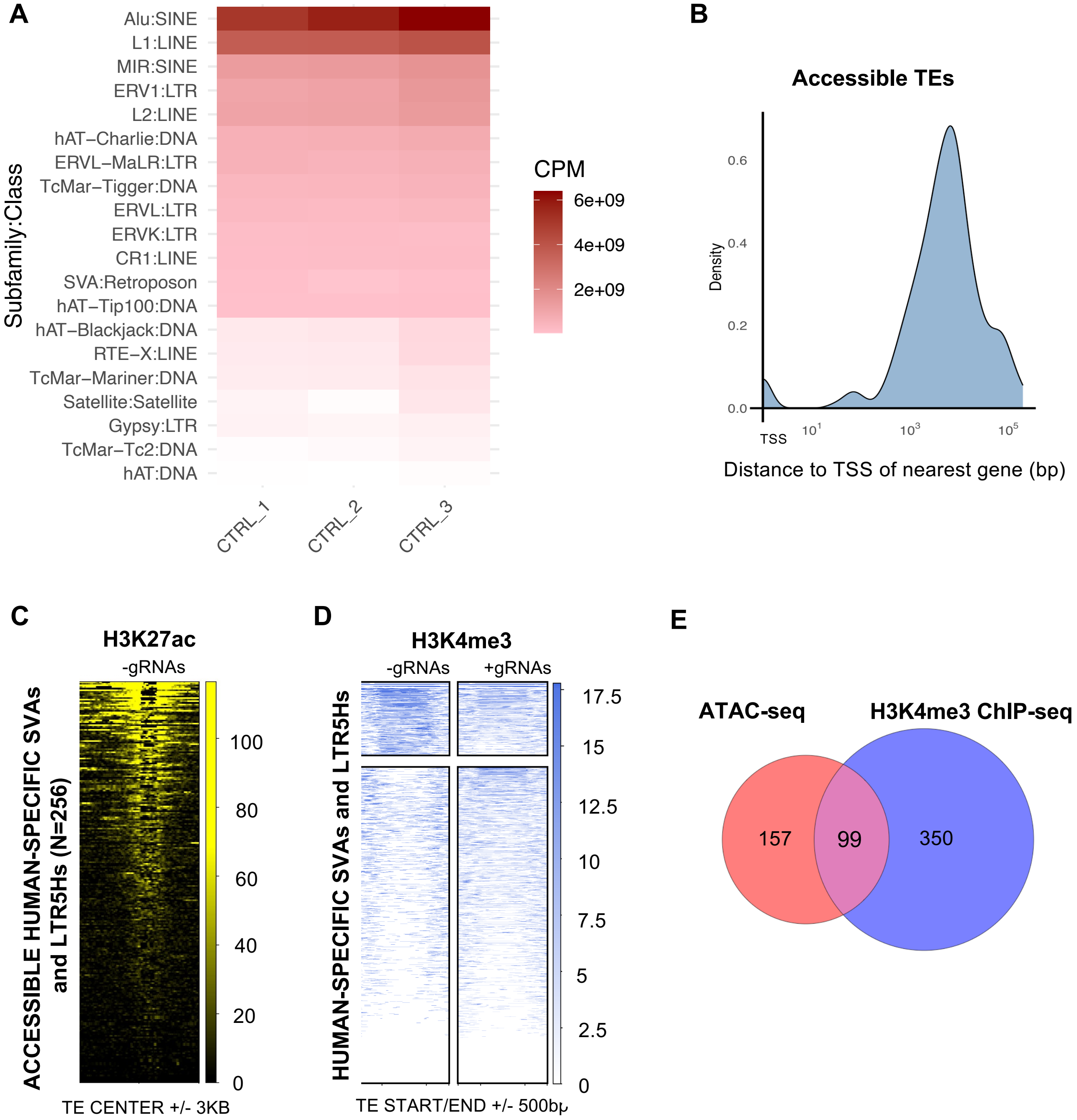

Appendix Figure S1 – (a) Top 20 transposable element (TE) subfamilies ranked by counts per million (CPM) expression across three CNCC replicates. **(b)** Density plot of accessible human-specific SVAs and LTR5Hs in relation to the transcription start site of their nearest genes. **(c)** ChIP-seq heatmap displaying H3K27ac signal at SVAs and LTR5Hs accessible in CNCCs. Bigwig used for heatmap was normalized by sequencing depth. **(d)** ChIP-seq heatmap displaying H3K4me3 signal at human-specific SVAs and LTR5Hs. Bigwig used for heatmap was normalized by sequencing depth. **(e)** Venn diagram illustrating the overlap between human-specific SVAs and LTR5Hs that are both accessible and marked by H3K4me3. Bigwigs used for heatmap are normalised by sequencing depth.

A

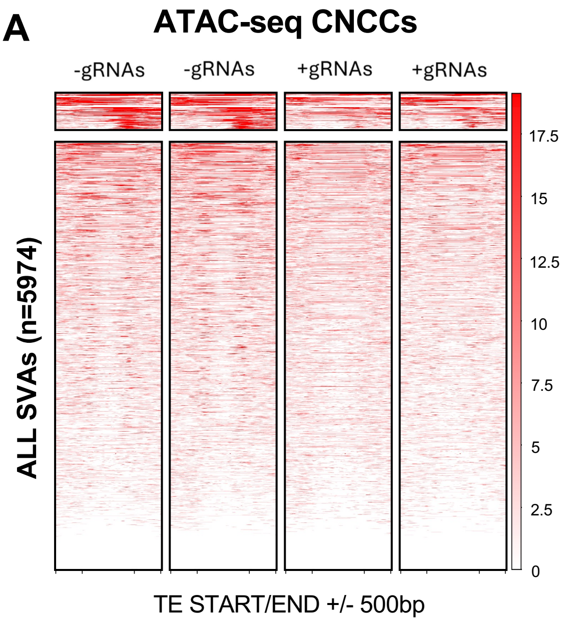

B

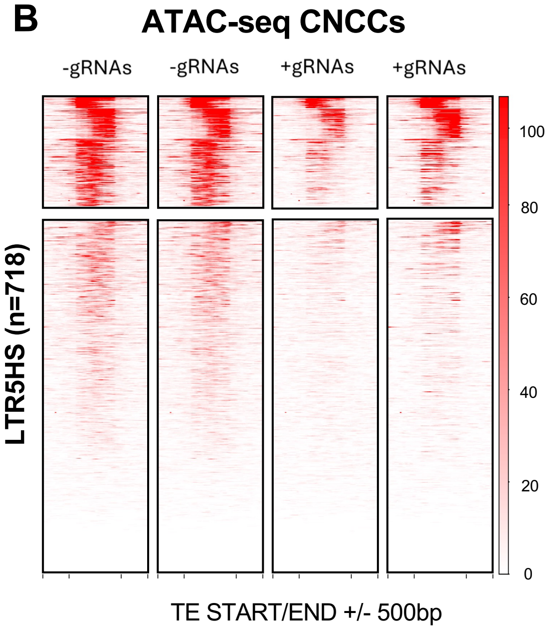

C

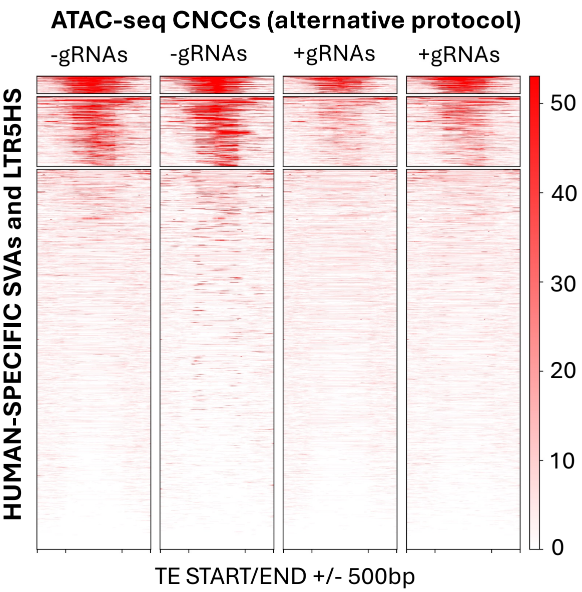

D

Motifs enriched in accessible SVAs

TWIST1	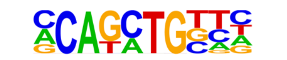	46%
HOMEOBOX	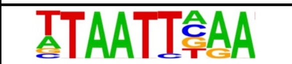	36%
SOX9	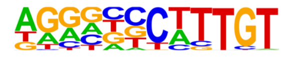	43%
SOX10	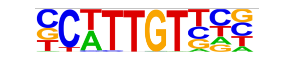	50%

E

Motifs enriched in accessible LTR5HS

TWIST1	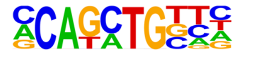	89%
HOMEOBOX	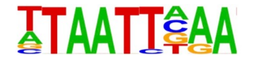	66%
TFAP2A	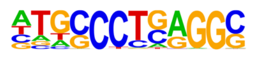	75%
SLUG	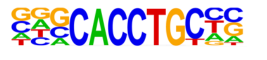	67%
SNAIL1	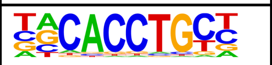	76%
ZEB1	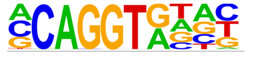	93%
ZEB2	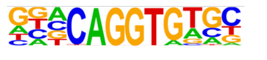	75%
SOX9	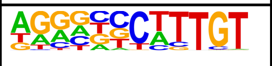	66%
SOX10	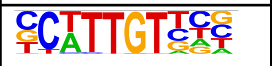	95%
FOXD3	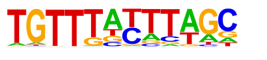	37%

Appendix Figure S2 – (a,b) ATAC-seq heatmap showing chromatin accessibility at all SVAs **(a)** and LTR5Hs **(b)** in CNCCs. **(c)** ATAC-seq heatmap showing chromatin accessibility at human-specific SVAs and LTR5Hs in CNCCs generated with alternative protocol (Bajpai et al. 2010). Bigwigs used for heatmaps were normalized by sequencing depth. **(d,e)** Motif analysis for all accessilbe SVAs **(d)** and LTR5Hs **(e)**.

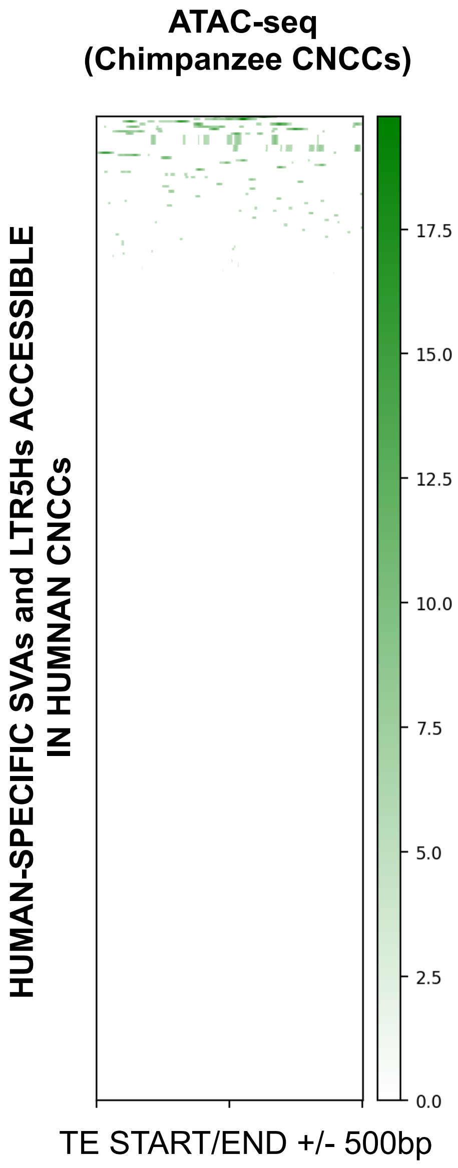

Appendix Figure S3. Chimpanzee CNCC ATAC-seq heatmap centered on regions up and downstream (+/- 250 bp) to the 256 accessible LTR5Hs and human-specific SVAs. The bigwig used for the heatmap is normalized by sequencing depth. The chimpanzee ATAC-seq data were publicly available (Prescott et al. 2015).

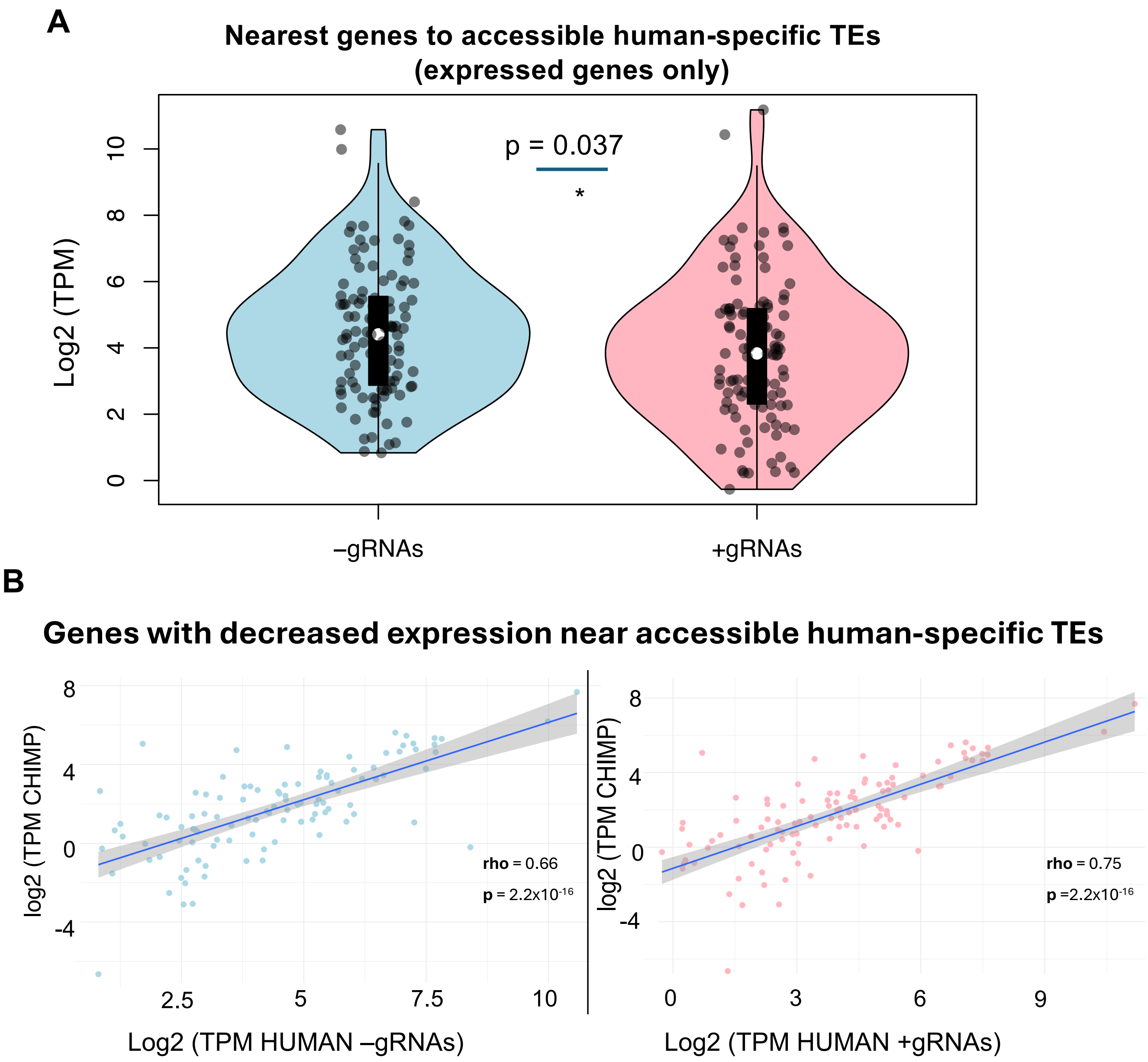

Appendix Figure S4 – (a) Violin plots showing the expression of the genes representing the nearest gene to an accessible human-specific LTR5Hs or SVA (expressed genes only, N = 107; p-value calculated with Wilcoxon's Rank Sum test). **(b)** Correlation plots showing correlation of expression for the same set of genes between human (+ and – gRNAs) and chimpanzee CNCCs. Correlation was statistically evaluated with the Spearman's rank correlation coefficient.

WEB-based GEne SeT AnaLysis Toolkit

WebGestalt

Translating gene lists into biological insights...

Summary

Result Download

Job summary

GO Slim summary for the user uploaded IDs

Enrichment Results

Redundancy reduction: ● None ○ Weighted set cover

Table | Bar chart | Volcano plot | DAG

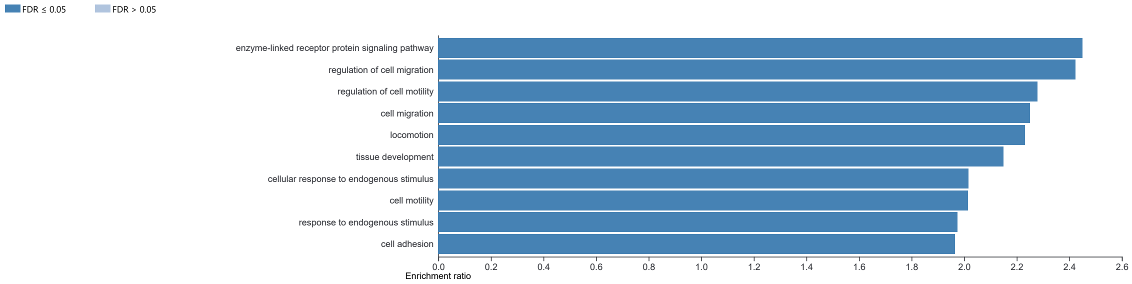

Select an enriched analyte set...

GO:0009888: tissue development

Analyte set: GO:0009888 tissue development

FDR 1.9573e-11

P Value 2.1658e-15

Analyte Set Size 1981

Expected Value 51.182

Overlap 110

Enrichment Ratio 2.1492

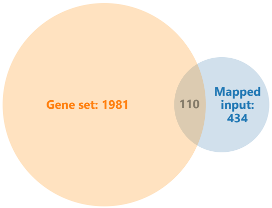

User ID ↑	Gene Symbol	Gene Name	Entrez Gene ID
ADAMTSL4	ADAMTSL4	ADAMTS like 4	54507
ALPK2	ALPK2	alpha kinase 2	115701
AMBN	AMBN	ameloblastin	258
AMER2	AMER2	APC membrane recruitment protein 2	219287
APELA	APELA	apelin receptor early endogenous ligand	100506013
ATP2B1	ATP2B1	ATPase plasma membrane Ca2+ transporting 1	490
ATP2C2	ATP2C2	ATPase secretory pathway Ca2+ transporting 2	9914
BMPER	BMPER	BMP binding endothelial regulator	168667
BNC1	BNC1	basonuclin zinc finger protein 1	646
BNC2	BNC2	basonuclin zinc finger protein 2	54796

10 per page

1 2 ... 11

WebGestalt is currently developed and maintained by Yuxing Liao, Suhas Vasaikar, Zhiao Shi and Bing Zhang at the Zhang Lab. Other people who have made significant contribution to the project include Jing Wang, Dexter Duncan, Stefan Kirov and Jay Snoddy.
Funding credits: NIH/NCI (U24 CA210954); Leidos (15X038); CPRIT (RR160027); NIH/NIAAA (U01 AA016662, U01 AA013512); NIH/NIDA (P01 DA015027); NIH/NIMH (P50 MH078028, P50 MH096972); NIH/NCI (U24 CA159988); NIH/NIGMS (R01 GM088822).

Appendix Figure S5 – Webgestalt output for Gene Ontology enrichment terms associated with the significantly downregulated genes in CNCCs RNAseq data at day 5.

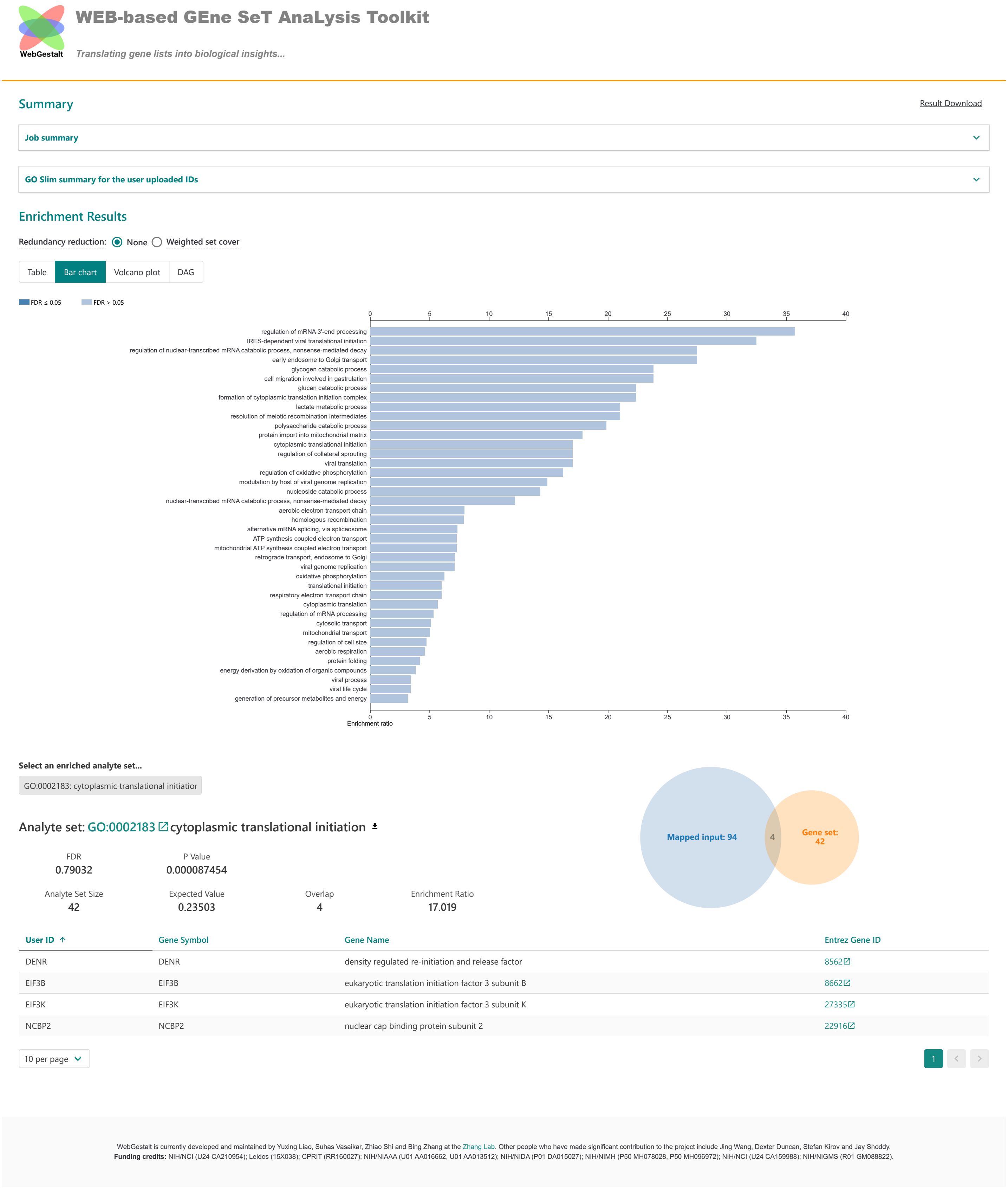

Appendix Figure S6 – Webgestalt output for Gene Ontology enrichment terms on the expressed genes located nearest to accessible LTR5Hs and human-specific SVAs in CNCCs at day 5.

WebGestalt

WEB-based GEne SeT AnaLysis Toolkit

Translating gene lists into biological insights...

Summary

Result Download

Job summary

GO Slim summary for the user uploaded IDs

Enrichment Results

Redundancy reduction: ◉ None ○ Weighted set cover

Table | Bar chart | Volcano plot | DAG

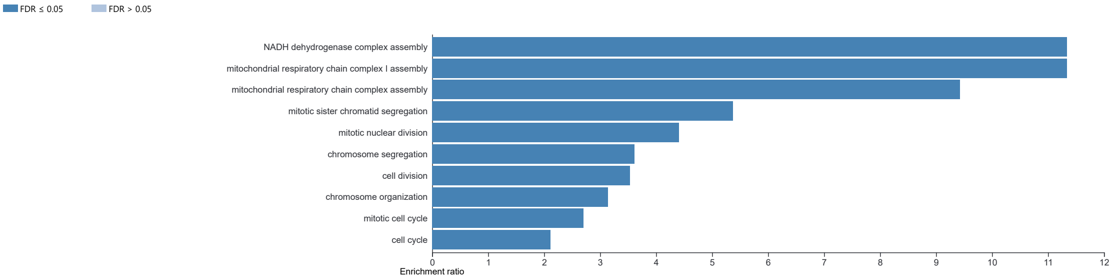

Select an enriched analyte set...

GO:0033108: mitochondrial respiratory chain cc

Analyte set: GO:0033108 mitochondrial respiratory chain complex assembly

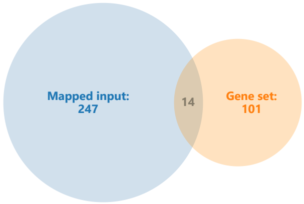

FDR	P Value		
0.0000012680	2.5175e-10		

Analyte Set Size	Expected Value	Overlap	Enrichment Ratio
101	1.4851	14	9.4269

User ID ↑	Gene Symbol	Gene Name	Entrez Gene ID
COA1	COA1	cytochrome c oxidase assembly factor 1	55744
COX17	COX17	cytochrome c oxidase copper chaperone COX17	10063
DMAC1	DMAC1	distal membrane arm assembly component 1	90871
NDUFA5	NDUFA5	NADH:ubiquinone oxidoreductase subunit A5	4698
NDUFAF2	NDUFAF2	NADH:ubiquinone oxidoreductase complex assembly factor 2	91942
NDUFAF4	NDUFAF4	NADH:ubiquinone oxidoreductase complex assembly factor 4	29078
NDUFAF8	NDUFAF8	NADH:ubiquinone oxidoreductase complex assembly factor 8	284184
NDUFB2	NDUFB2	NADH:ubiquinone oxidoreductase subunit B2	4708
NDUFB3	NDUFB3	NADH:ubiquinone oxidoreductase subunit B3	4709
NDUFB6	NDUFB6	NADH:ubiquinone oxidoreductase subunit B6	4712

10 per page

1 2 ‹ ›

WebGestalt is currently developed and maintained by Yuxing Liao, Suhas Vasaikar, Zhiao Shi and Bing Zhang at the Zhang Lab. Other people who have made significant contribution to the project include Jing Wang, Dexter Duncan, Stefan Kirov and Jay Snoddy.
Funding credits: NIH/NCI (U24 CA210954); Leidos (15X038); CPRIT (RR160027); NIH/NIAAA (U01 AA016662, U01 AA013512); NIH/NIDA (P01 DA015027); NIH/NIMH (P50 MH078028, P50 MH096972); NIH/NCI (U24 CA159988); NIH/NIGMS (R01 GM088822).

Appendix Figure S7 – Webgestalt output for Gene Ontology enrichment terms associated with the significantly upregulated genes in CNCCs RNAseq data at day 5.
