# Supplementary figures and images for: Hominoid-specific transposable elements reshaped neural crest migration in craniofacial development
Source: Mol Syst Biol. 2025 Sep 22;21(12):1731–47. doi: 10.1038/s44320-025-00151-z (PMC12673149; doi:10.1038/s44320-025-00151-z)

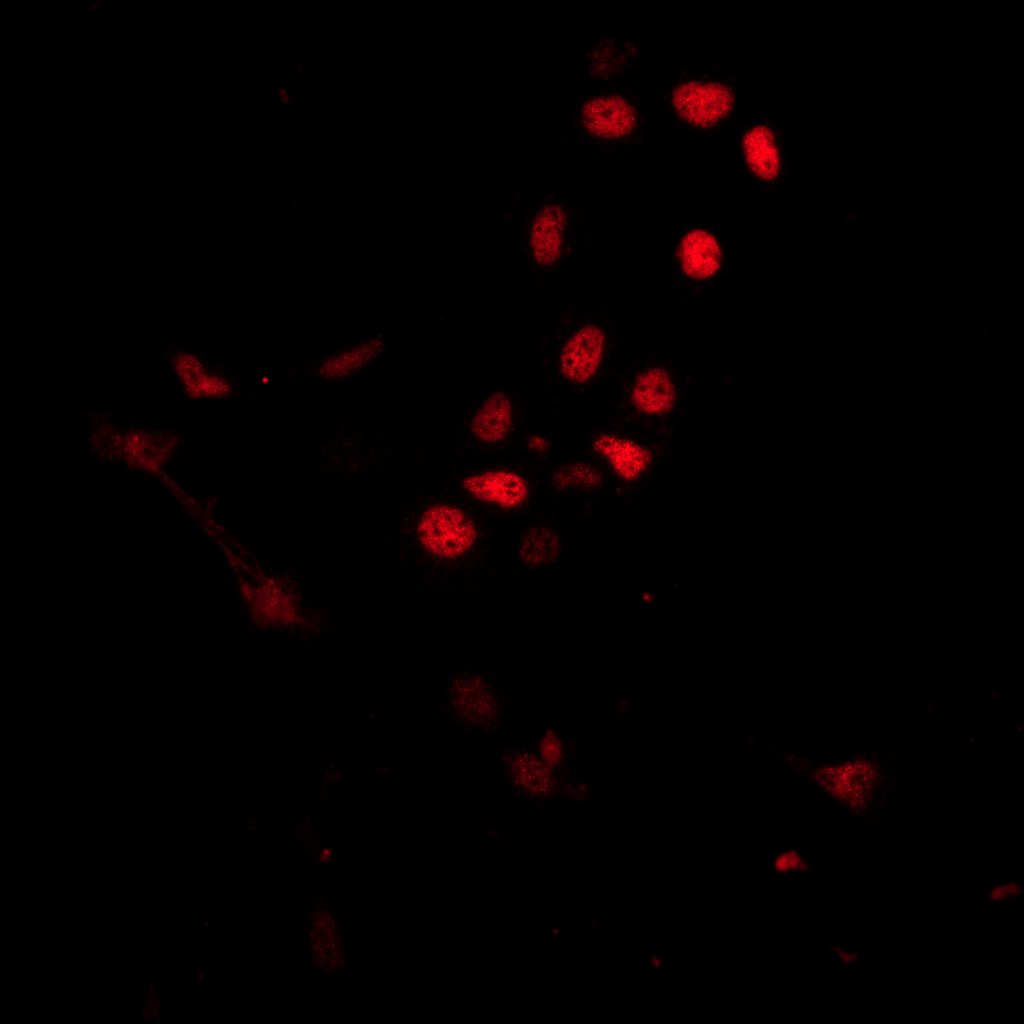

Supplement: Supplementary file 8 — Source data Fig. 1 [file 44320_2025_151_MOESM8_ESM.zip › FIGURE1/1E/242221-NCC-SOX9-TFAP2A/241121-NCC-paper-HSTE3-TFAP2A-40X-2-tfap2a.jpg]

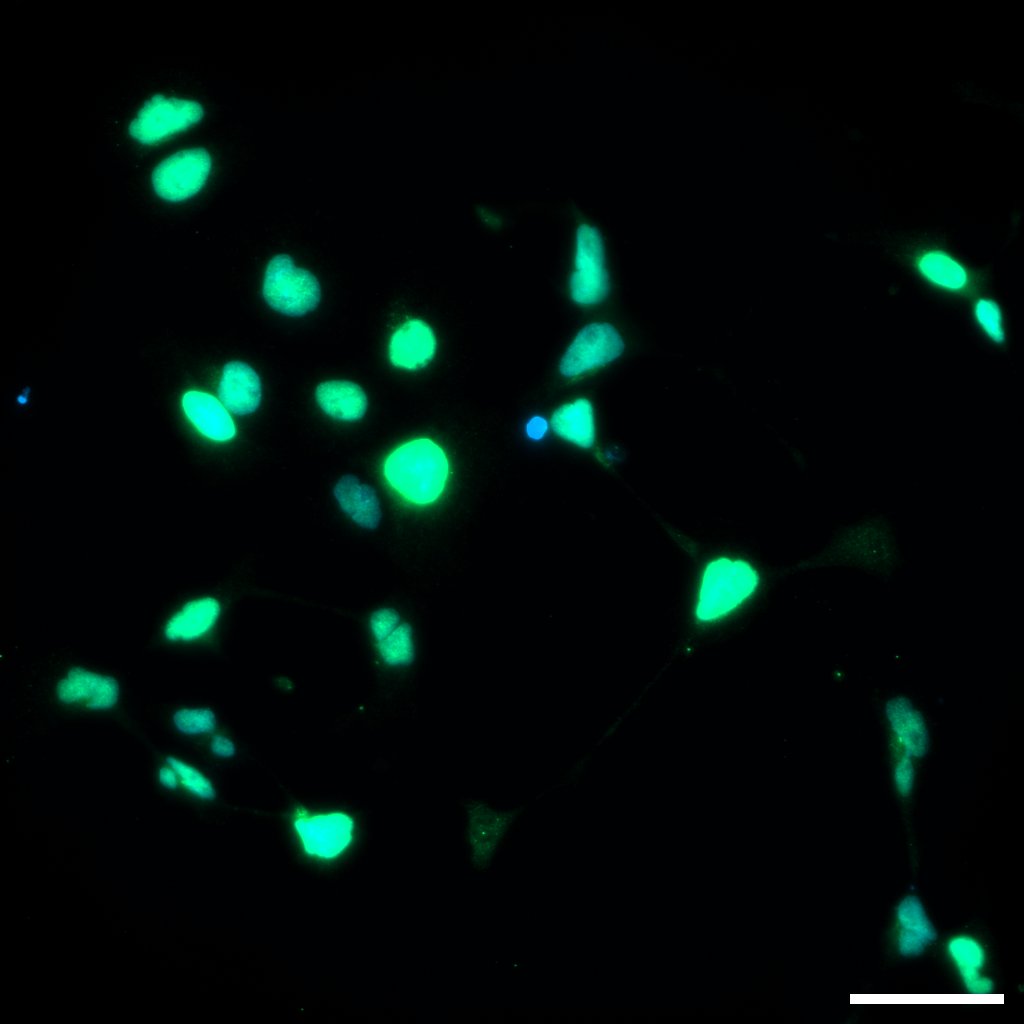

Supplement: Supplementary file 8 — Source data Fig. 1 [file 44320_2025_151_MOESM8_ESM.zip › FIGURE1/1E/242221-NCC-SOX9-TFAP2A/241121-NCC-paper-HSTE3-SOX9-40X-4-paper-scalebar.jpg]

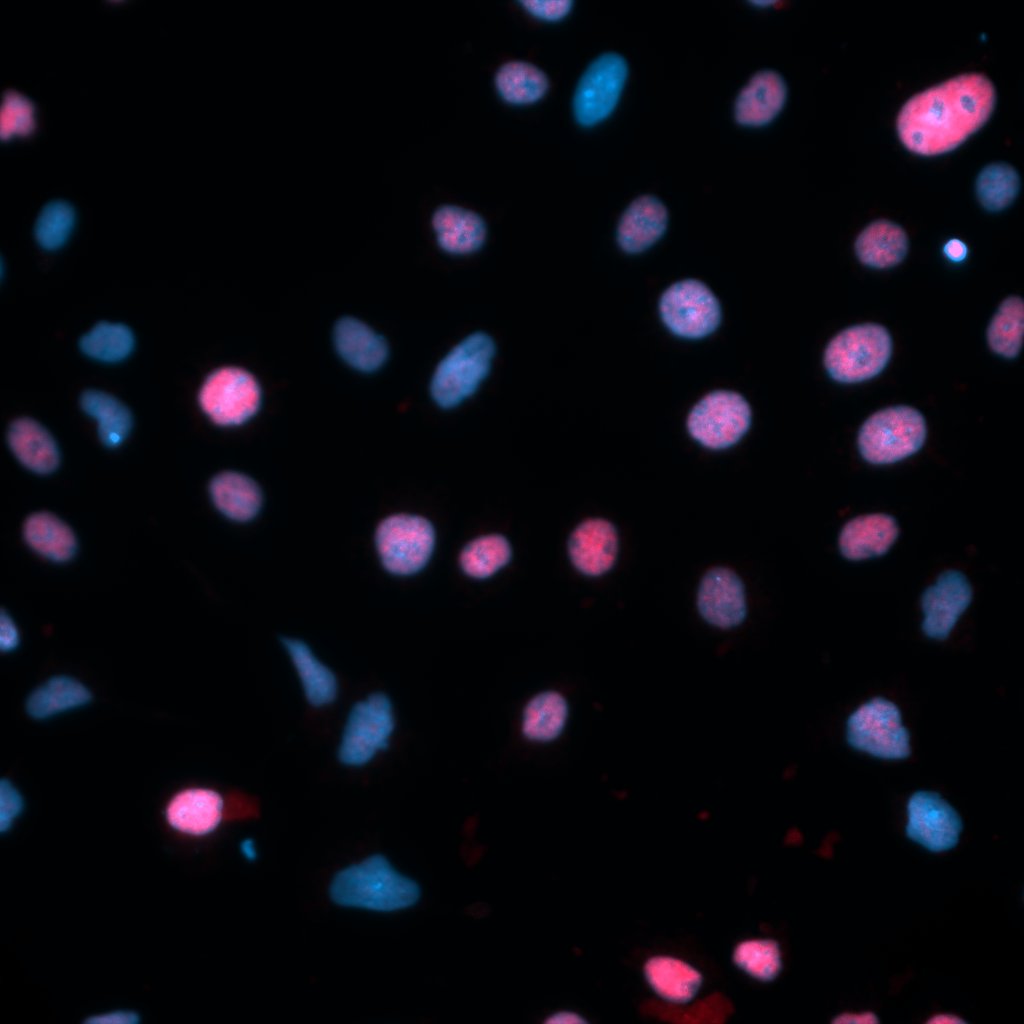

Supplement: Supplementary file 8 — Source data Fig. 1 [file 44320_2025_151_MOESM8_ESM.zip › FIGURE1/1E/242221-NCC-SOX9-TFAP2A/241121-NCC-paper-NOG3-TFAP2A-40X-composite.jpg]

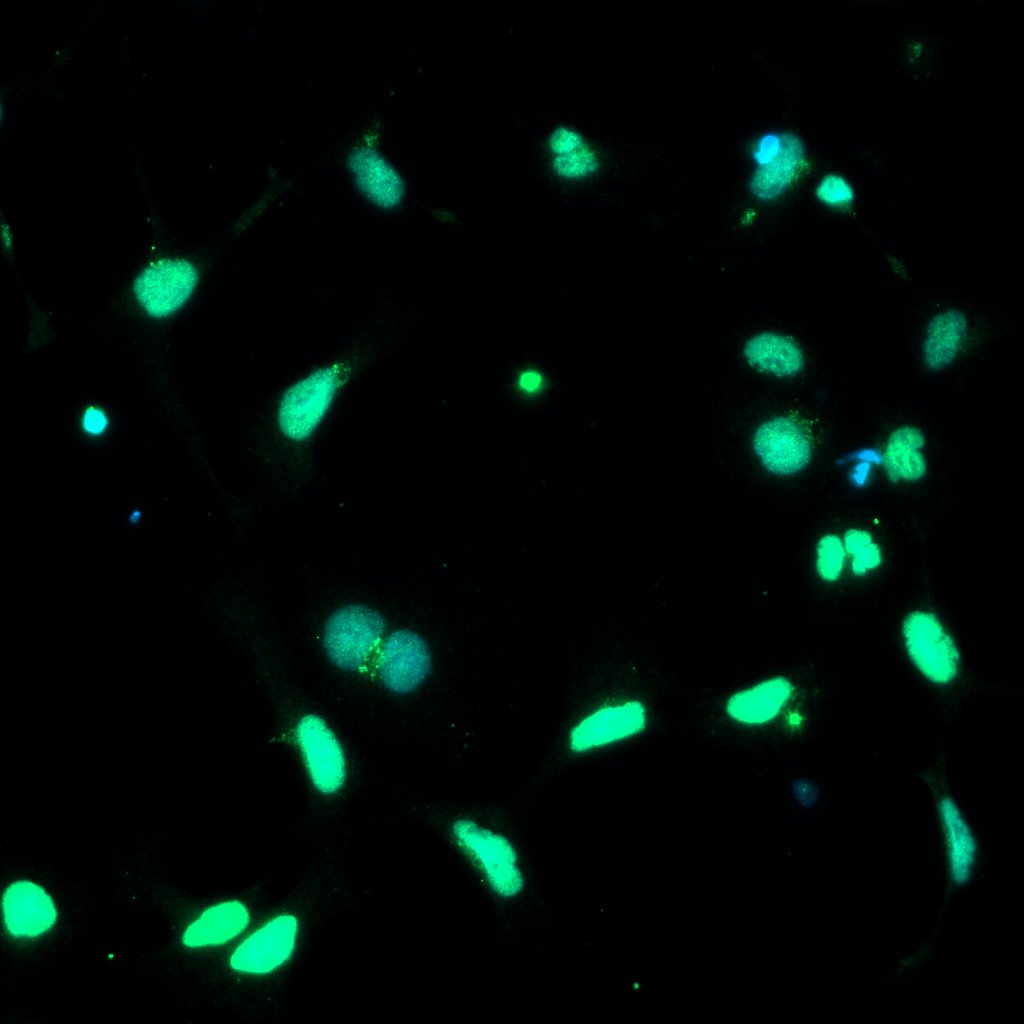

Supplement: Supplementary file 8 — Source data Fig. 1 [file 44320_2025_151_MOESM8_ESM.zip › FIGURE1/1E/242221-NCC-SOX9-TFAP2A/241121-NCC-paper-NOG3-SOX9-40X-5-paper.jpg]

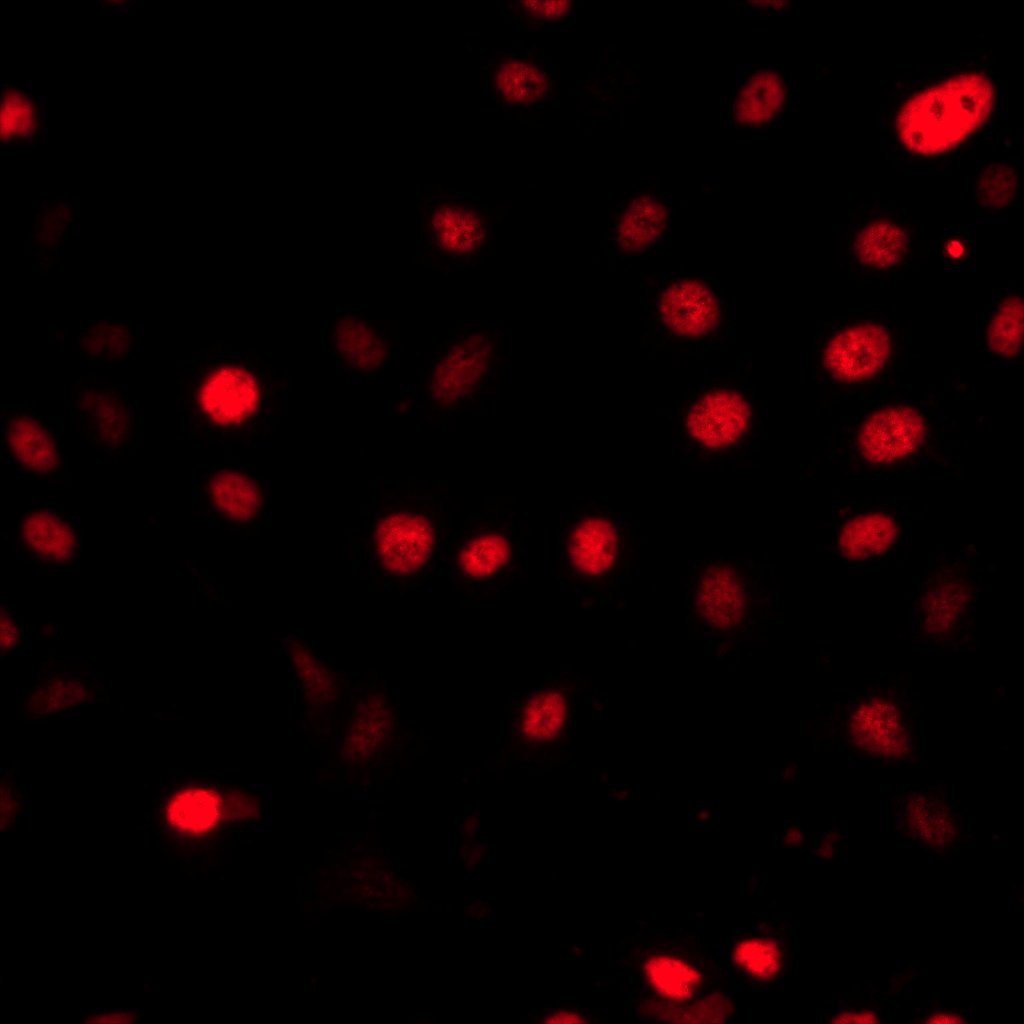

Supplement: Supplementary file 8 — Source data Fig. 1 [file 44320_2025_151_MOESM8_ESM.zip › FIGURE1/1E/242221-NCC-SOX9-TFAP2A/241121-NCC-paper-NOG3-TFAP2A-40X-tfap2a.jpg]

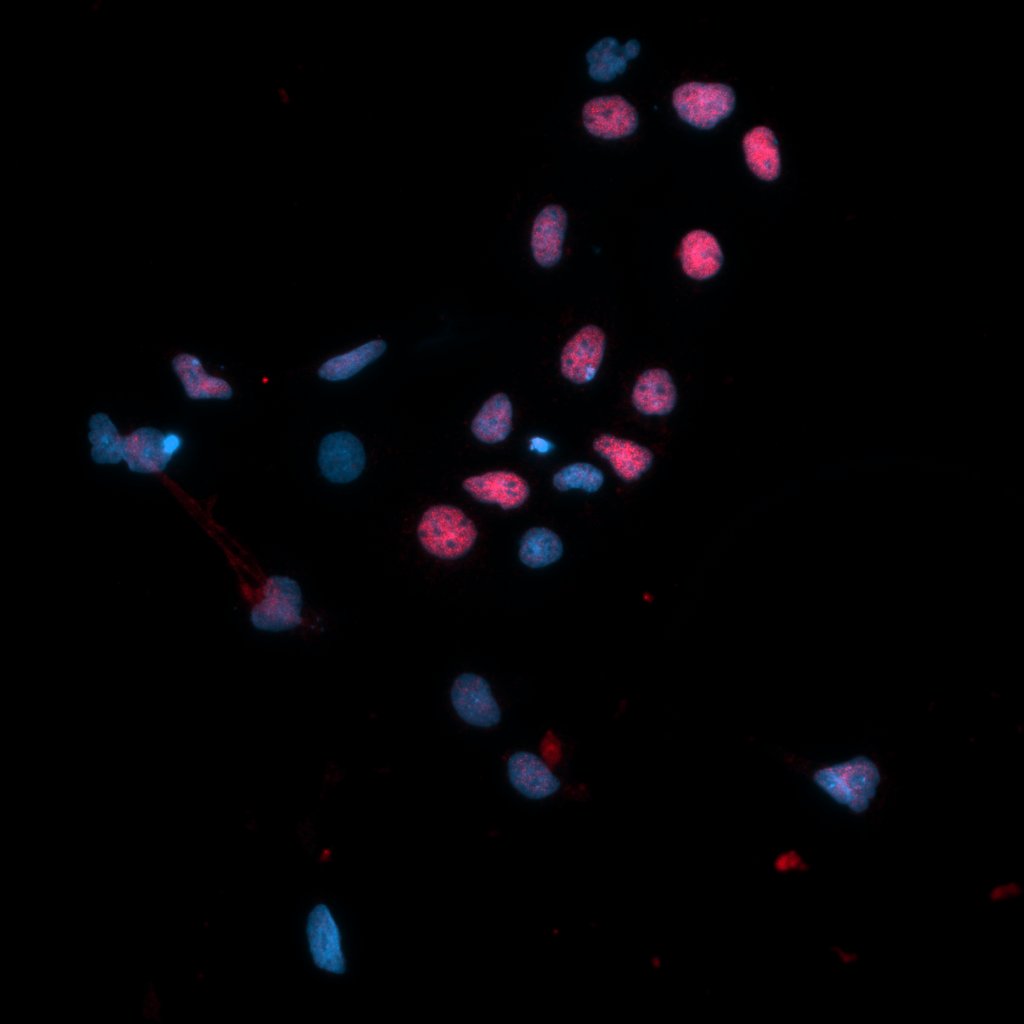

Supplement: Supplementary file 8 — Source data Fig. 1 [file 44320_2025_151_MOESM8_ESM.zip › FIGURE1/1E/242221-NCC-SOX9-TFAP2A/241121-NCC-paper-HSTE3-TFAP2A-40X-2-composite.jpg]

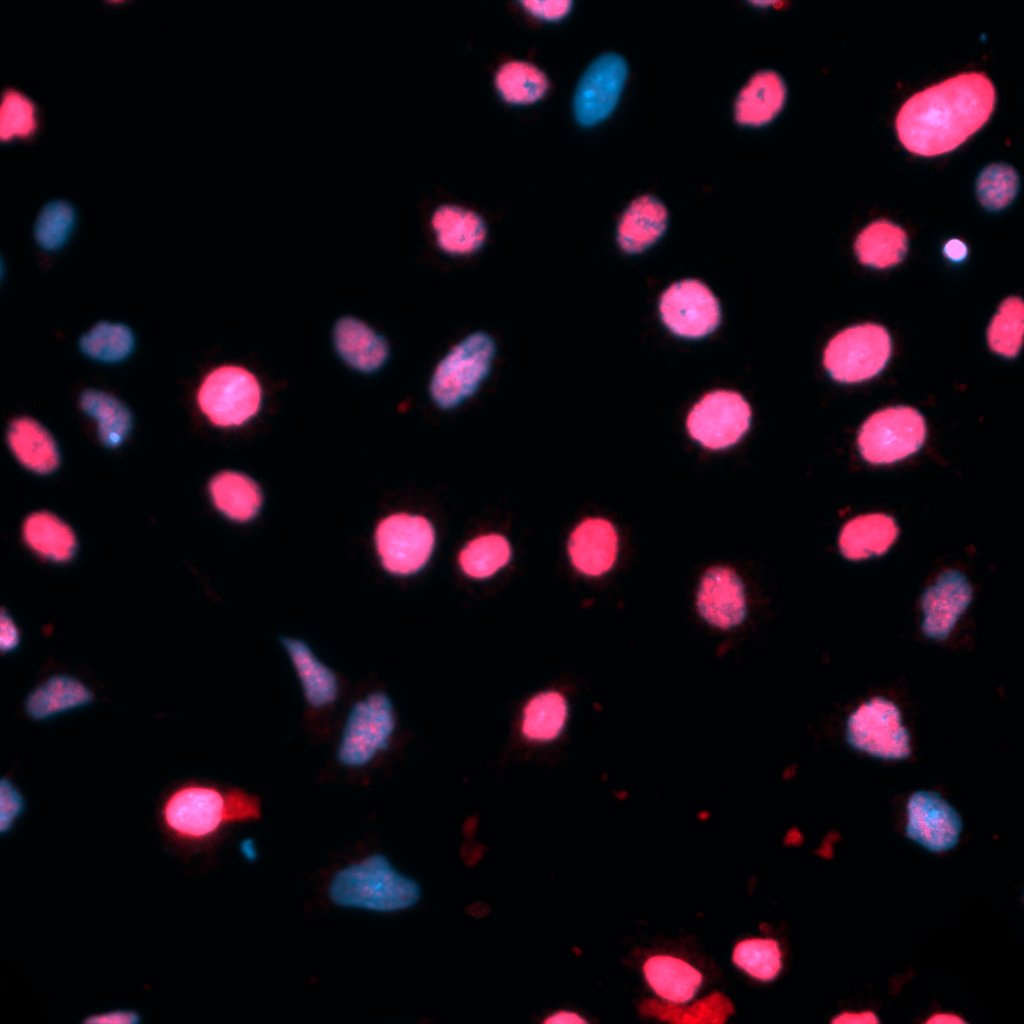

Supplement: Supplementary file 8 — Source data Fig. 1 [file 44320_2025_151_MOESM8_ESM.zip › FIGURE1/1E/242221-NCC-SOX9-TFAP2A/241121-NCC-paper-NOG3-TFAP2A-40X-paper.jpg]

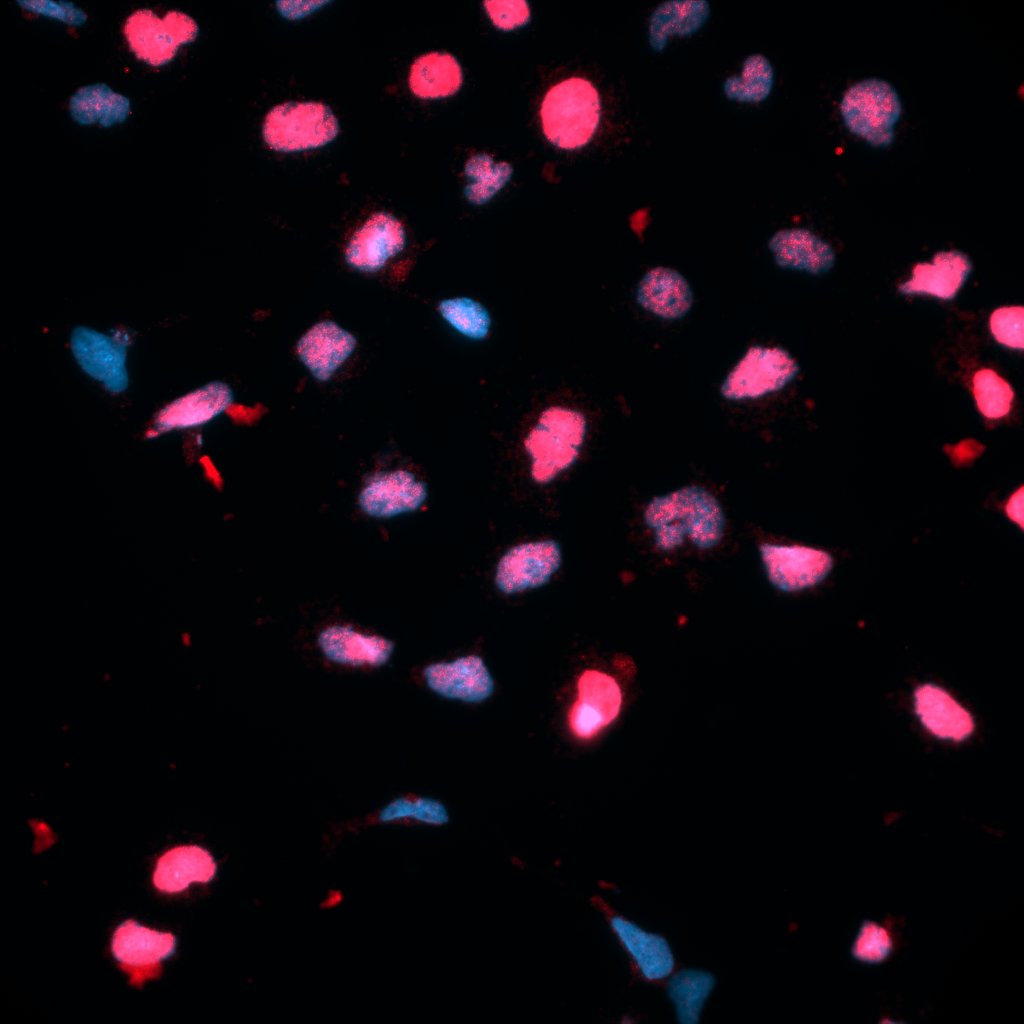

Supplement: Supplementary file 8 — Source data Fig. 1 [file 44320_2025_151_MOESM8_ESM.zip › FIGURE1/1E/242221-NCC-SOX9-TFAP2A/241121-NCC-paper-HSTE3-TFAP2A-40X-paper.jpg]

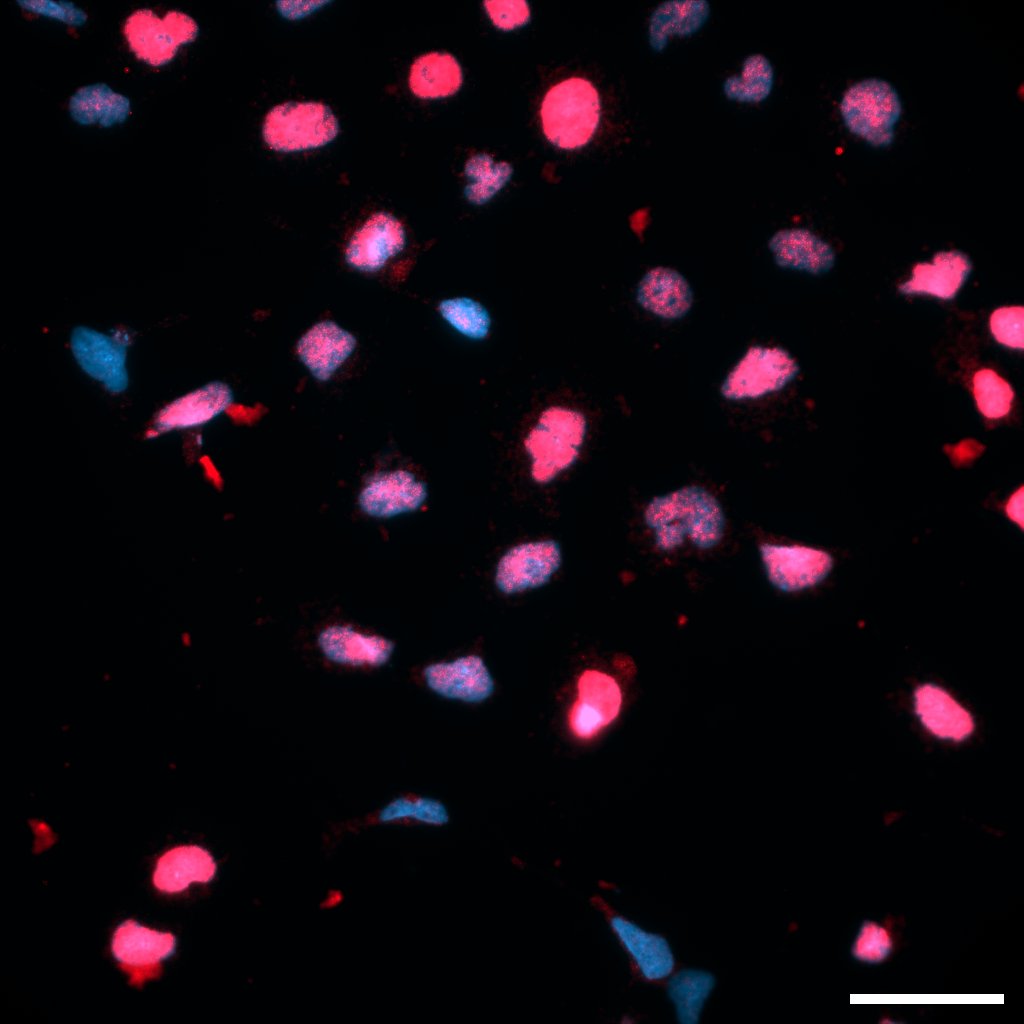

Supplement: Supplementary file 8 — Source data Fig. 1 [file 44320_2025_151_MOESM8_ESM.zip › FIGURE1/1E/242221-NCC-SOX9-TFAP2A/241121-NCC-paper-HSTE3-TFAP2A-40X-paper-scalebar.jpg]

## Slide 1
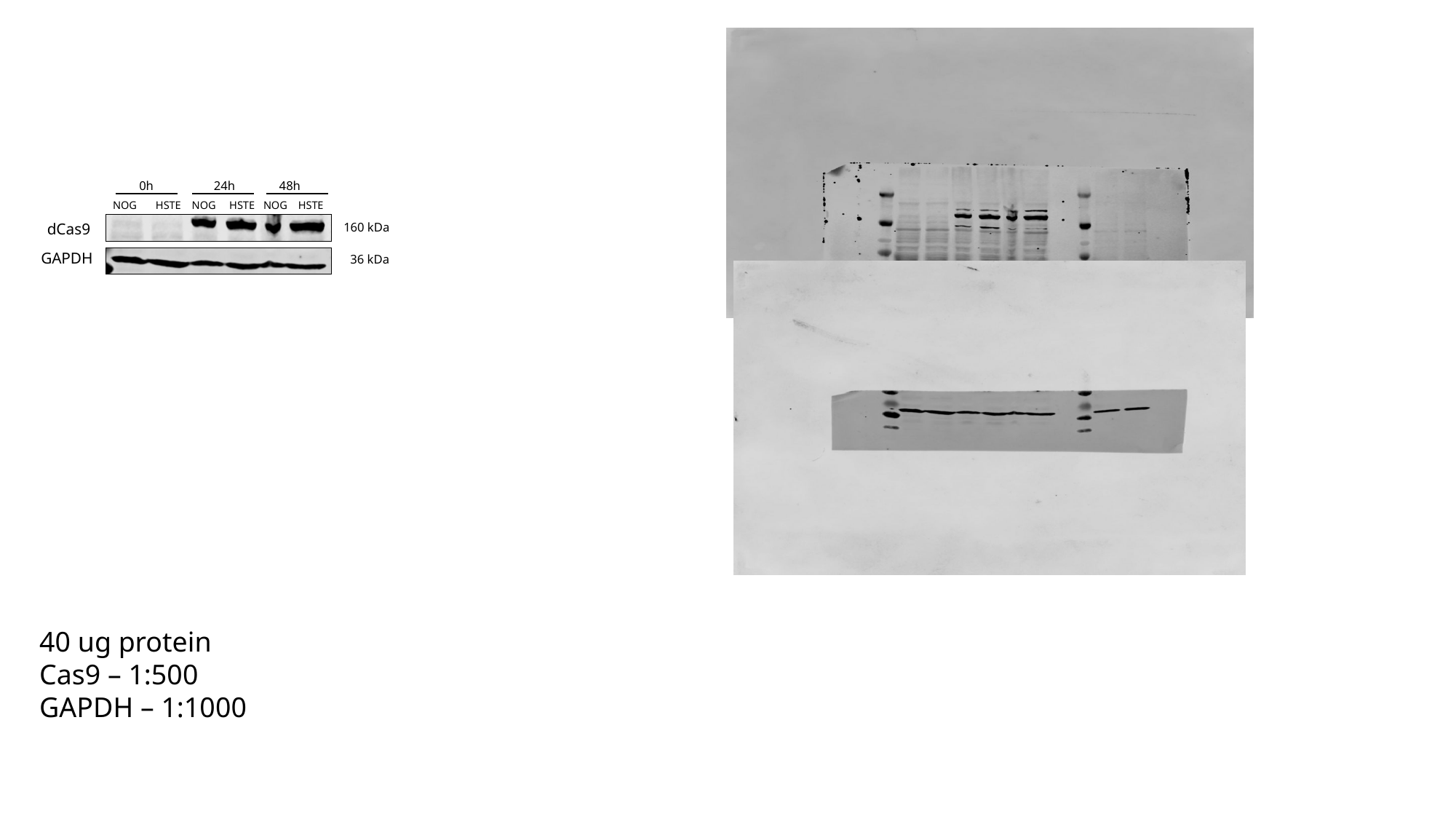

48h
0h
24h
NOG HSTE
NOG HSTE
NOG HSTE
dCas9
160 kDa
GAPDH
36 kDa
40 ug protein
Cas9 – 1:500
GAPDH – 1:1000

Supplement: Supplementary file 8 — Source data Fig. 1 [file 44320_2025_151_MOESM8_ESM.zip › FIGURE1/1D/240731-CNCC d1-3 and CO d55/Presentation1.pptx]

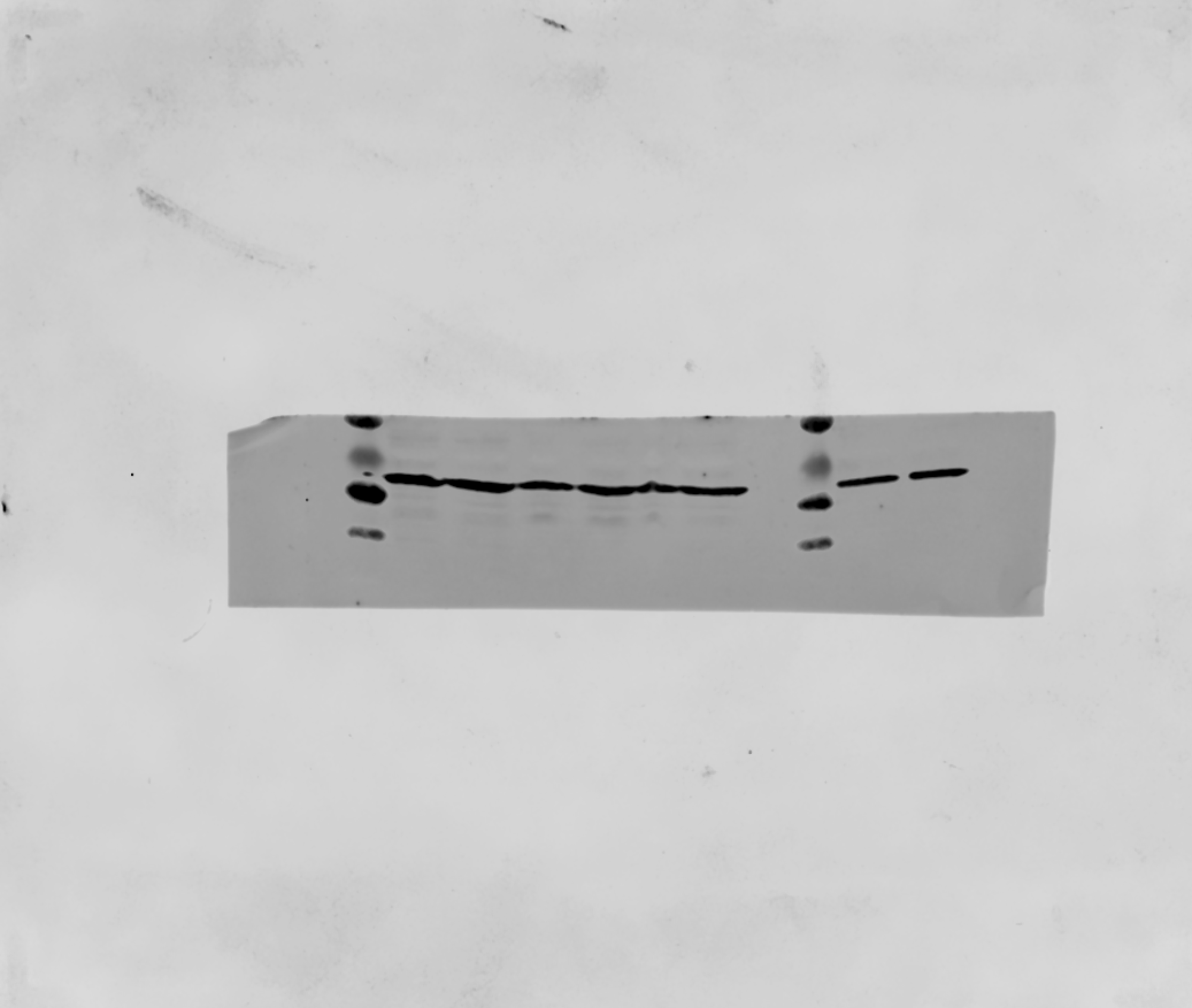

Supplement: Supplementary file 8 — Source data Fig. 1 [file 44320_2025_151_MOESM8_ESM.zip › FIGURE1/1D/240731-CNCC d1-3 and CO d55/LD_Cas9_CNCCs d1to3_CO d55_GAPDH.tif]

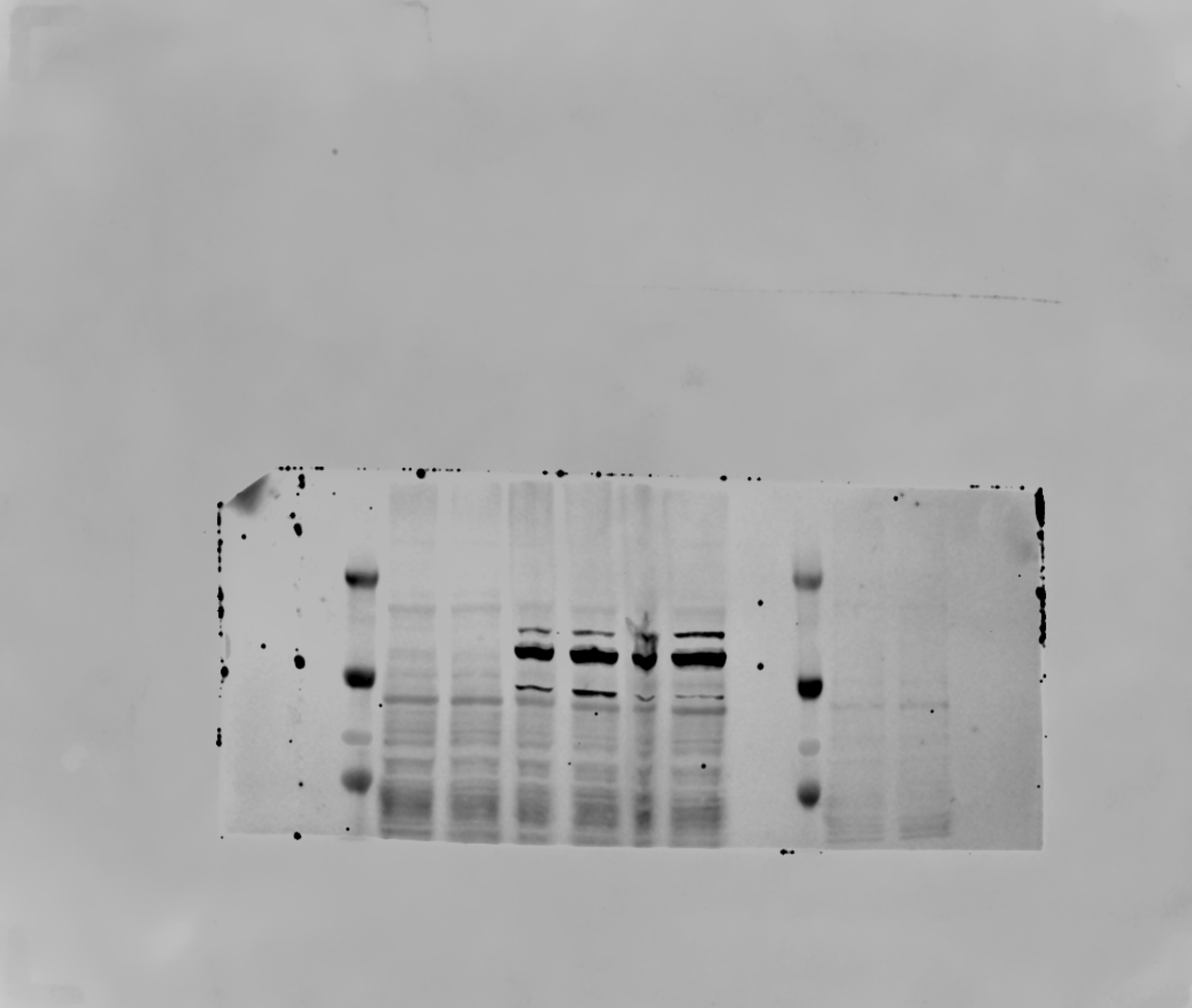

Supplement: Supplementary file 8 — Source data Fig. 1 [file 44320_2025_151_MOESM8_ESM.zip › FIGURE1/1D/240731-CNCC d1-3 and CO d55/LD_Cas9_CNCCs d1to3_CO d55_Cas9.tif]

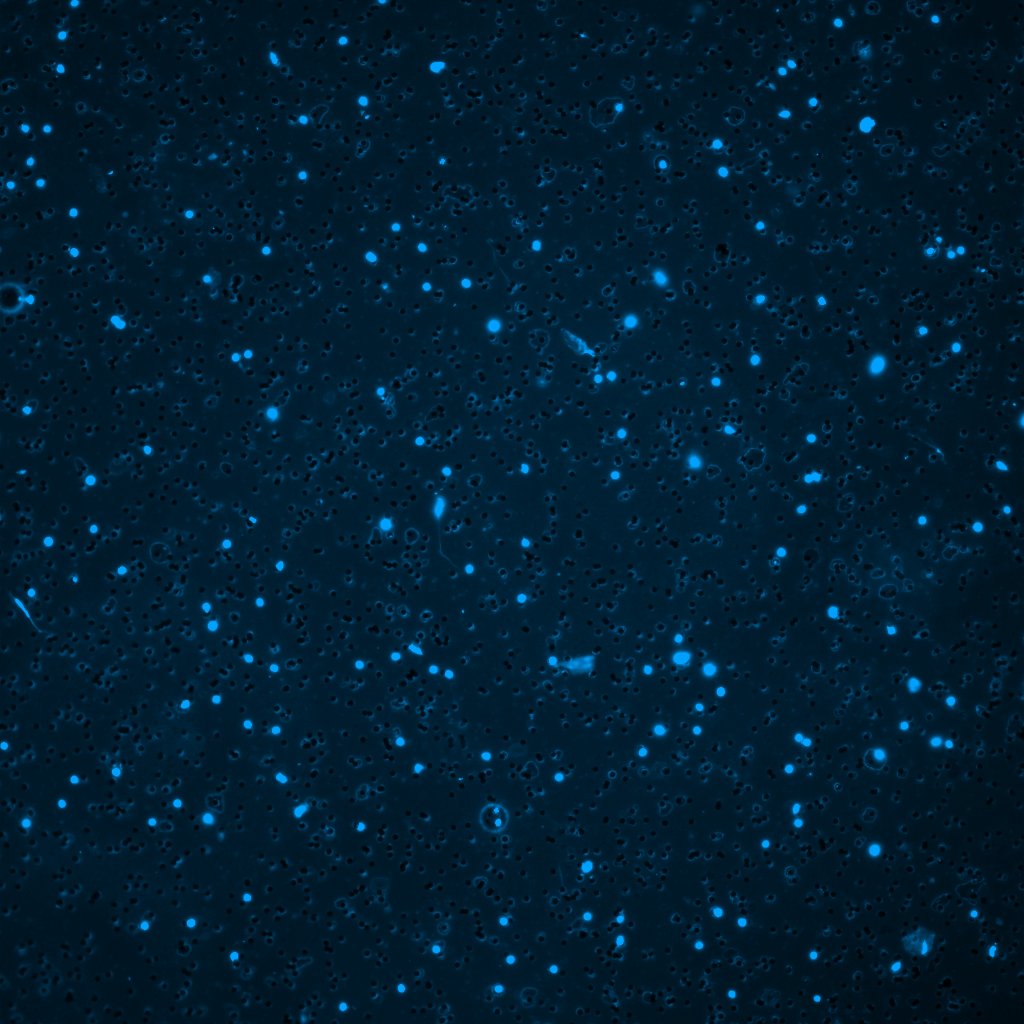

Supplement: Supplementary file 11 — Source data Fig. 4 [file 44320_2025_151_MOESM11_ESM.zip › FIGURE4/4B/250305-TWM-NOGvsHSTE-10pFBS/NOG5-24h.jpg]

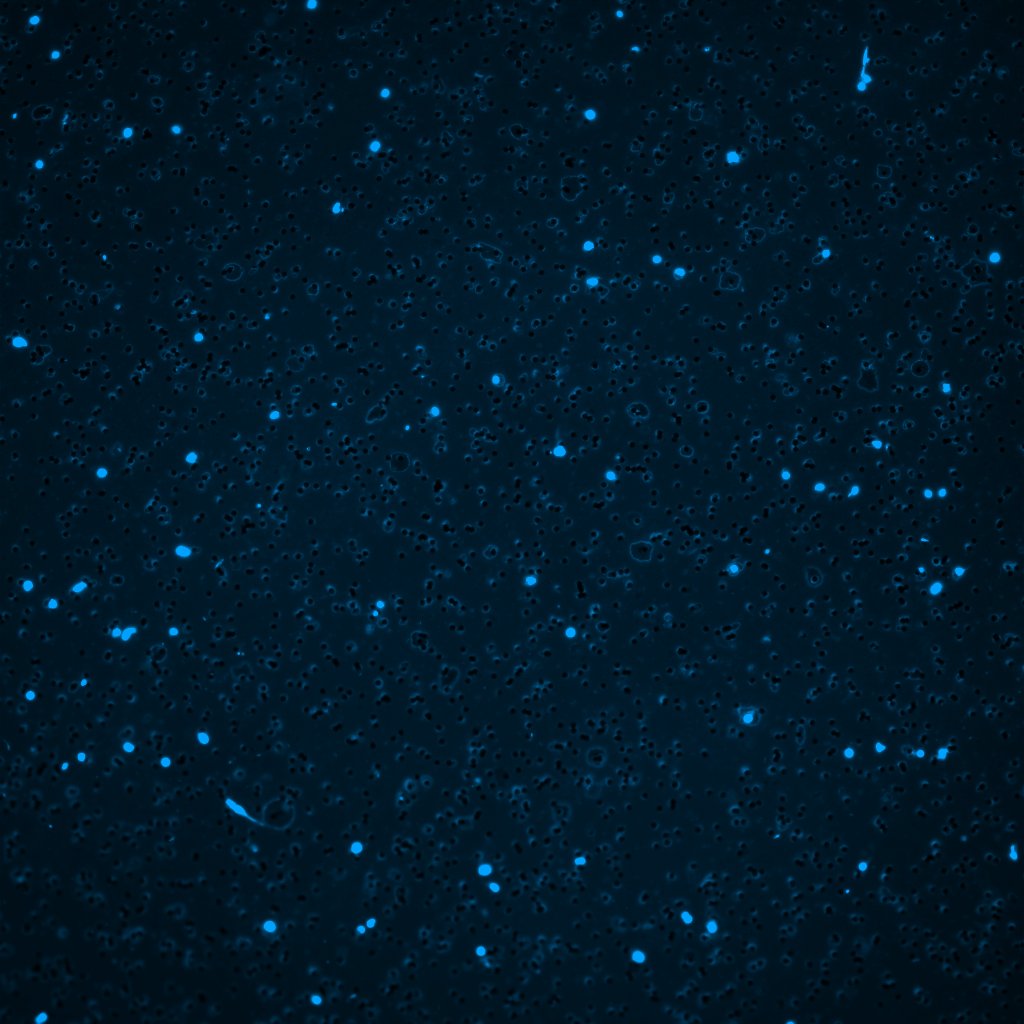

Supplement: Supplementary file 11 — Source data Fig. 4 [file 44320_2025_151_MOESM11_ESM.zip › FIGURE4/4B/250305-TWM-NOGvsHSTE-10pFBS/HSTE3-24h-3.jpg]

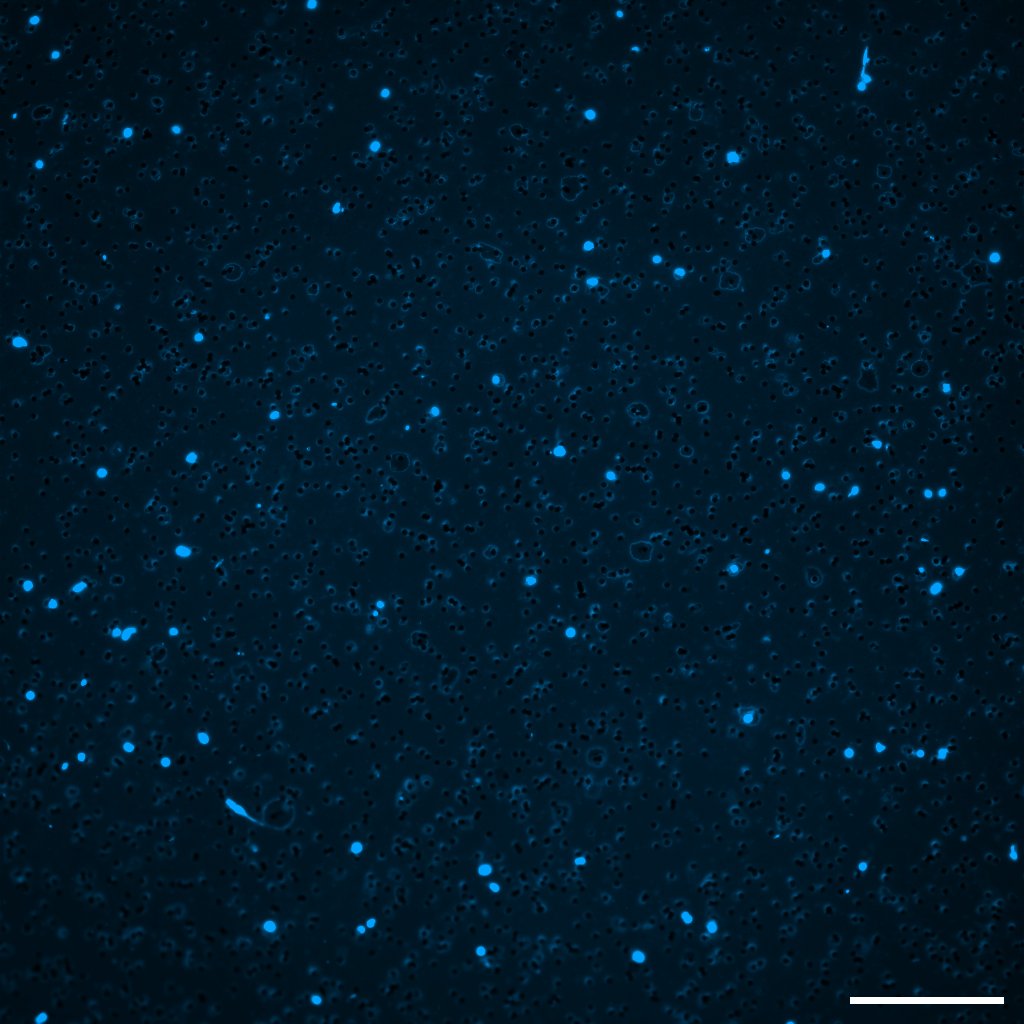

Supplement: Supplementary file 11 — Source data Fig. 4 [file 44320_2025_151_MOESM11_ESM.zip › FIGURE4/4B/250305-TWM-NOGvsHSTE-10pFBS/HSTE3-24h-3-scalebar.jpg]

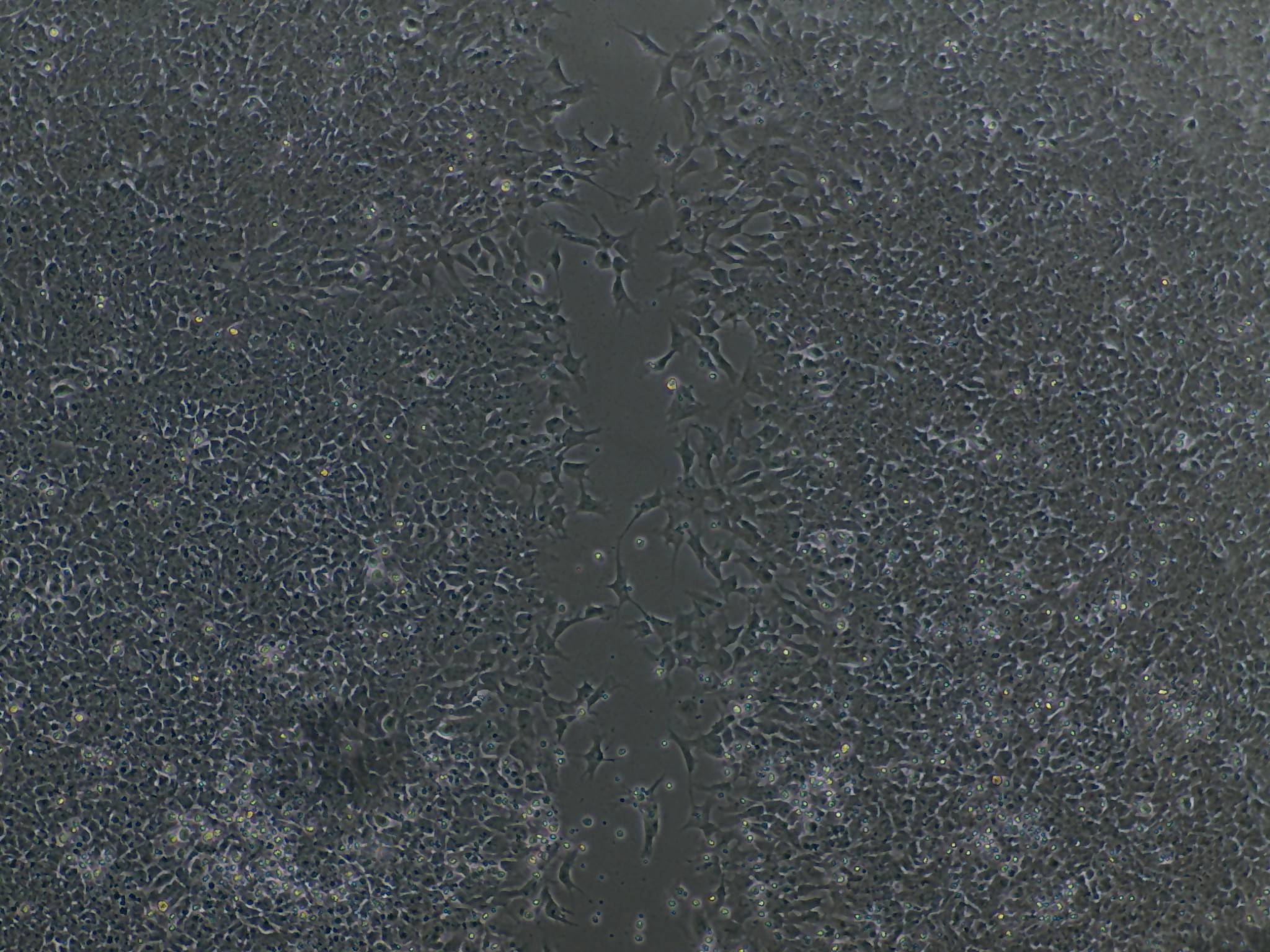

Supplement: Supplementary file 11 — Source data Fig. 4 [file 44320_2025_151_MOESM11_ESM.zip › FIGURE4/4D/250529-NCC-d5-SWA/NOG12-8h.jpg]

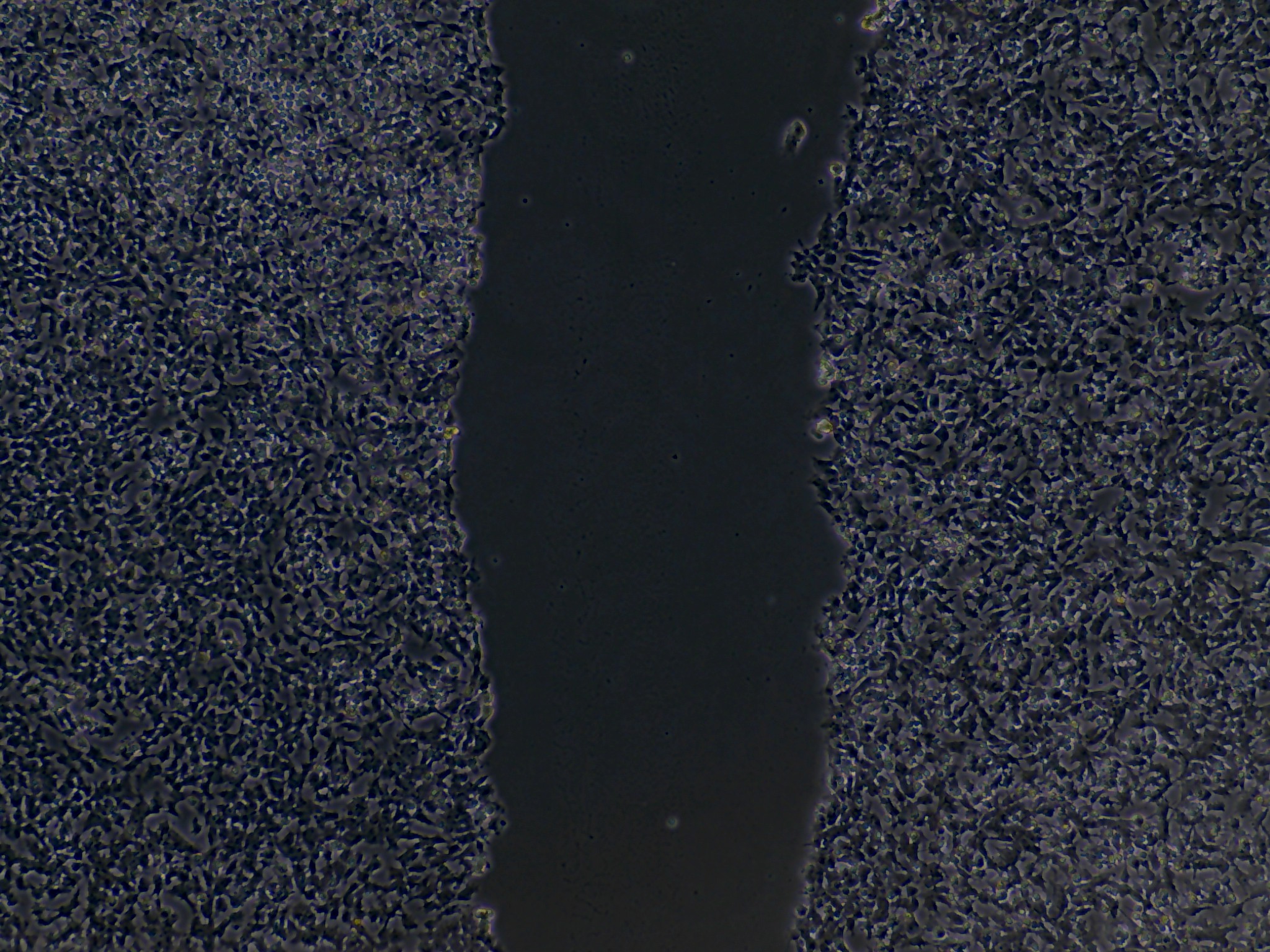

Supplement: Supplementary file 11 — Source data Fig. 4 [file 44320_2025_151_MOESM11_ESM.zip › FIGURE4/4D/250529-NCC-d5-SWA/NOG7-0h.jpg]

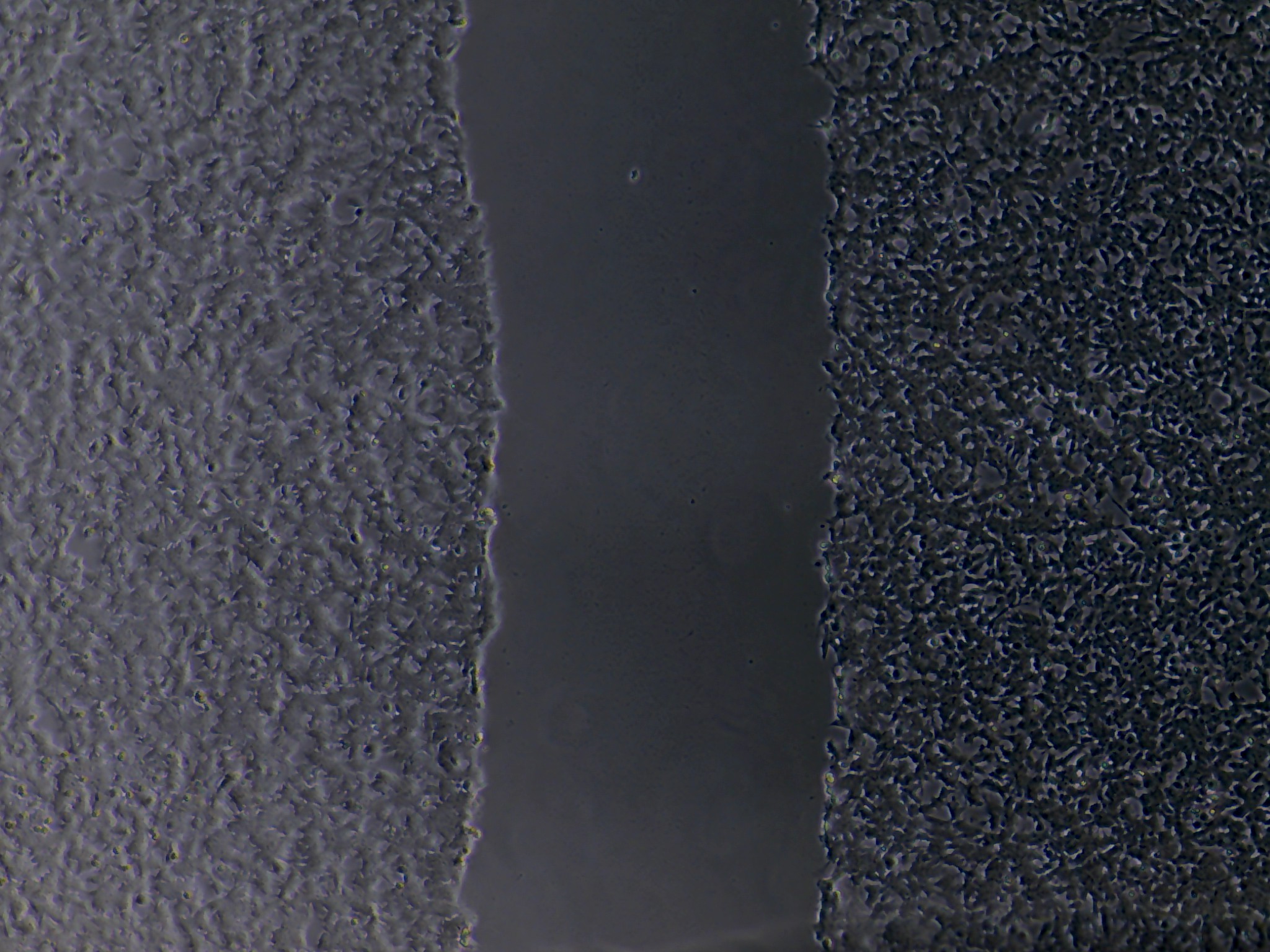

Supplement: Supplementary file 11 — Source data Fig. 4 [file 44320_2025_151_MOESM11_ESM.zip › FIGURE4/4D/250529-NCC-d5-SWA/HSTE6-0h.jpg]

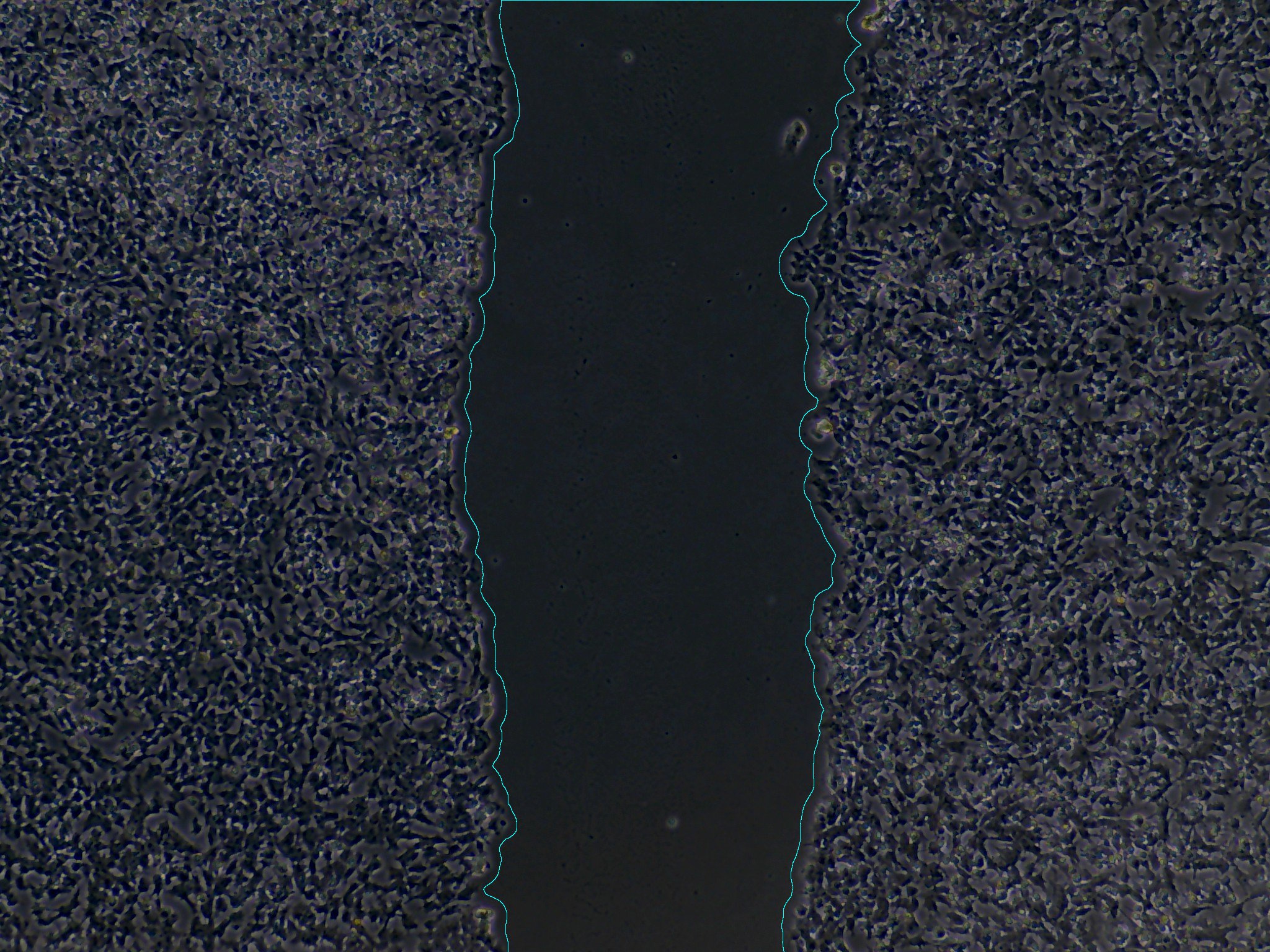

Supplement: Supplementary file 11 — Source data Fig. 4 [file 44320_2025_151_MOESM11_ESM.zip › FIGURE4/4D/250529-NCC-d5-SWA/NOG7-0h-1-flatten.jpg]

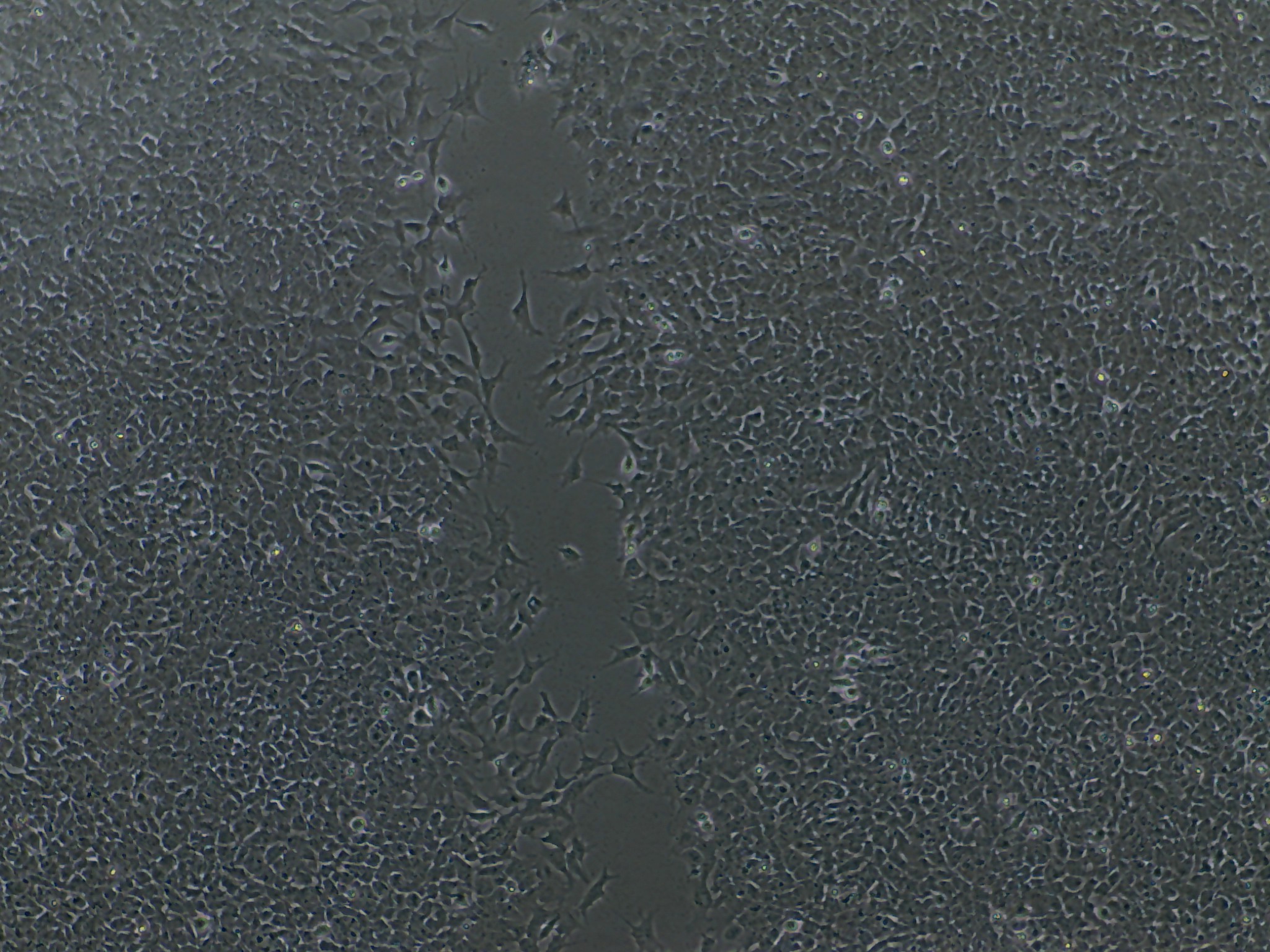

Supplement: Supplementary file 11 — Source data Fig. 4 [file 44320_2025_151_MOESM11_ESM.zip › FIGURE4/4D/250529-NCC-d5-SWA/NOG6-8h.jpg]

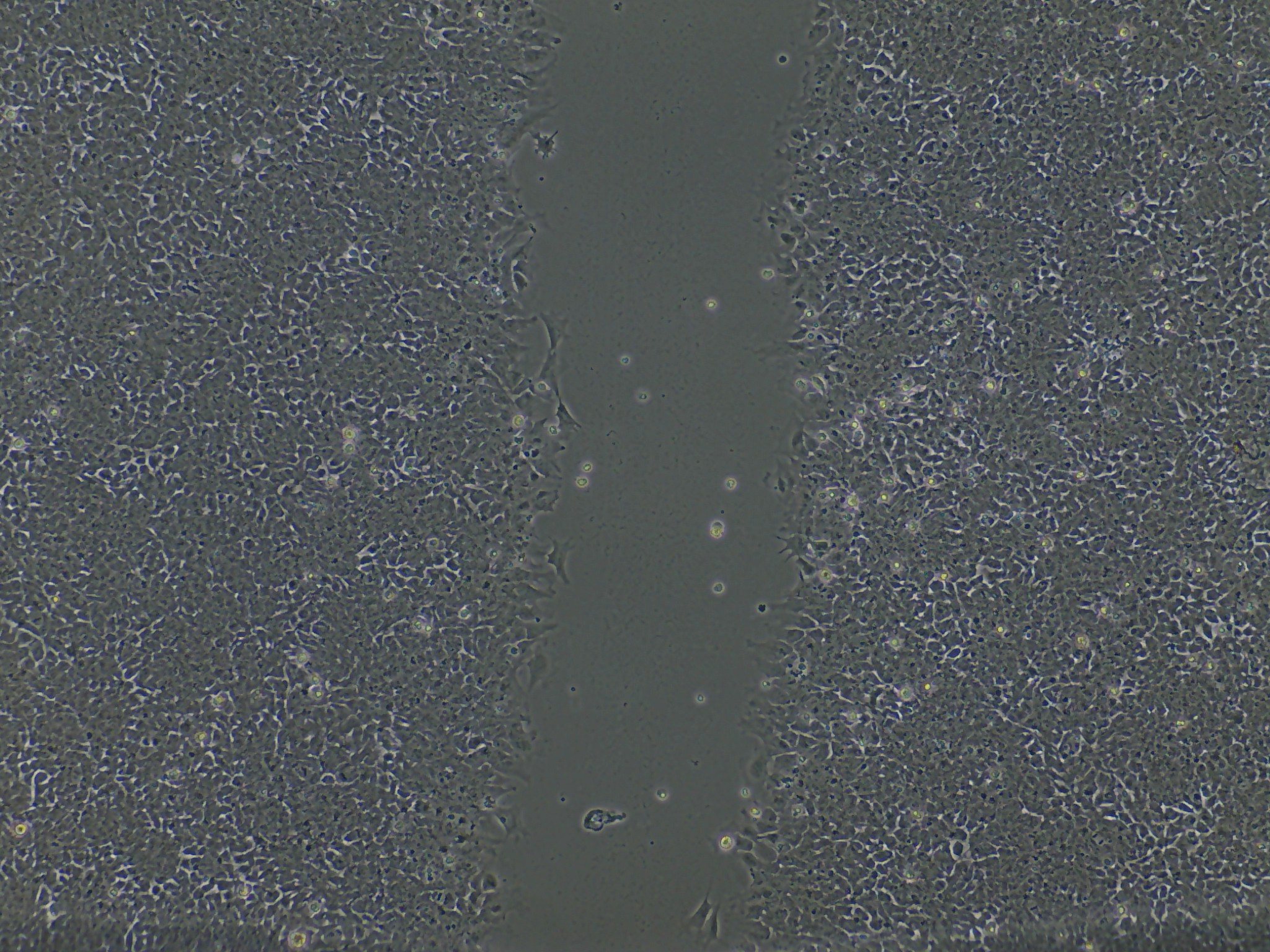

Supplement: Supplementary file 11 — Source data Fig. 4 [file 44320_2025_151_MOESM11_ESM.zip › FIGURE4/4D/250529-NCC-d5-SWA/HSTE7-8h.jpg]

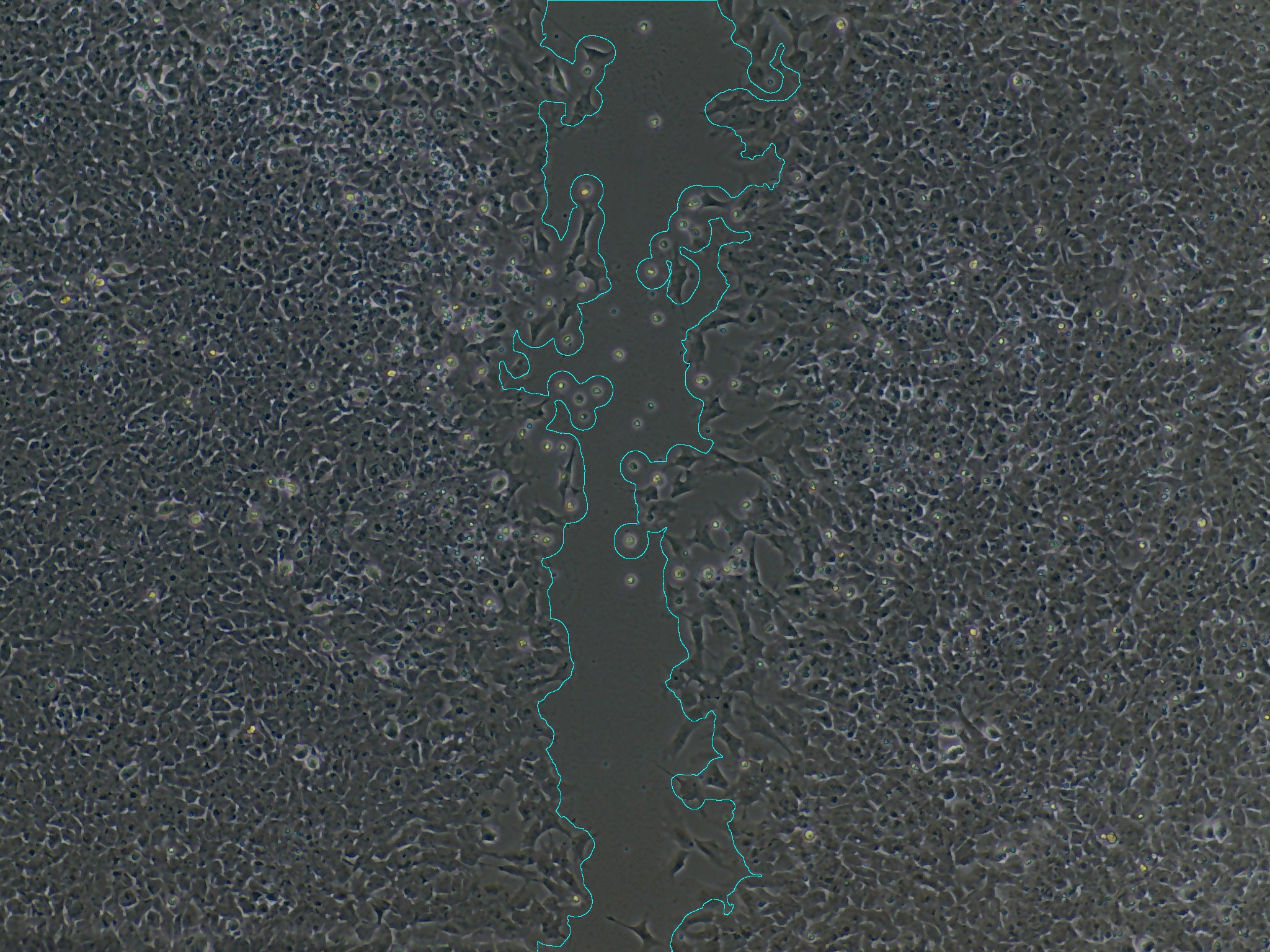

Supplement: Supplementary file 11 — Source data Fig. 4 [file 44320_2025_151_MOESM11_ESM.zip › FIGURE4/4D/250529-NCC-d5-SWA/NOG7-8h-1-flatten.jpg]

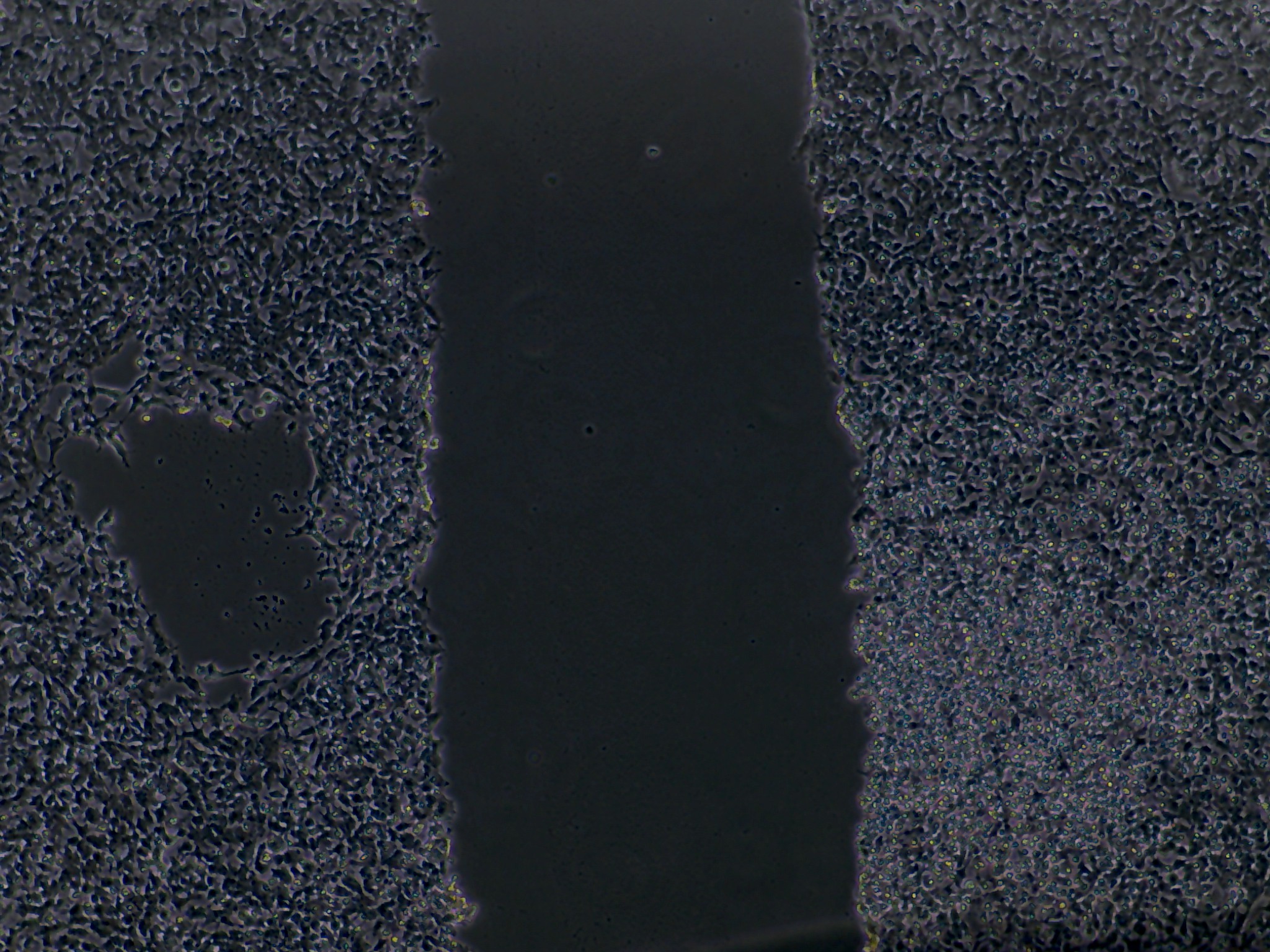

Supplement: Supplementary file 11 — Source data Fig. 4 [file 44320_2025_151_MOESM11_ESM.zip › FIGURE4/4D/250529-NCC-d5-SWA/NOG11-0h.jpg]

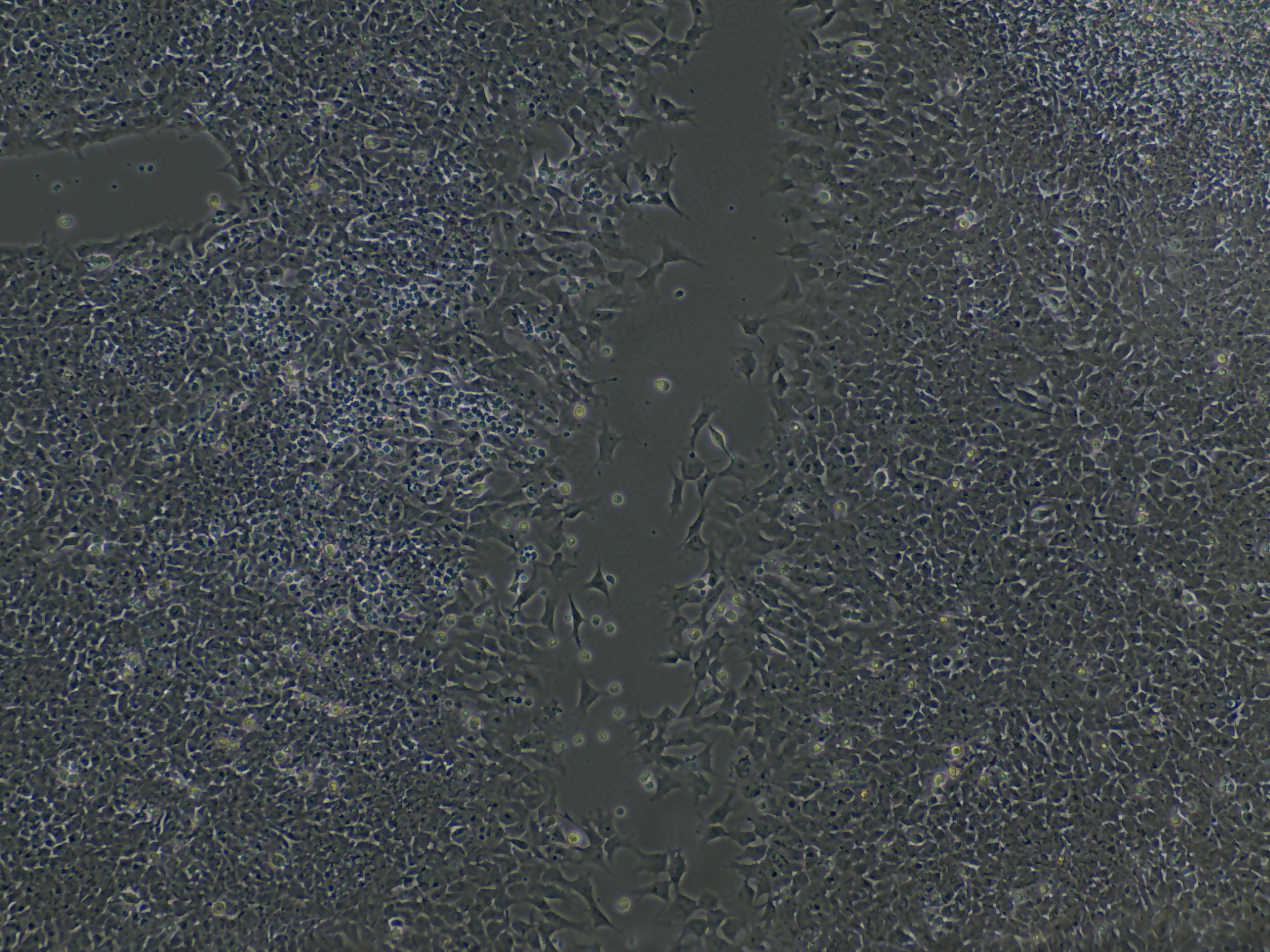

Supplement: Supplementary file 11 — Source data Fig. 4 [file 44320_2025_151_MOESM11_ESM.zip › FIGURE4/4D/250529-NCC-d5-SWA/NOG8-8h.jpg]

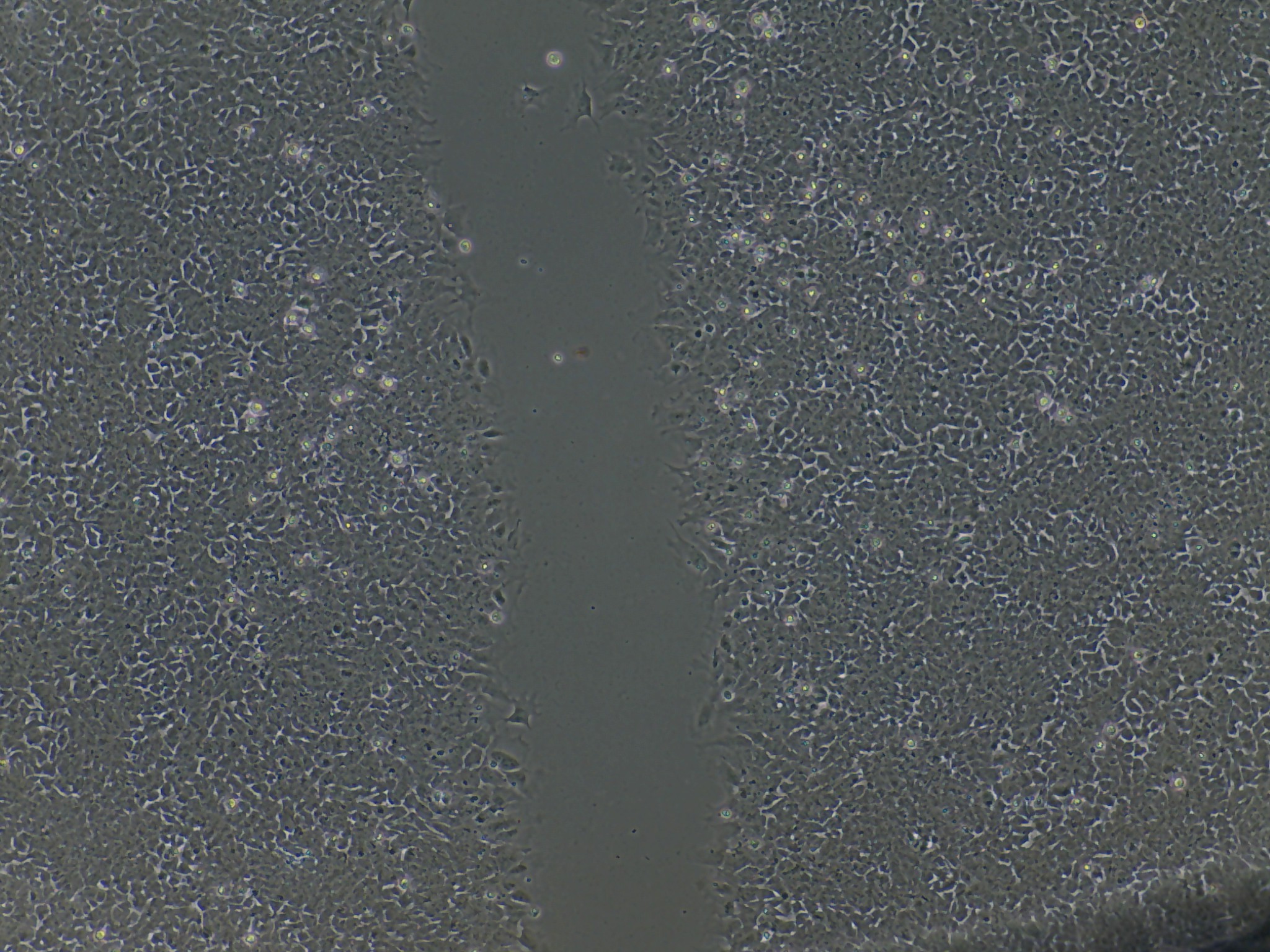

Supplement: Supplementary file 11 — Source data Fig. 4 [file 44320_2025_151_MOESM11_ESM.zip › FIGURE4/4D/250529-NCC-d5-SWA/HSTE9-8h.jpg]

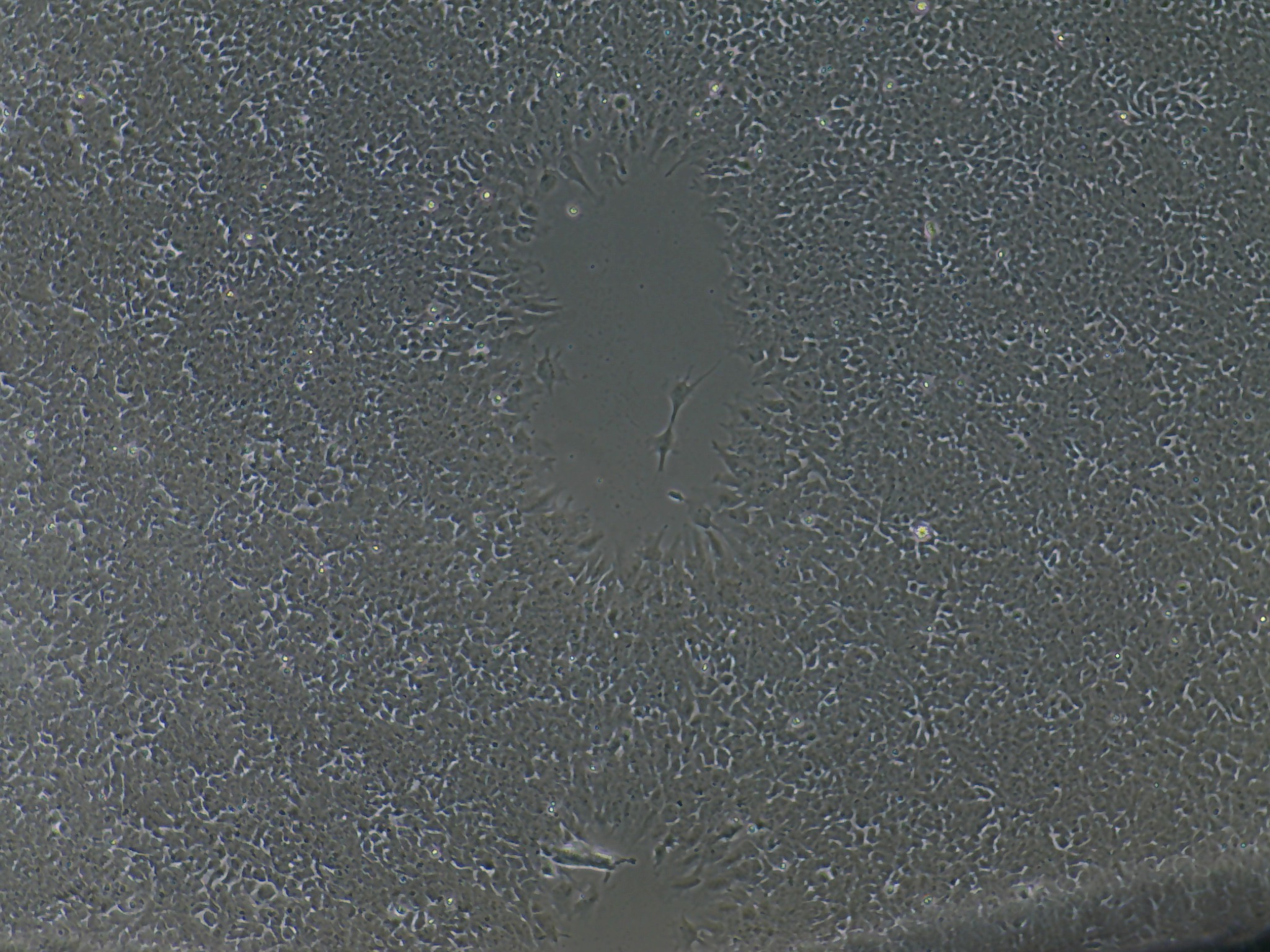

Supplement: Supplementary file 11 — Source data Fig. 4 [file 44320_2025_151_MOESM11_ESM.zip › FIGURE4/4D/250529-NCC-d5-SWA/HSTE5-8h.jpg]

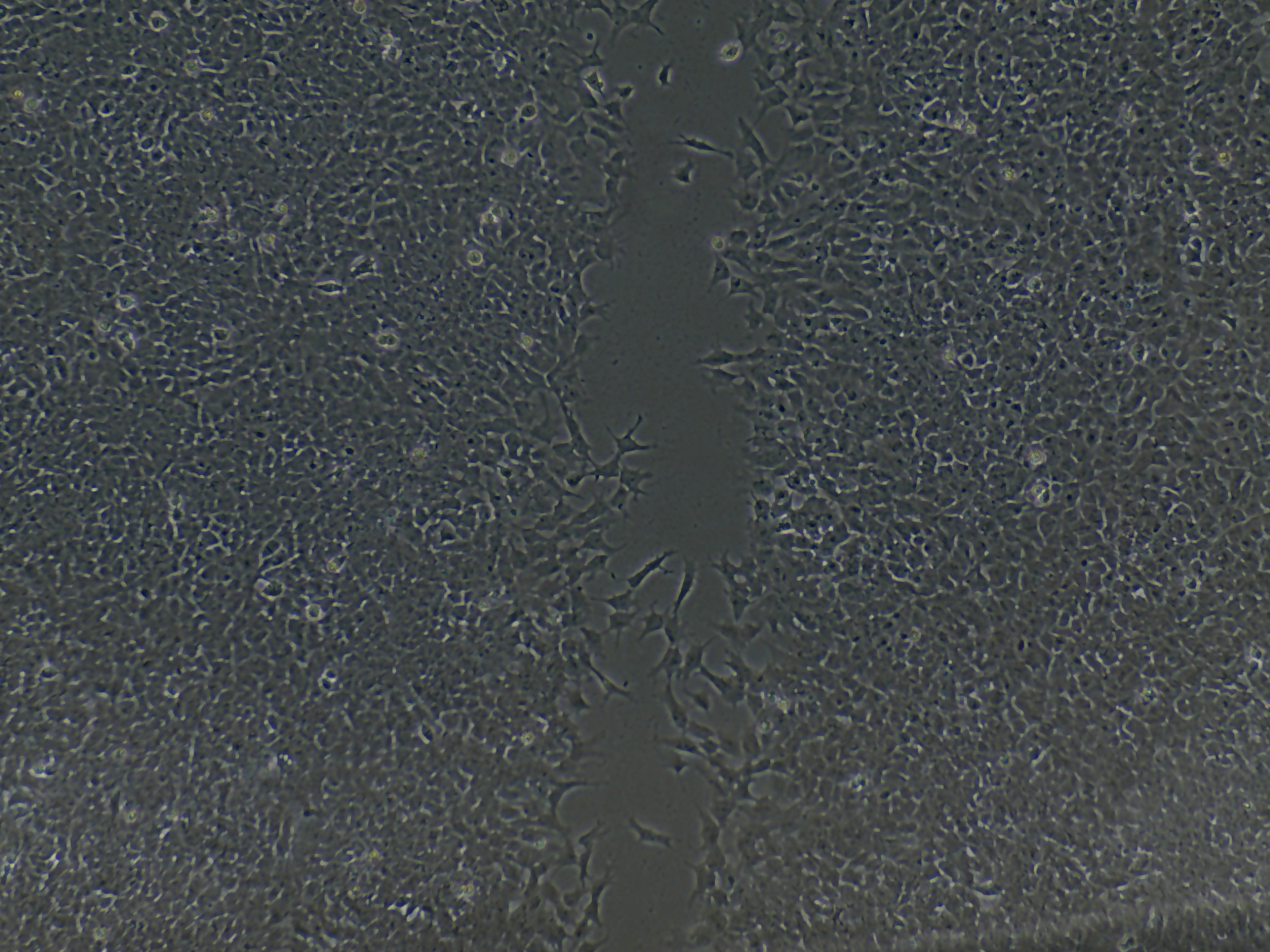

Supplement: Supplementary file 11 — Source data Fig. 4 [file 44320_2025_151_MOESM11_ESM.zip › FIGURE4/4D/250529-NCC-d5-SWA/NOG4-8h.jpg]

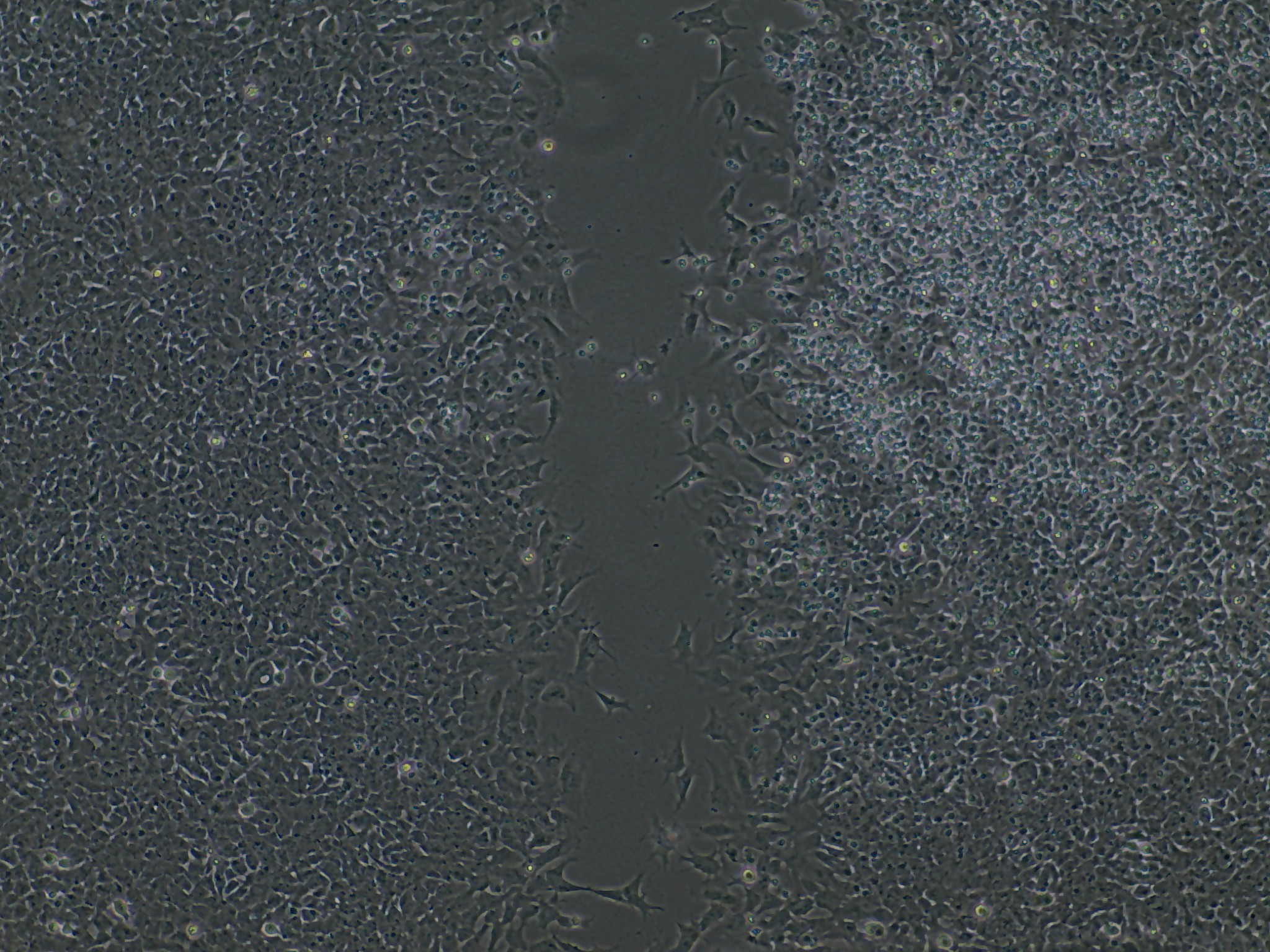

Supplement: Supplementary file 11 — Source data Fig. 4 [file 44320_2025_151_MOESM11_ESM.zip › FIGURE4/4D/250529-NCC-d5-SWA/NOG10-8h.jpg]

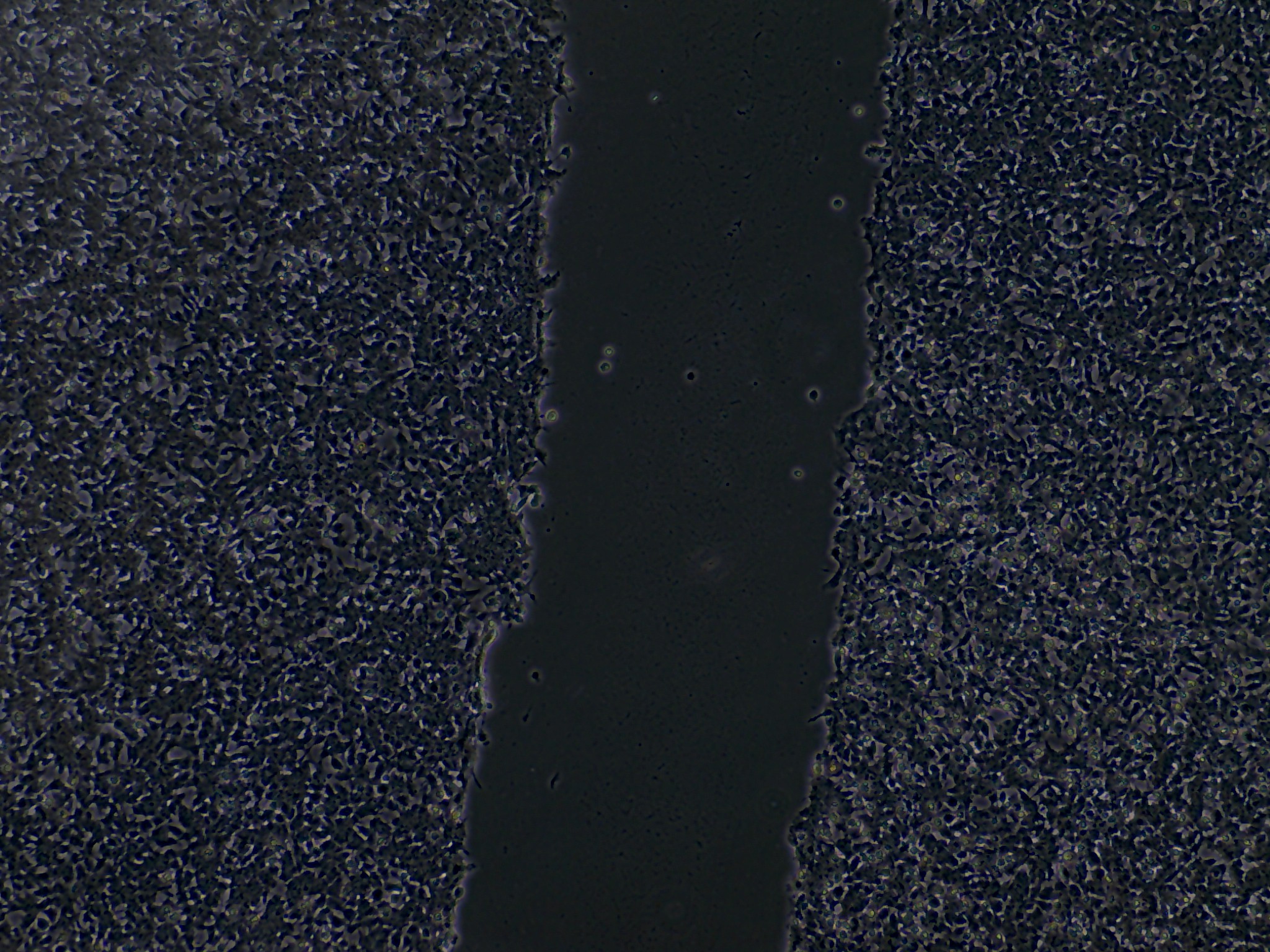

Supplement: Supplementary file 11 — Source data Fig. 4 [file 44320_2025_151_MOESM11_ESM.zip › FIGURE4/4D/250529-NCC-d5-SWA/HSTE4-0h.jpg]

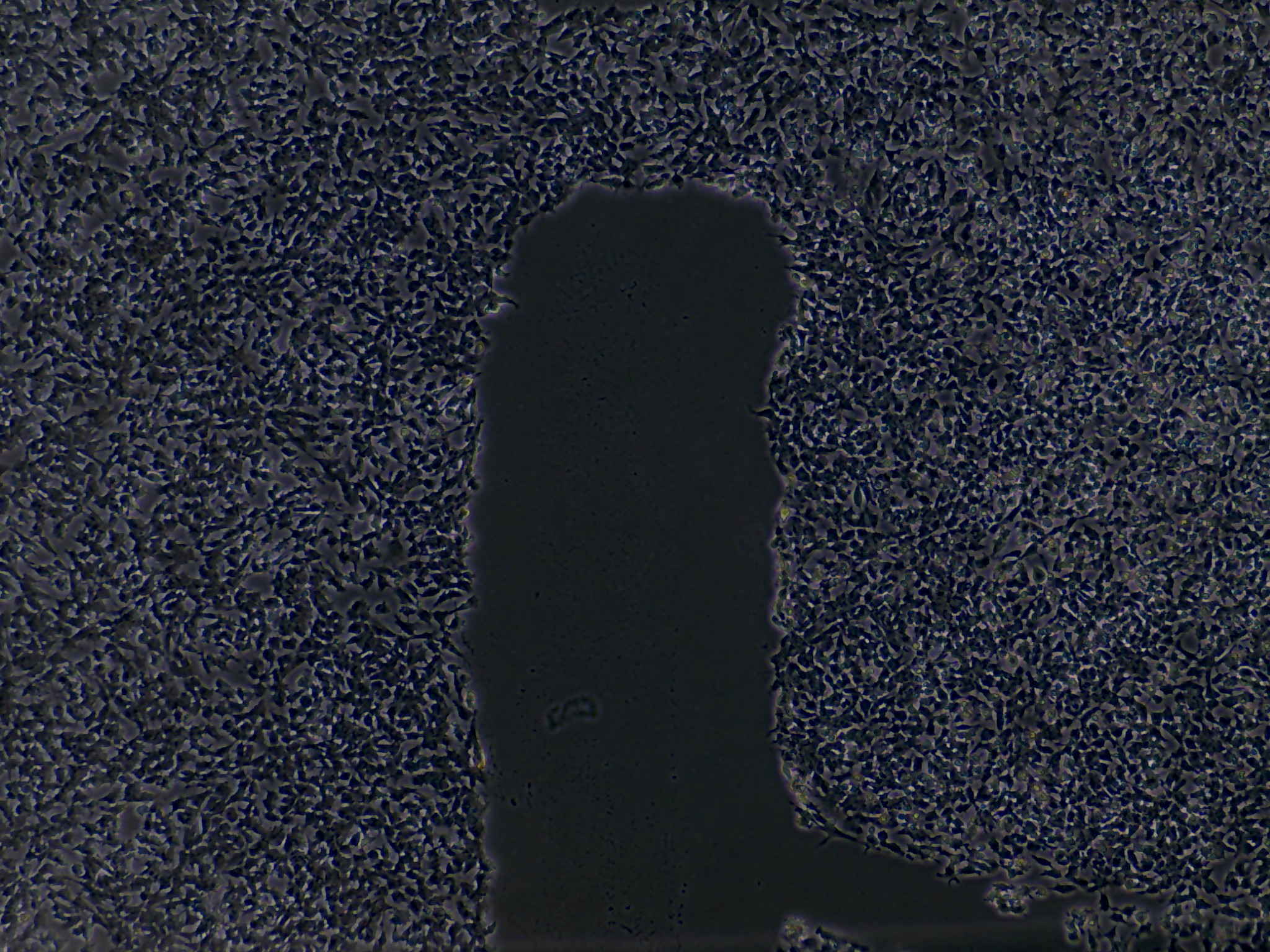

Supplement: Supplementary file 11 — Source data Fig. 4 [file 44320_2025_151_MOESM11_ESM.zip › FIGURE4/4D/250529-NCC-d5-SWA/NOG5-0h.jpg]

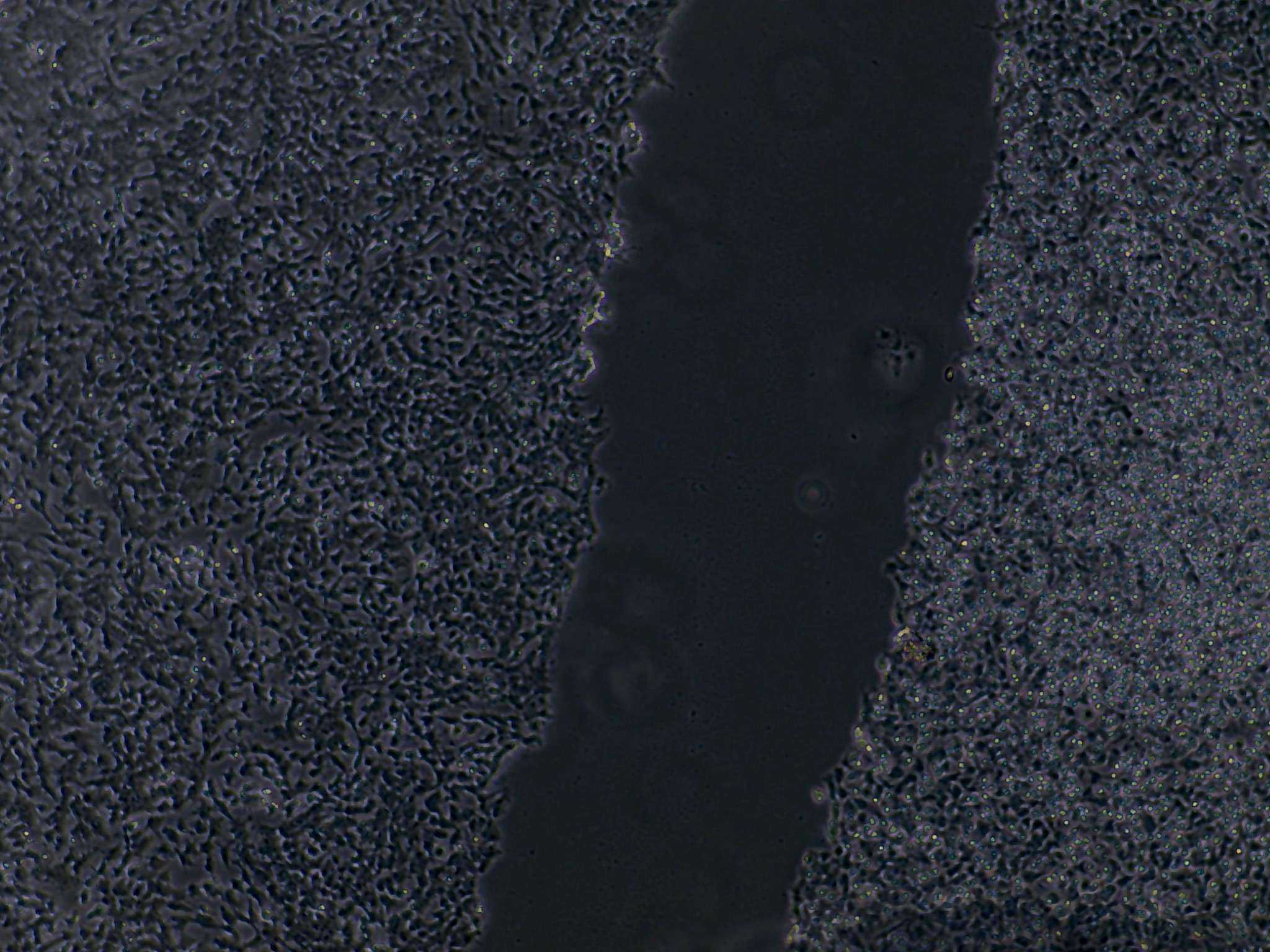

Supplement: Supplementary file 11 — Source data Fig. 4 [file 44320_2025_151_MOESM11_ESM.zip › FIGURE4/4D/250529-NCC-d5-SWA/NOG9-0h.jpg]

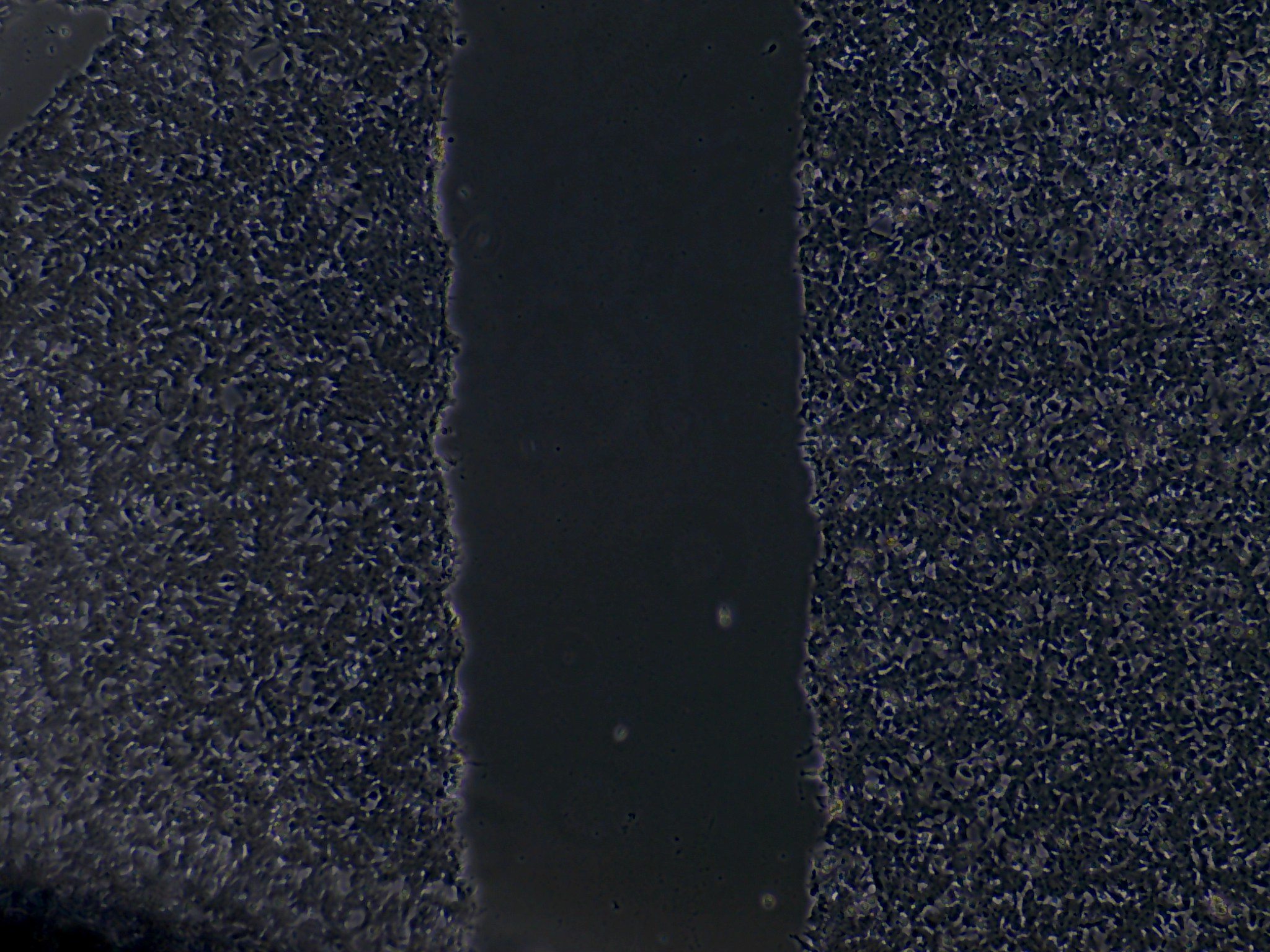

Supplement: Supplementary file 11 — Source data Fig. 4 [file 44320_2025_151_MOESM11_ESM.zip › FIGURE4/4D/250529-NCC-d5-SWA/HSTE8-0h.jpg]

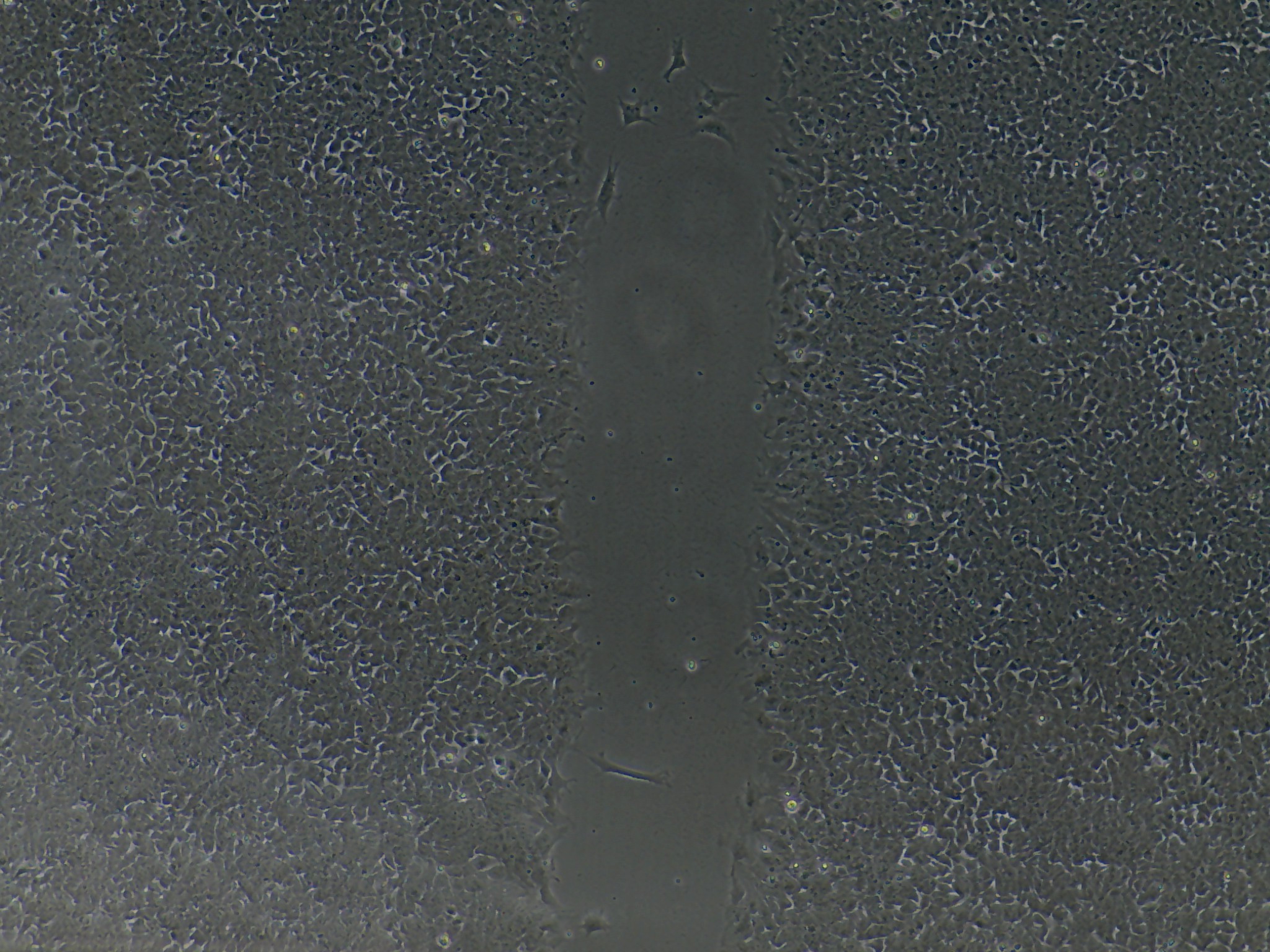

Supplement: Supplementary file 11 — Source data Fig. 4 [file 44320_2025_151_MOESM11_ESM.zip › FIGURE4/4D/250529-NCC-d5-SWA/HSTE1-8h.jpg]

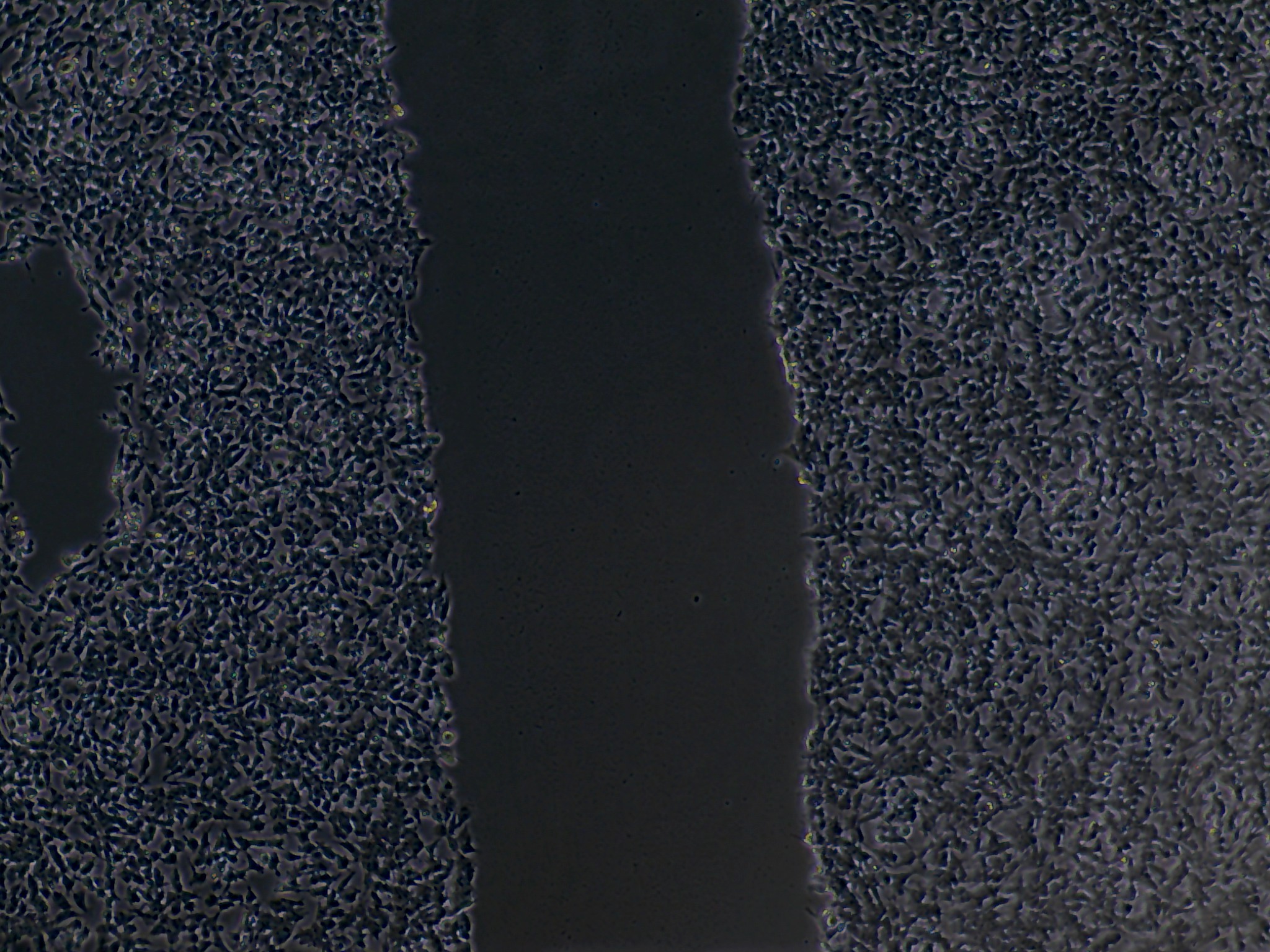

Supplement: Supplementary file 11 — Source data Fig. 4 [file 44320_2025_151_MOESM11_ESM.zip › FIGURE4/4D/250529-NCC-d5-SWA/NOG1-0h.jpg]

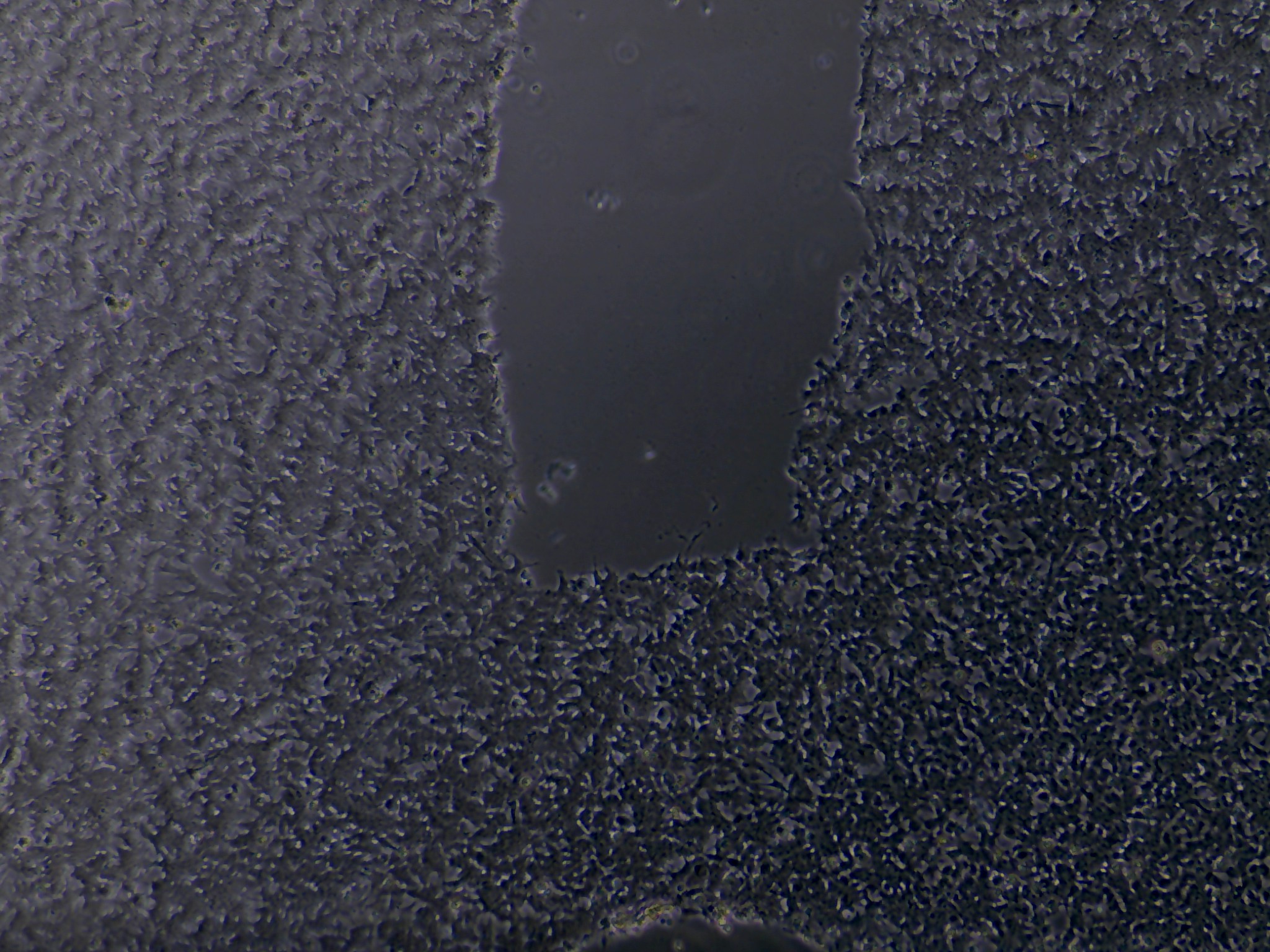

Supplement: Supplementary file 11 — Source data Fig. 4 [file 44320_2025_151_MOESM11_ESM.zip › FIGURE4/4D/250529-NCC-d5-SWA/HSTE12-0h.jpg]

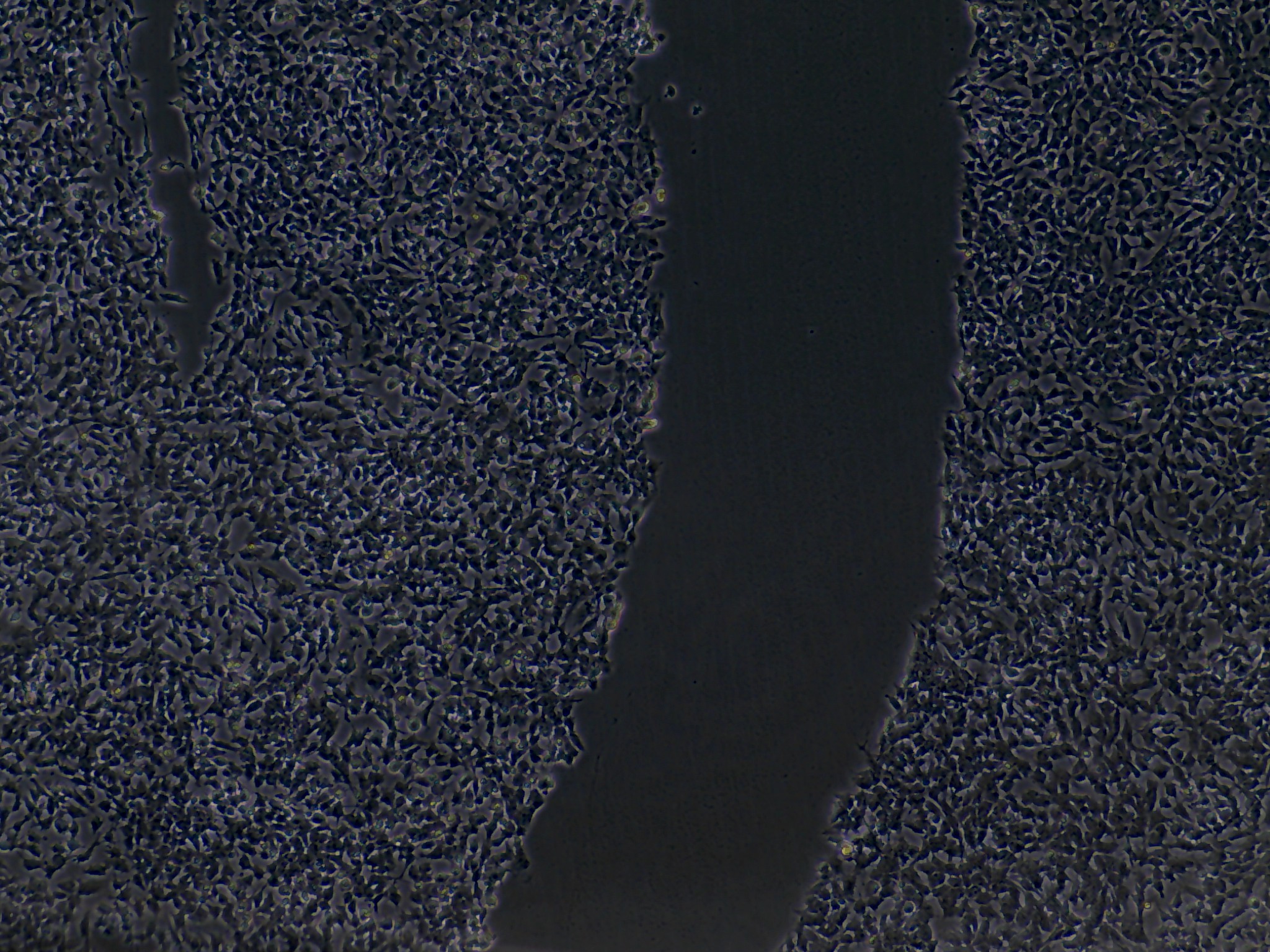

Supplement: Supplementary file 11 — Source data Fig. 4 [file 44320_2025_151_MOESM11_ESM.zip › FIGURE4/4D/250529-NCC-d5-SWA/NOG3-0h.jpg]

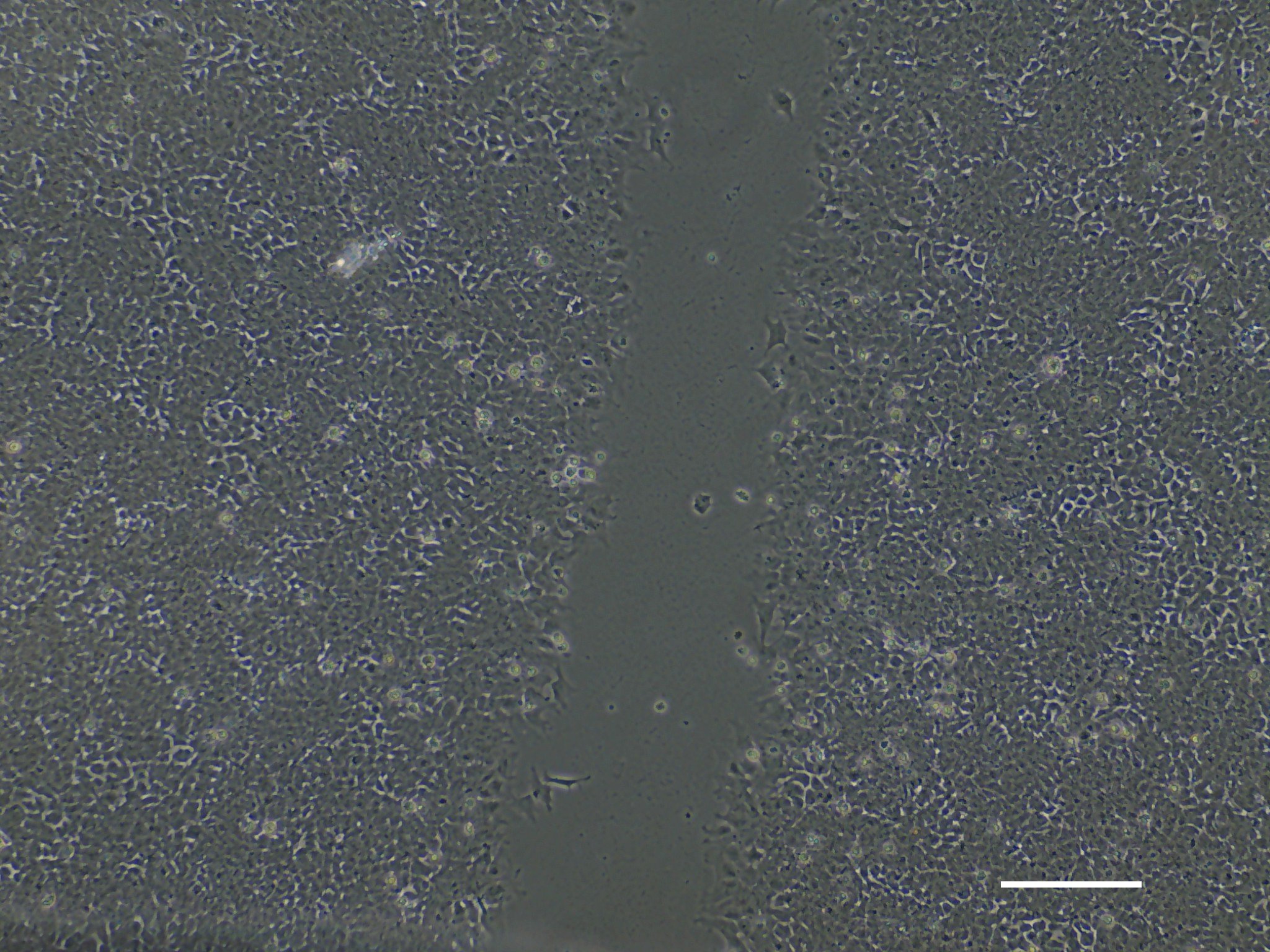

Supplement: Supplementary file 11 — Source data Fig. 4 [file 44320_2025_151_MOESM11_ESM.zip › FIGURE4/4D/250529-NCC-d5-SWA/HSTE4-8h-scalebar200um.jpg]

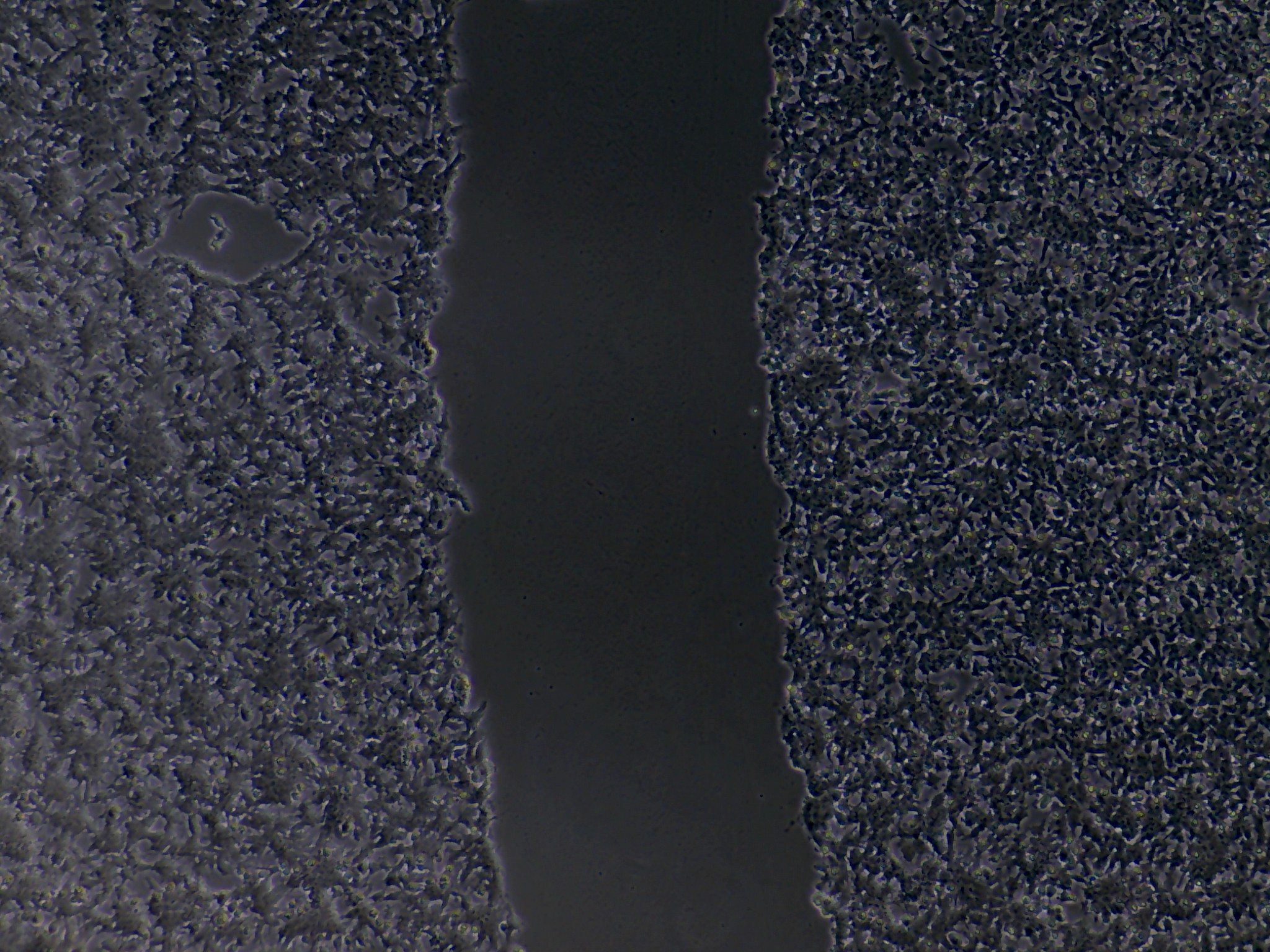

Supplement: Supplementary file 11 — Source data Fig. 4 [file 44320_2025_151_MOESM11_ESM.zip › FIGURE4/4D/250529-NCC-d5-SWA/HSTE2-0h.jpg]

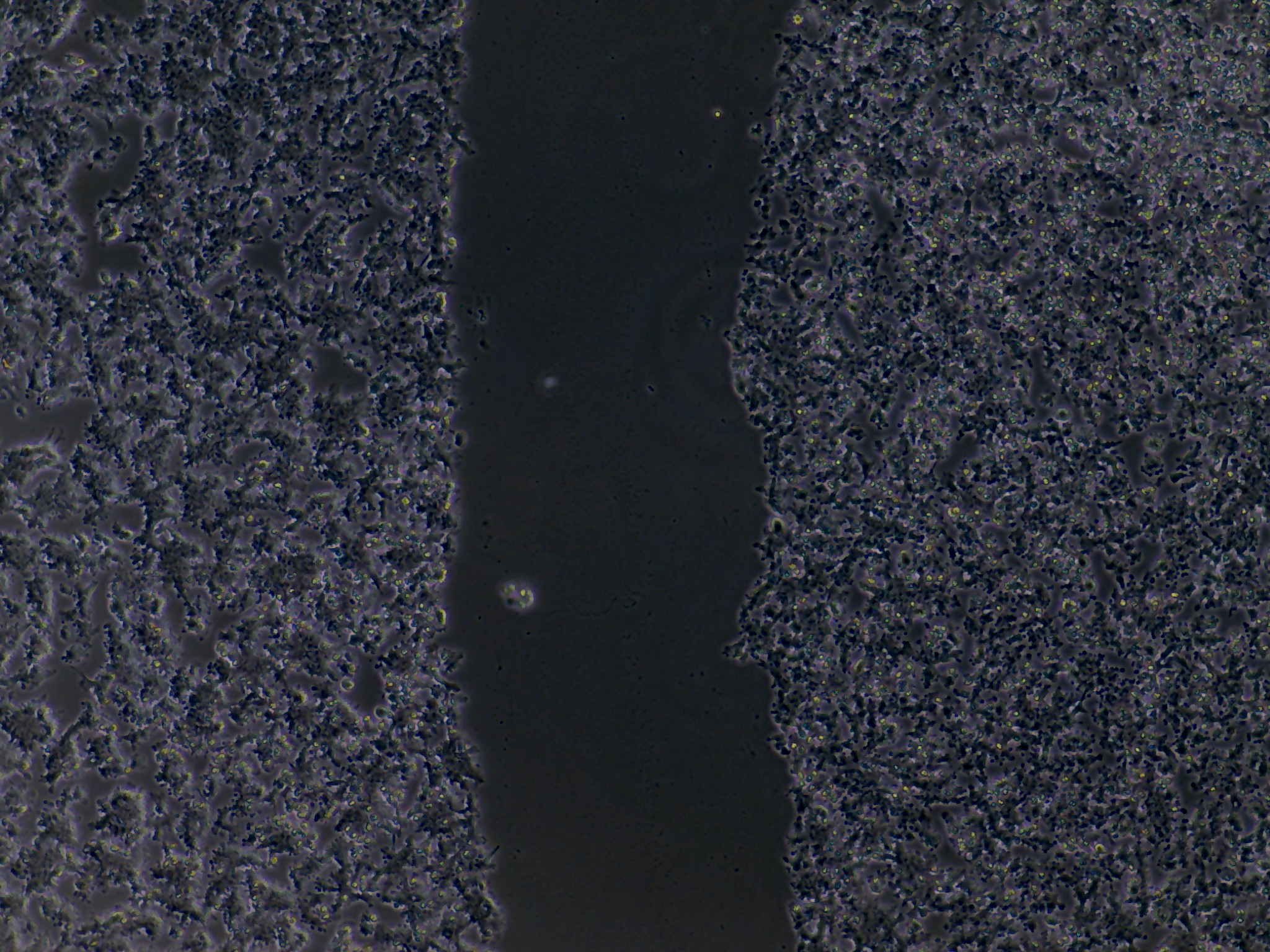

Supplement: Supplementary file 11 — Source data Fig. 4 [file 44320_2025_151_MOESM11_ESM.zip › FIGURE4/4D/250529-NCC-d5-SWA/HSTE10-0h.jpg]

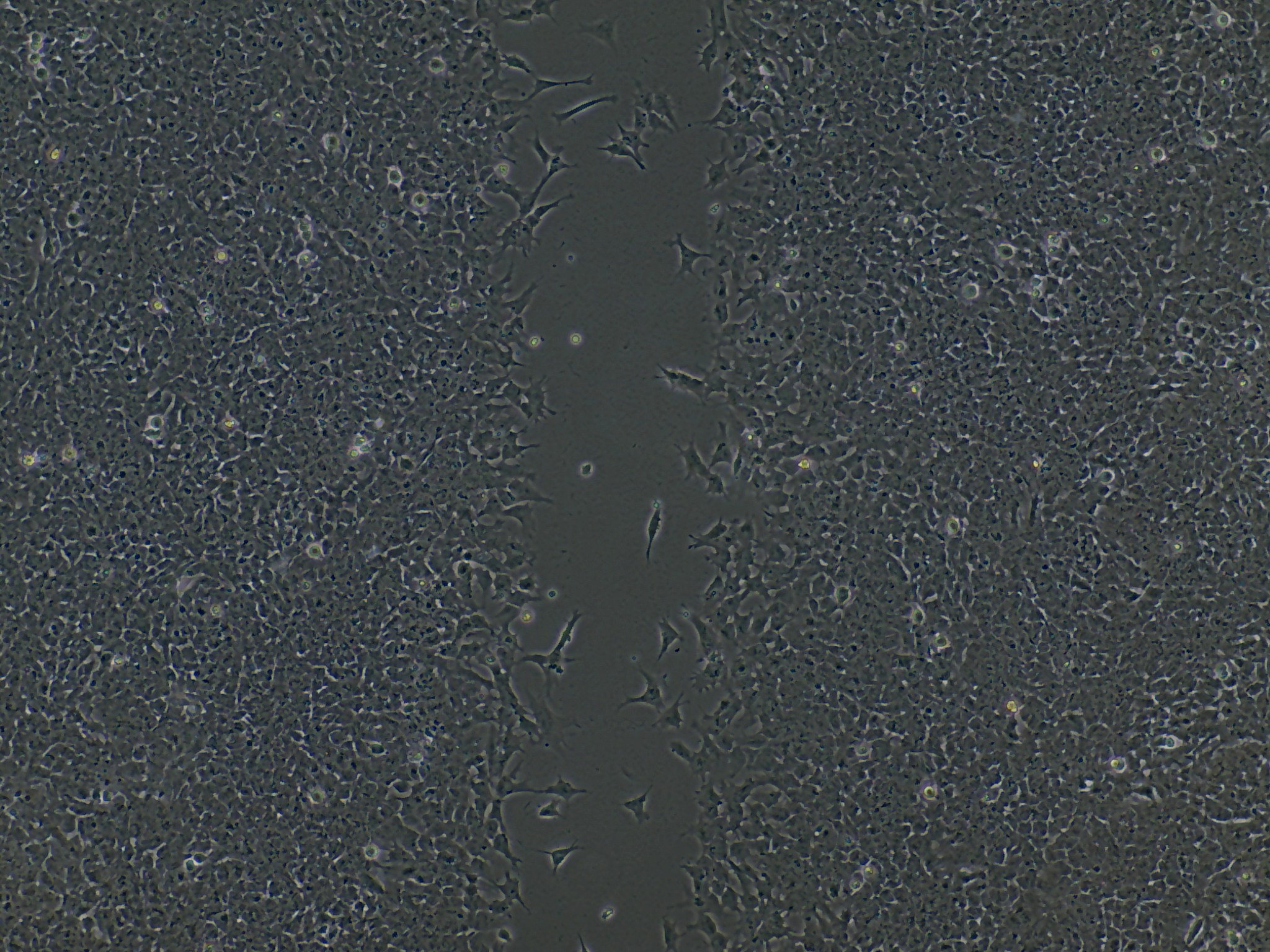

Supplement: Supplementary file 11 — Source data Fig. 4 [file 44320_2025_151_MOESM11_ESM.zip › FIGURE4/4D/250529-NCC-d5-SWA/NOG2-8h.jpg]

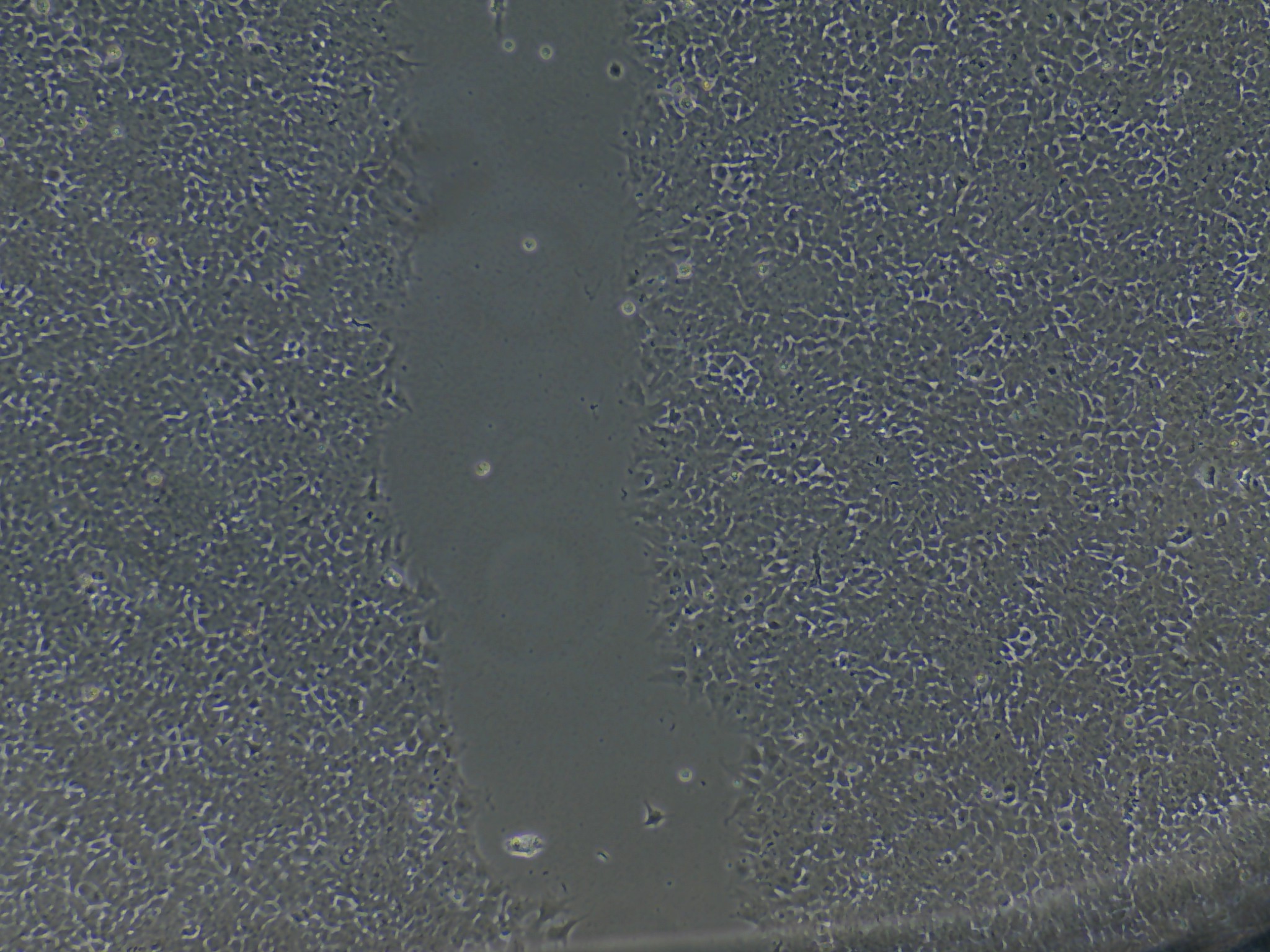

Supplement: Supplementary file 11 — Source data Fig. 4 [file 44320_2025_151_MOESM11_ESM.zip › FIGURE4/4D/250529-NCC-d5-SWA/HSTE3-8h.jpg]

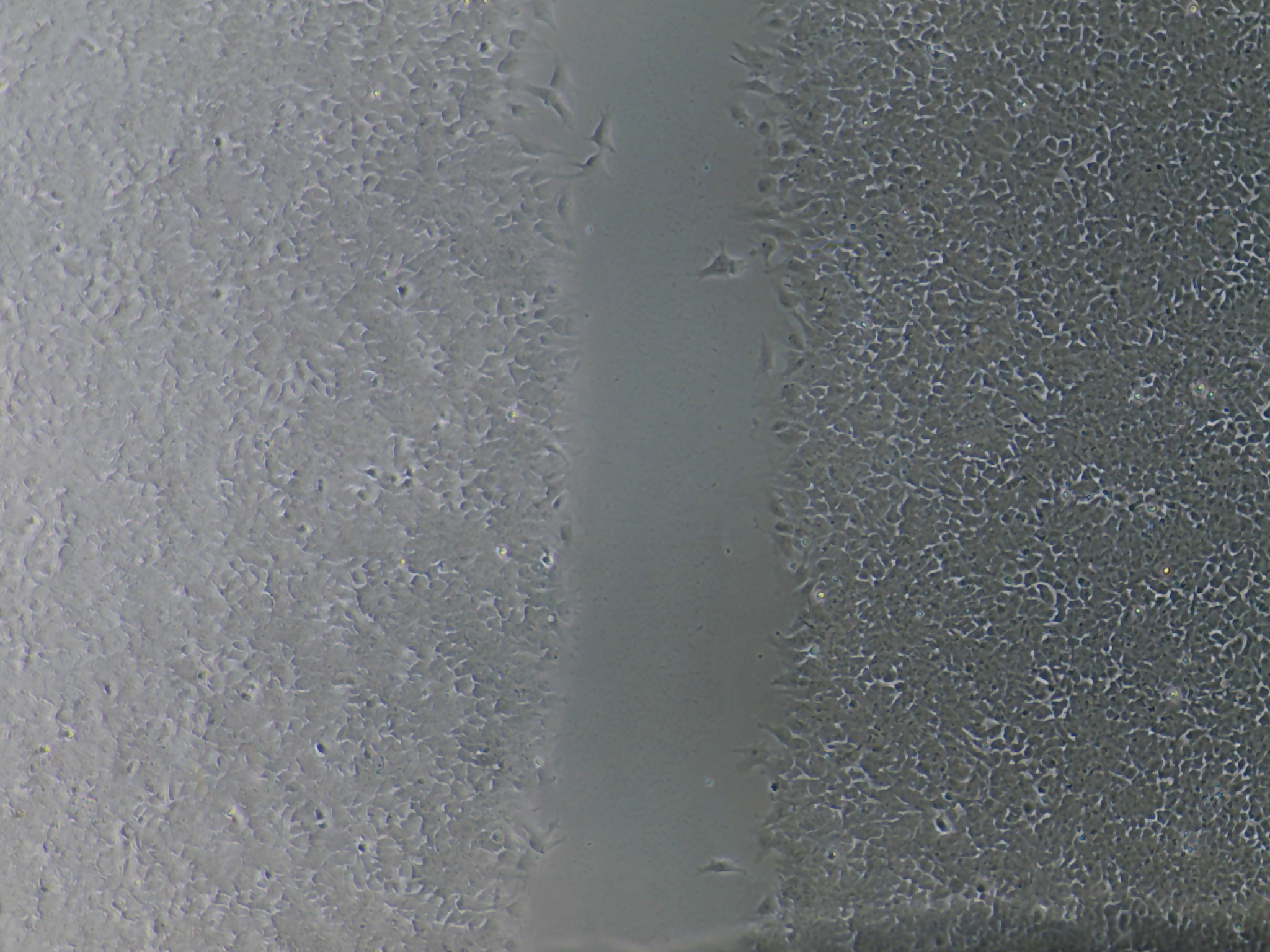

Supplement: Supplementary file 11 — Source data Fig. 4 [file 44320_2025_151_MOESM11_ESM.zip › FIGURE4/4D/250529-NCC-d5-SWA/HSTE6-8h.jpg]

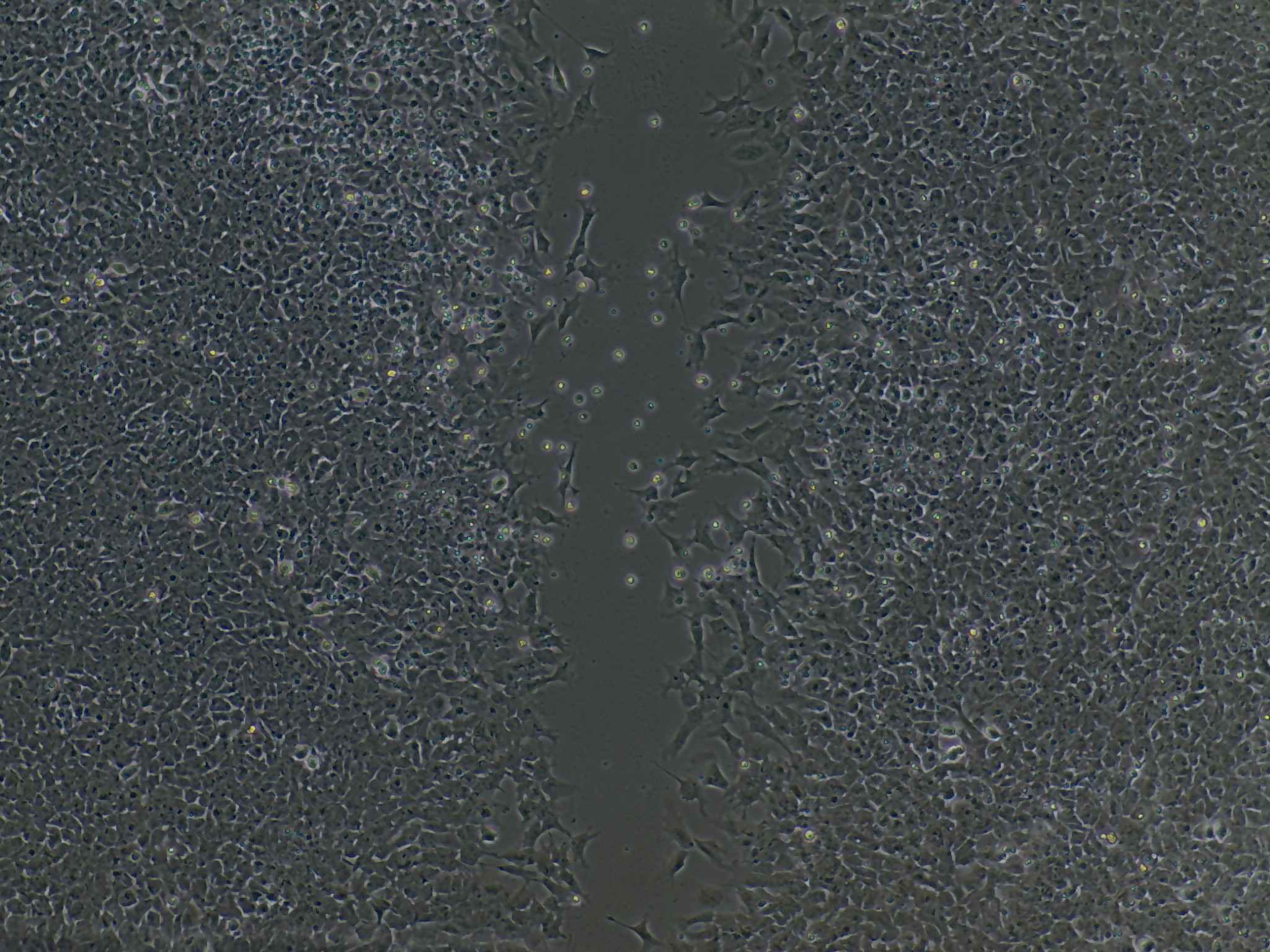

Supplement: Supplementary file 11 — Source data Fig. 4 [file 44320_2025_151_MOESM11_ESM.zip › FIGURE4/4D/250529-NCC-d5-SWA/NOG7-8h.jpg]

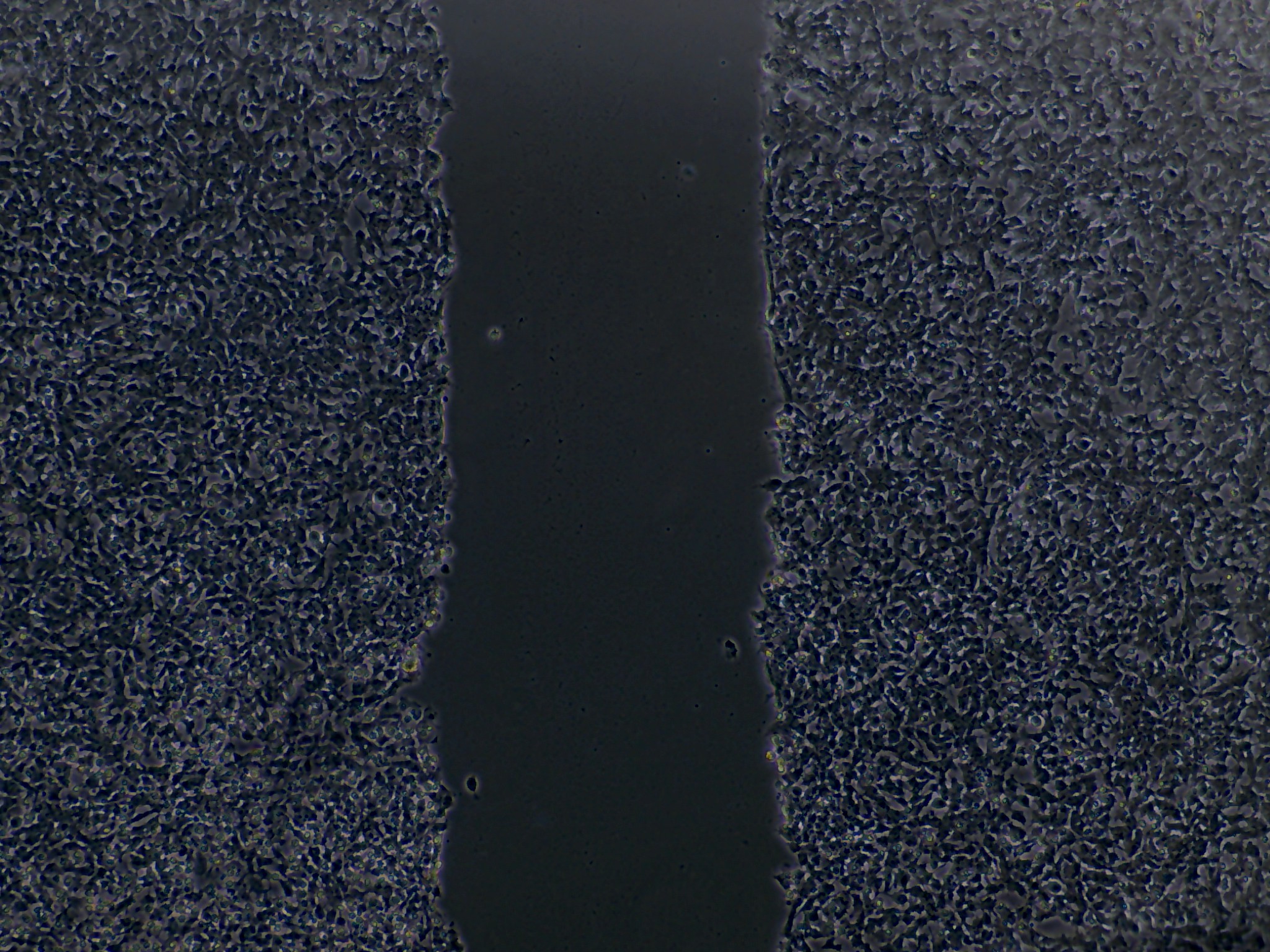

Supplement: Supplementary file 11 — Source data Fig. 4 [file 44320_2025_151_MOESM11_ESM.zip › FIGURE4/4D/250529-NCC-d5-SWA/NOG12-0h.jpg]

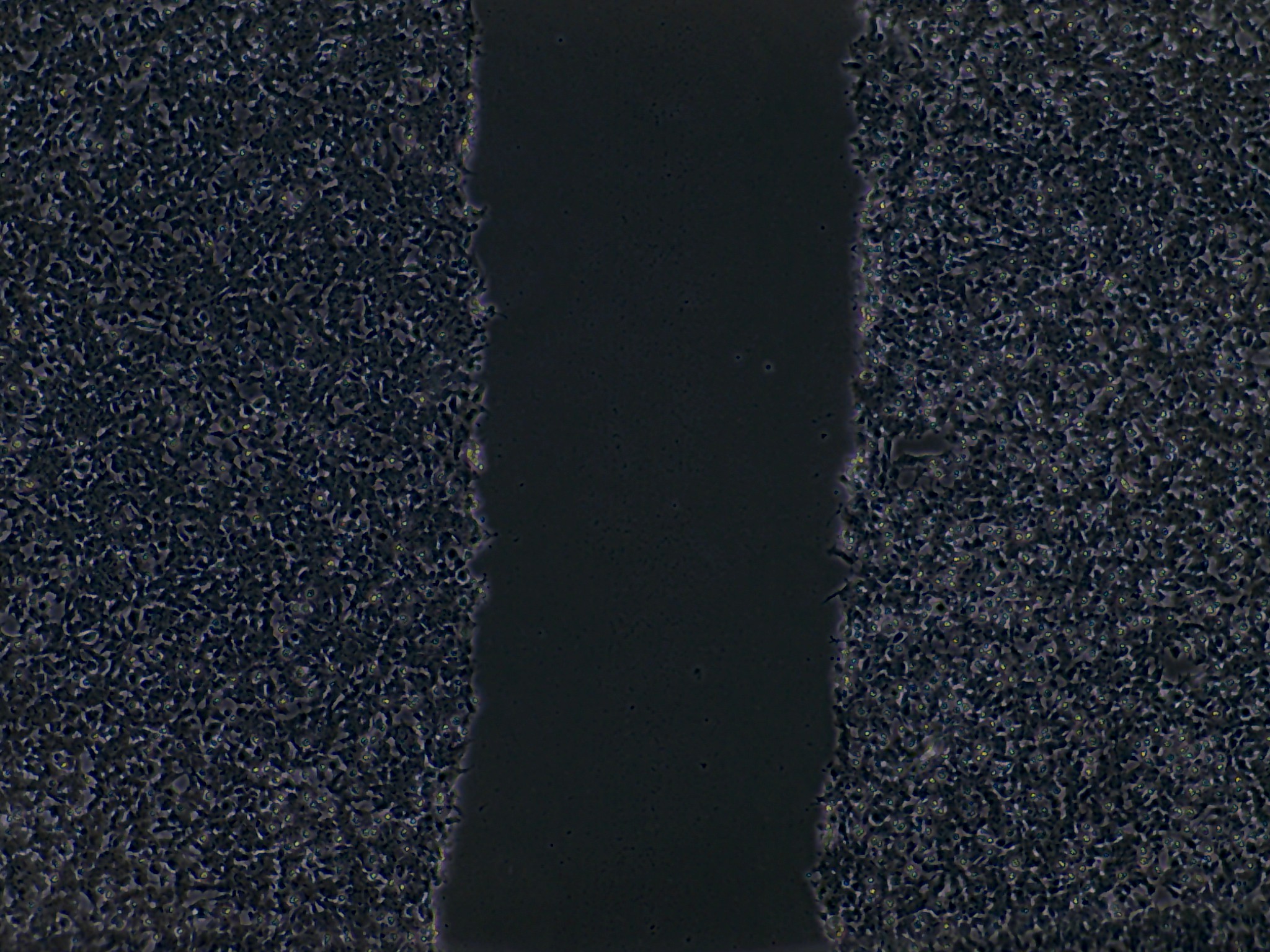

Supplement: Supplementary file 11 — Source data Fig. 4 [file 44320_2025_151_MOESM11_ESM.zip › FIGURE4/4D/250529-NCC-d5-SWA/HSTE7-0h.jpg]

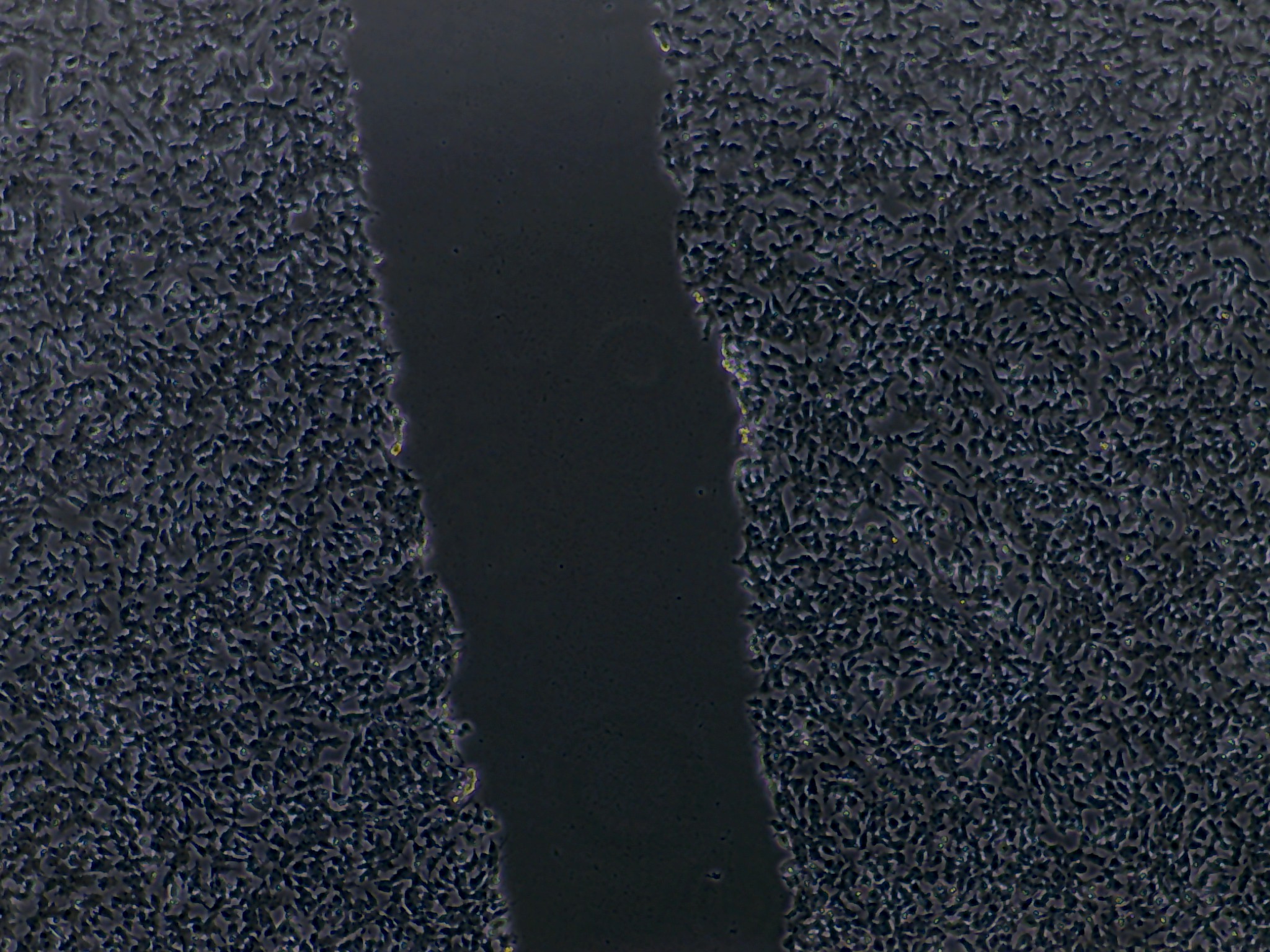

Supplement: Supplementary file 11 — Source data Fig. 4 [file 44320_2025_151_MOESM11_ESM.zip › FIGURE4/4D/250529-NCC-d5-SWA/NOG6-0h.jpg]

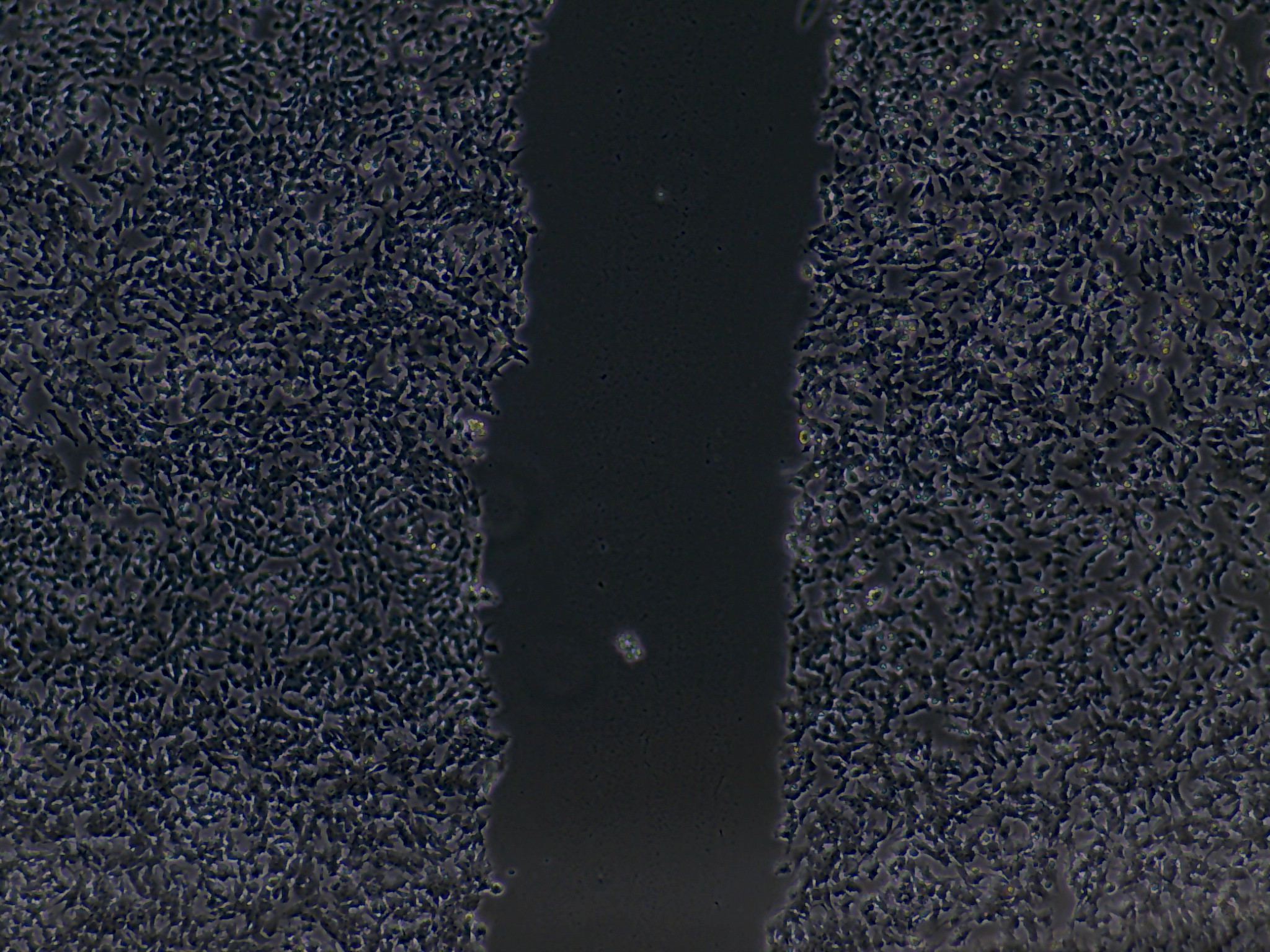

Supplement: Supplementary file 11 — Source data Fig. 4 [file 44320_2025_151_MOESM11_ESM.zip › FIGURE4/4D/250529-NCC-d5-SWA/NOG4-0h.jpg]

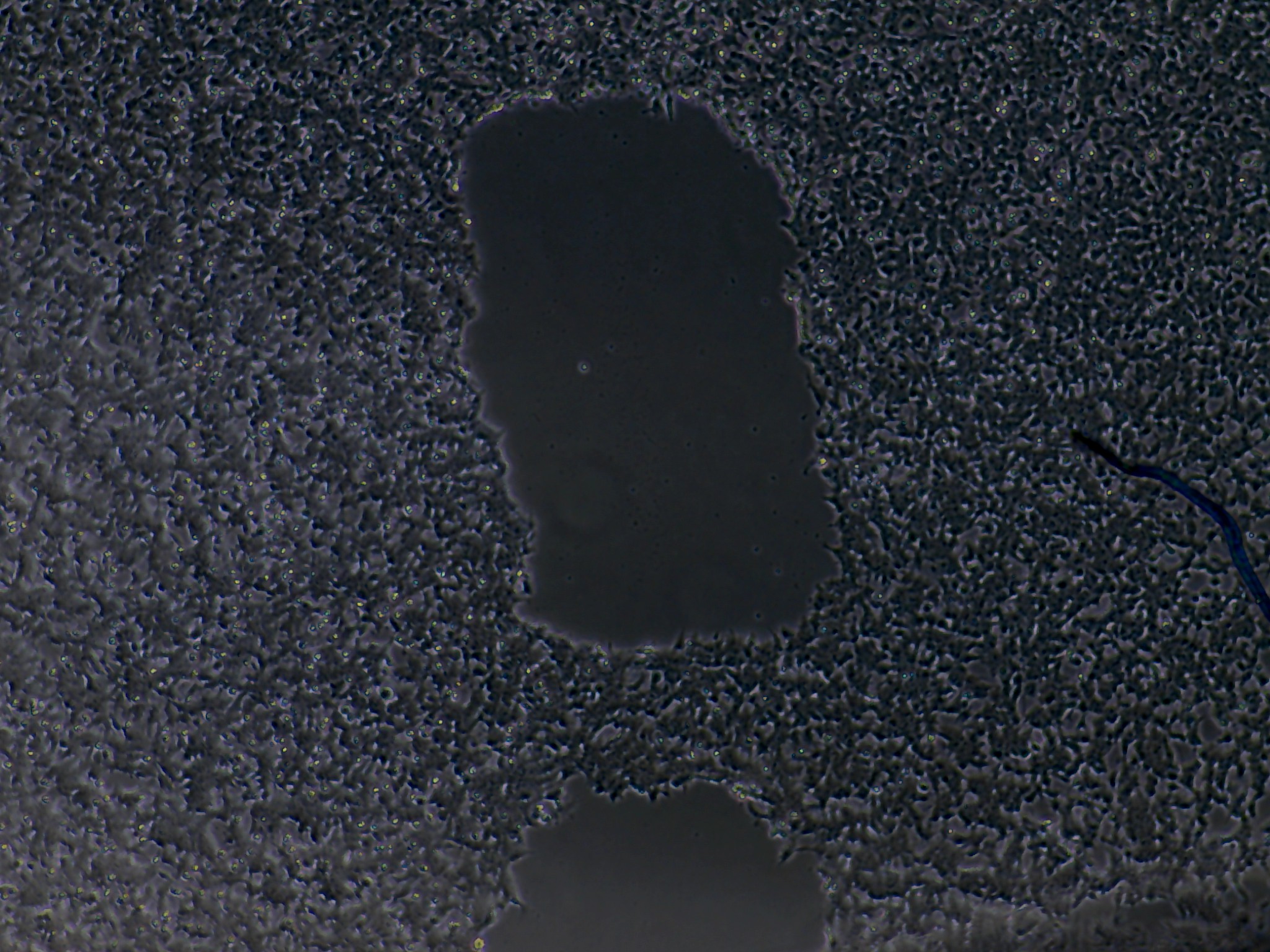

Supplement: Supplementary file 11 — Source data Fig. 4 [file 44320_2025_151_MOESM11_ESM.zip › FIGURE4/4D/250529-NCC-d5-SWA/HSTE5-0h.jpg]

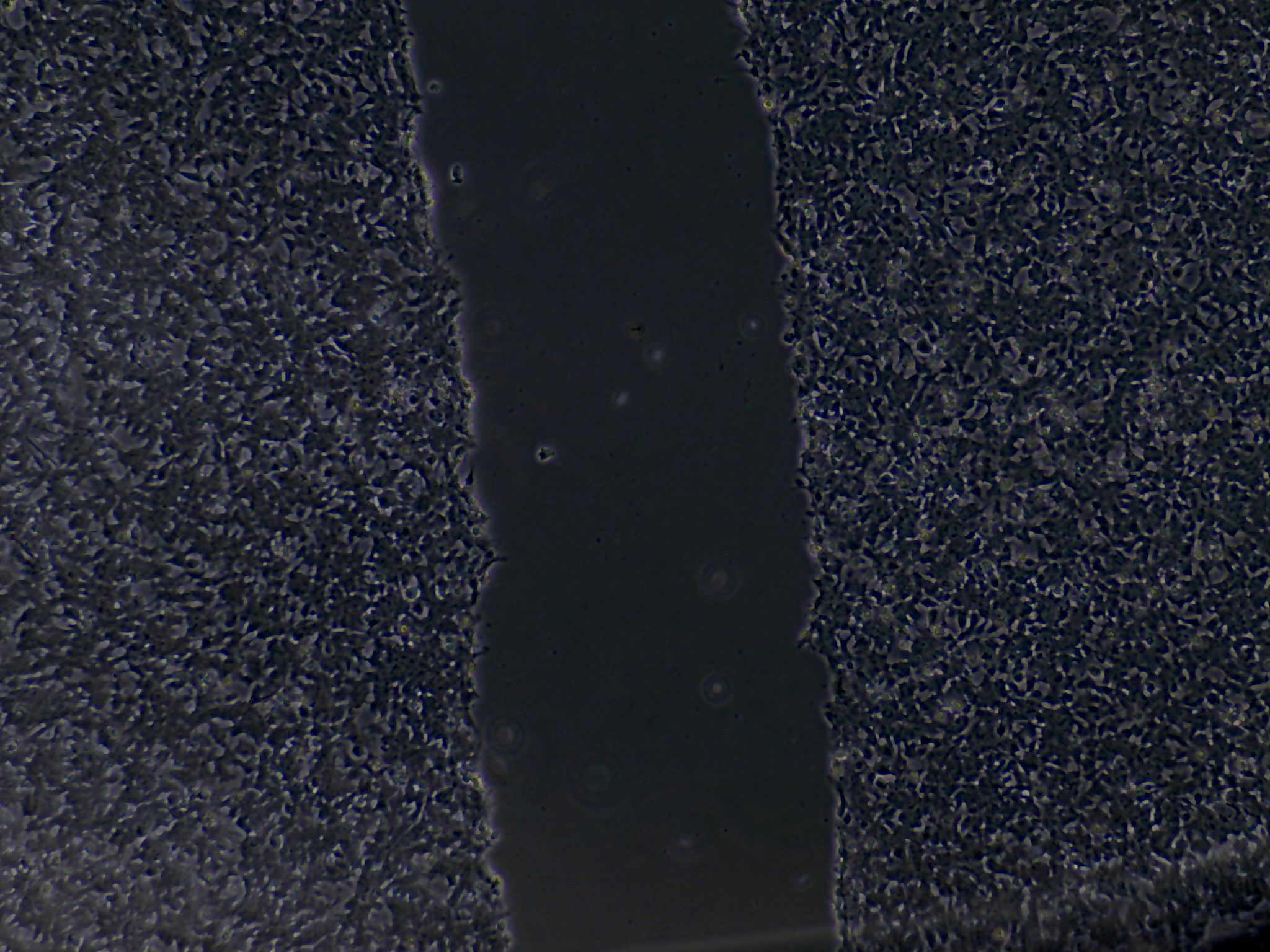

Supplement: Supplementary file 11 — Source data Fig. 4 [file 44320_2025_151_MOESM11_ESM.zip › FIGURE4/4D/250529-NCC-d5-SWA/HSTE9-0h.jpg]

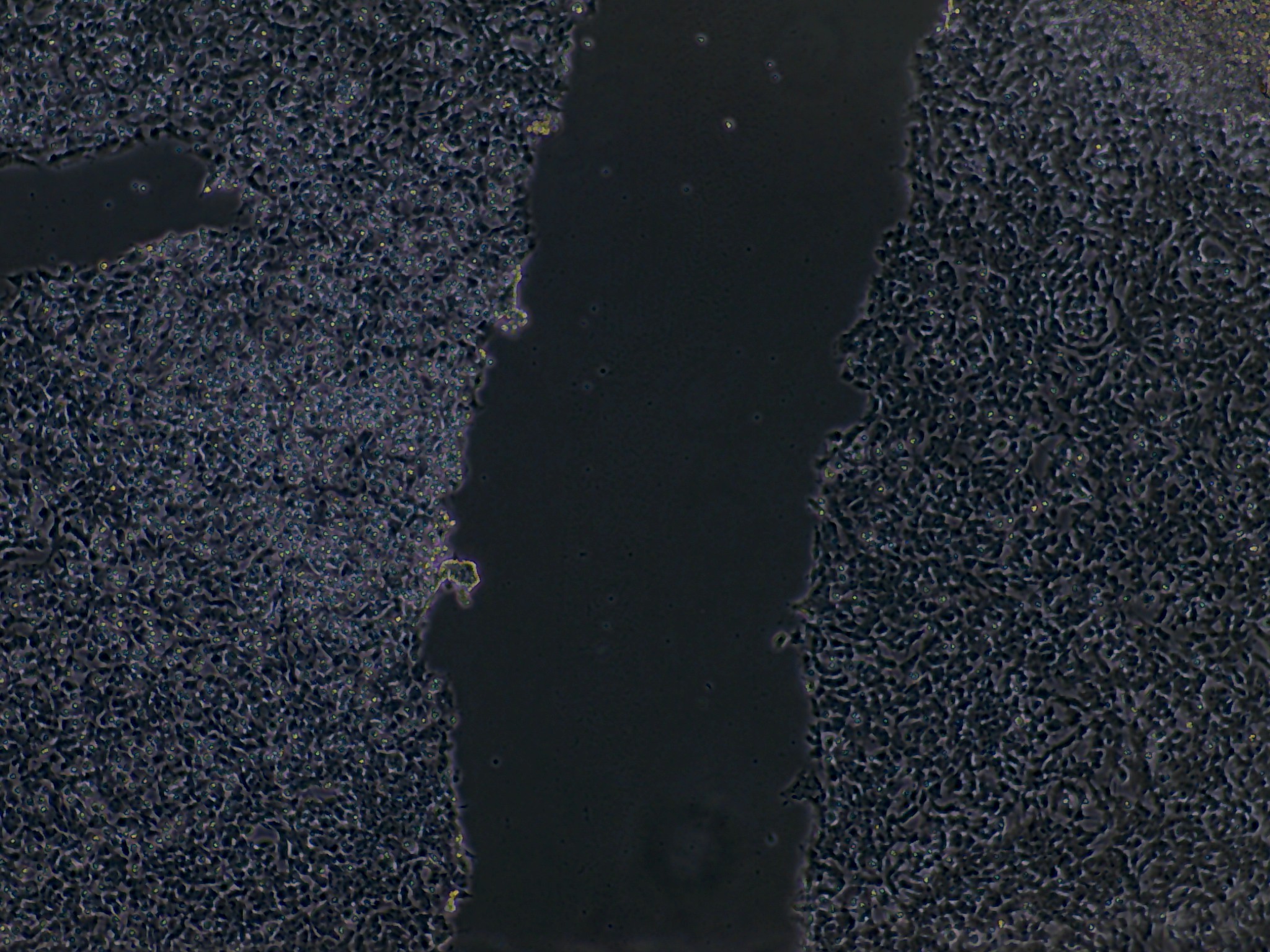

Supplement: Supplementary file 11 — Source data Fig. 4 [file 44320_2025_151_MOESM11_ESM.zip › FIGURE4/4D/250529-NCC-d5-SWA/NOG8-0h.jpg]

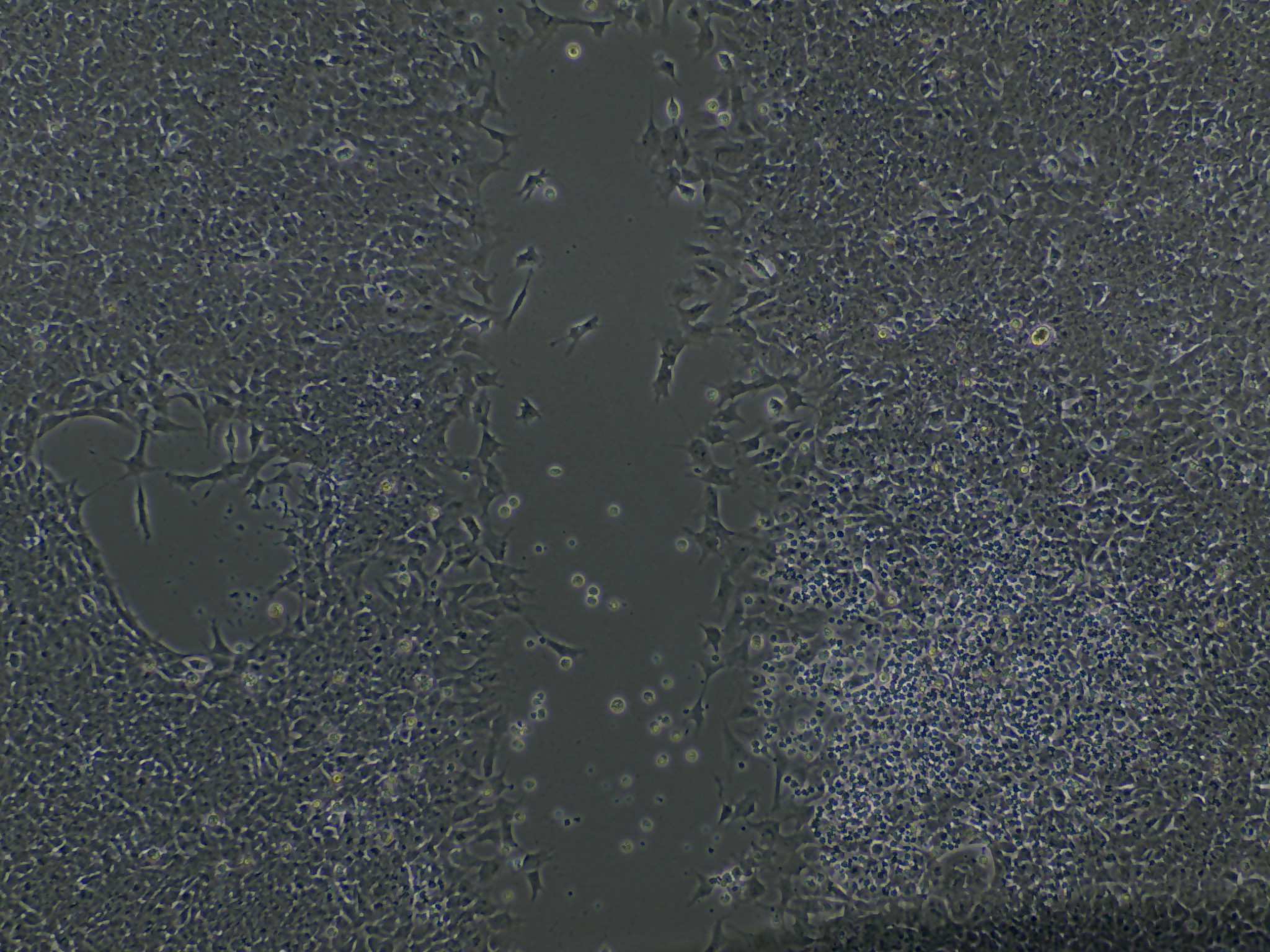

Supplement: Supplementary file 11 — Source data Fig. 4 [file 44320_2025_151_MOESM11_ESM.zip › FIGURE4/4D/250529-NCC-d5-SWA/NOG11-8h.jpg]

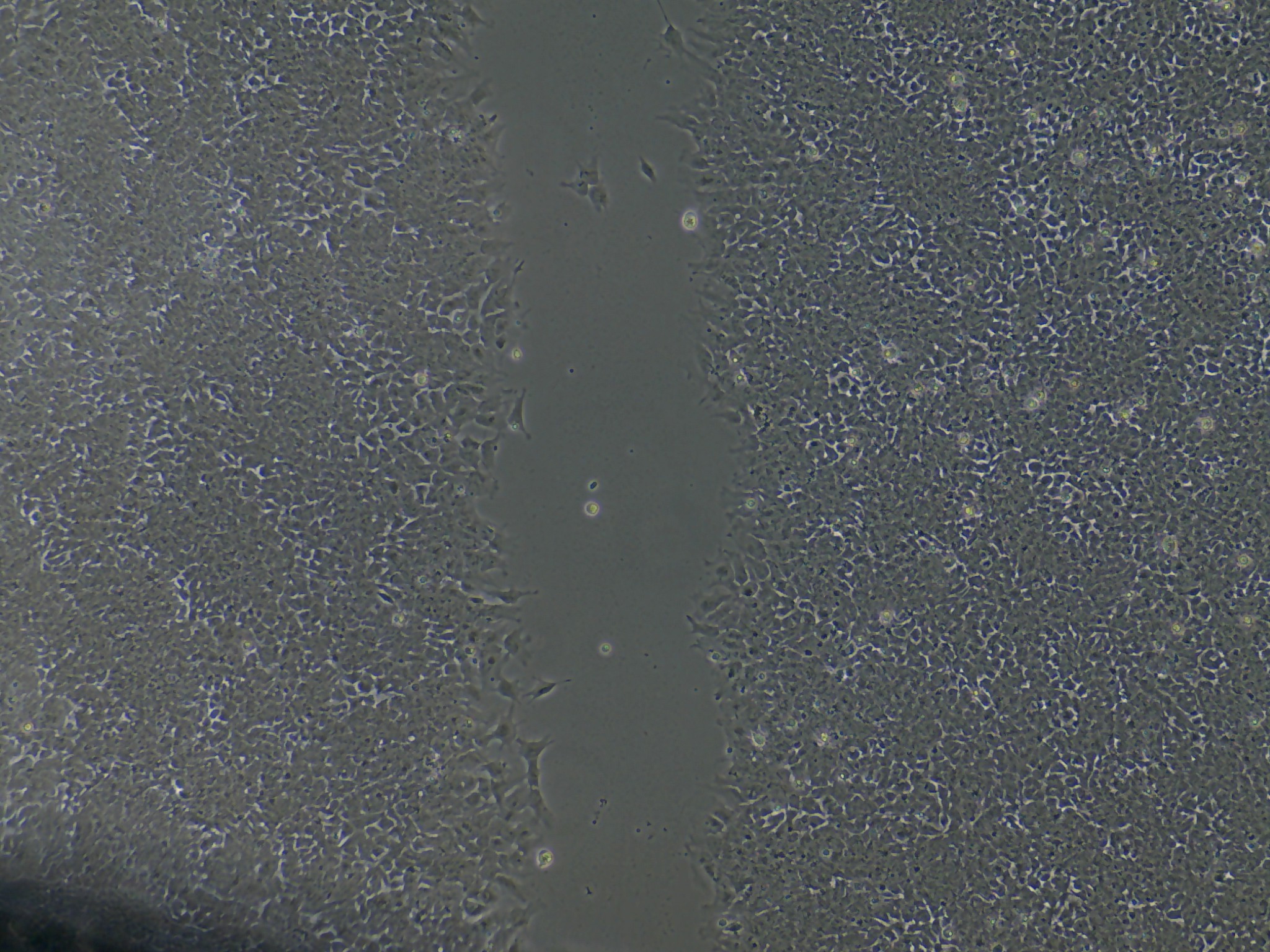

Supplement: Supplementary file 11 — Source data Fig. 4 [file 44320_2025_151_MOESM11_ESM.zip › FIGURE4/4D/250529-NCC-d5-SWA/HSTE8-8h.jpg]

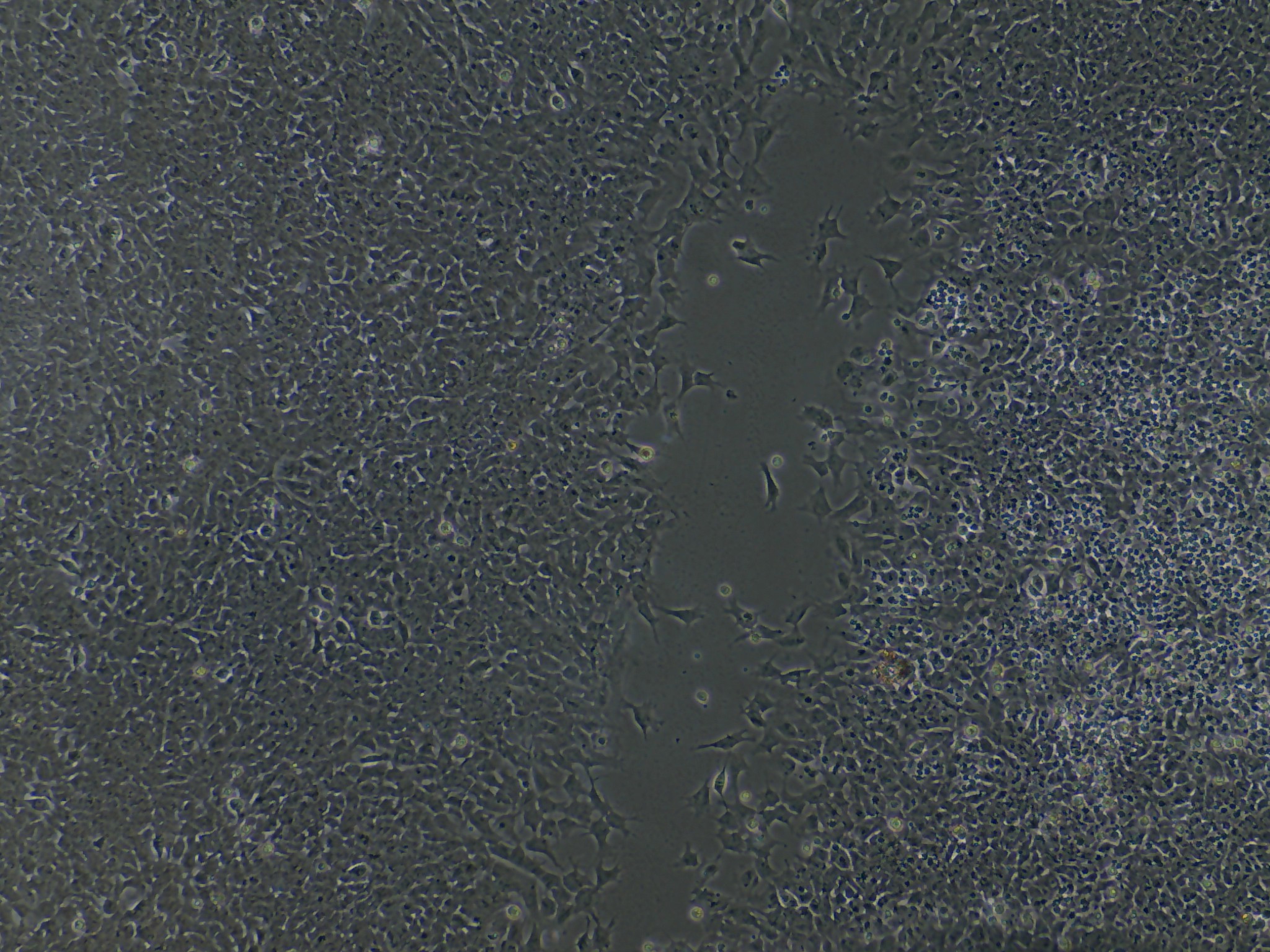

Supplement: Supplementary file 11 — Source data Fig. 4 [file 44320_2025_151_MOESM11_ESM.zip › FIGURE4/4D/250529-NCC-d5-SWA/NOG9-8h.jpg]

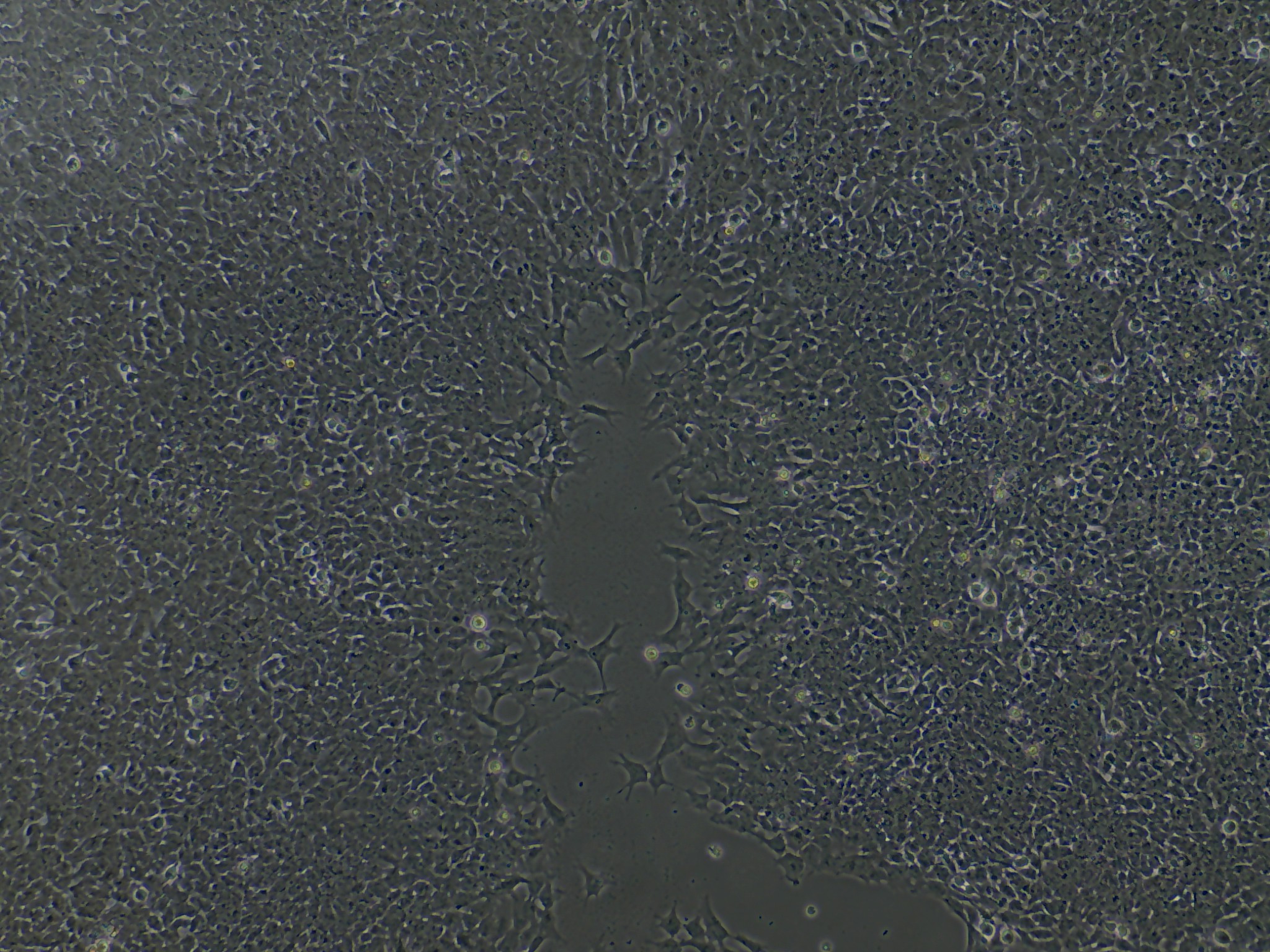

Supplement: Supplementary file 11 — Source data Fig. 4 [file 44320_2025_151_MOESM11_ESM.zip › FIGURE4/4D/250529-NCC-d5-SWA/NOG5-8h.jpg]

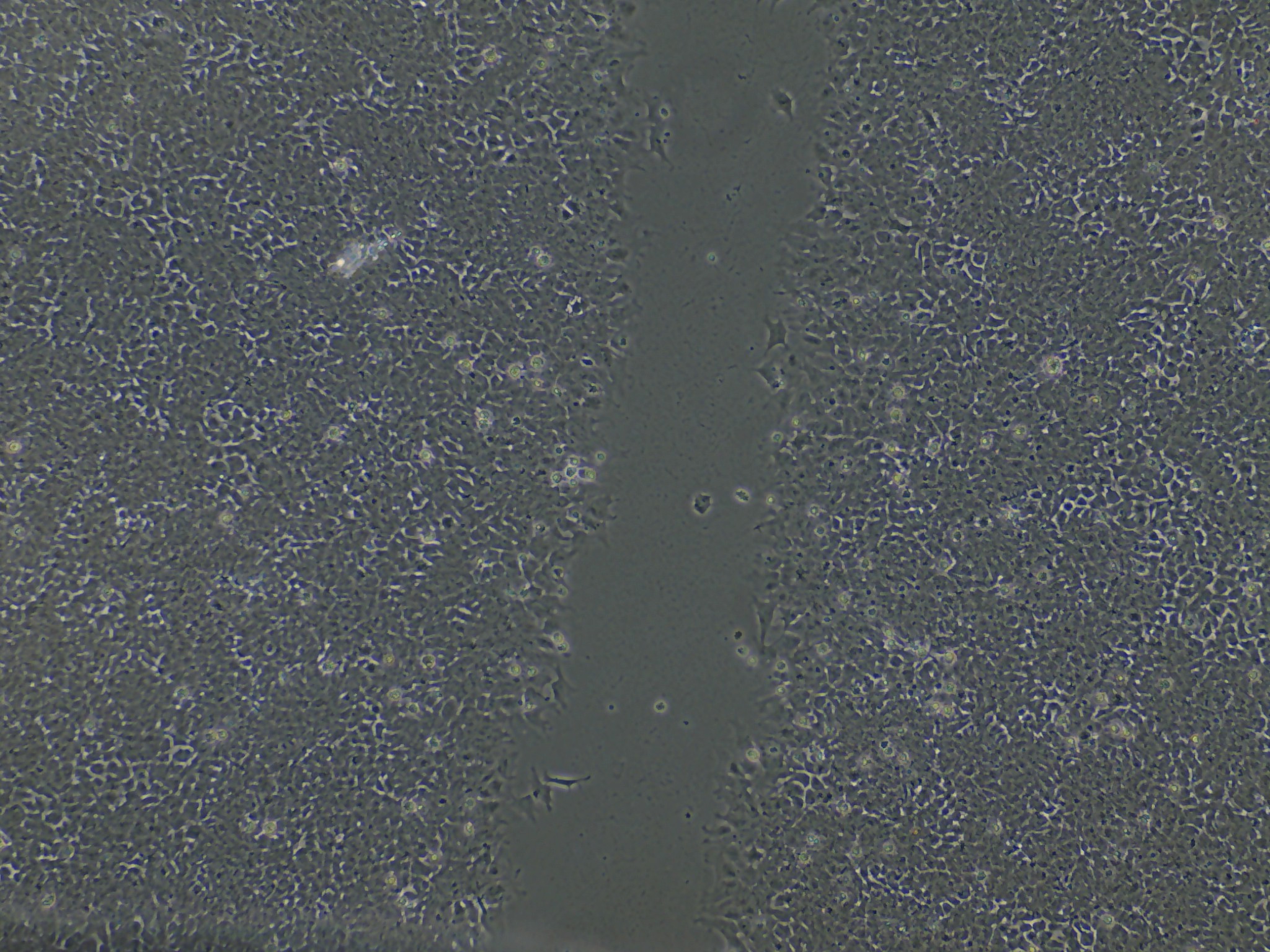

Supplement: Supplementary file 11 — Source data Fig. 4 [file 44320_2025_151_MOESM11_ESM.zip › FIGURE4/4D/250529-NCC-d5-SWA/HSTE4-8h.jpg]

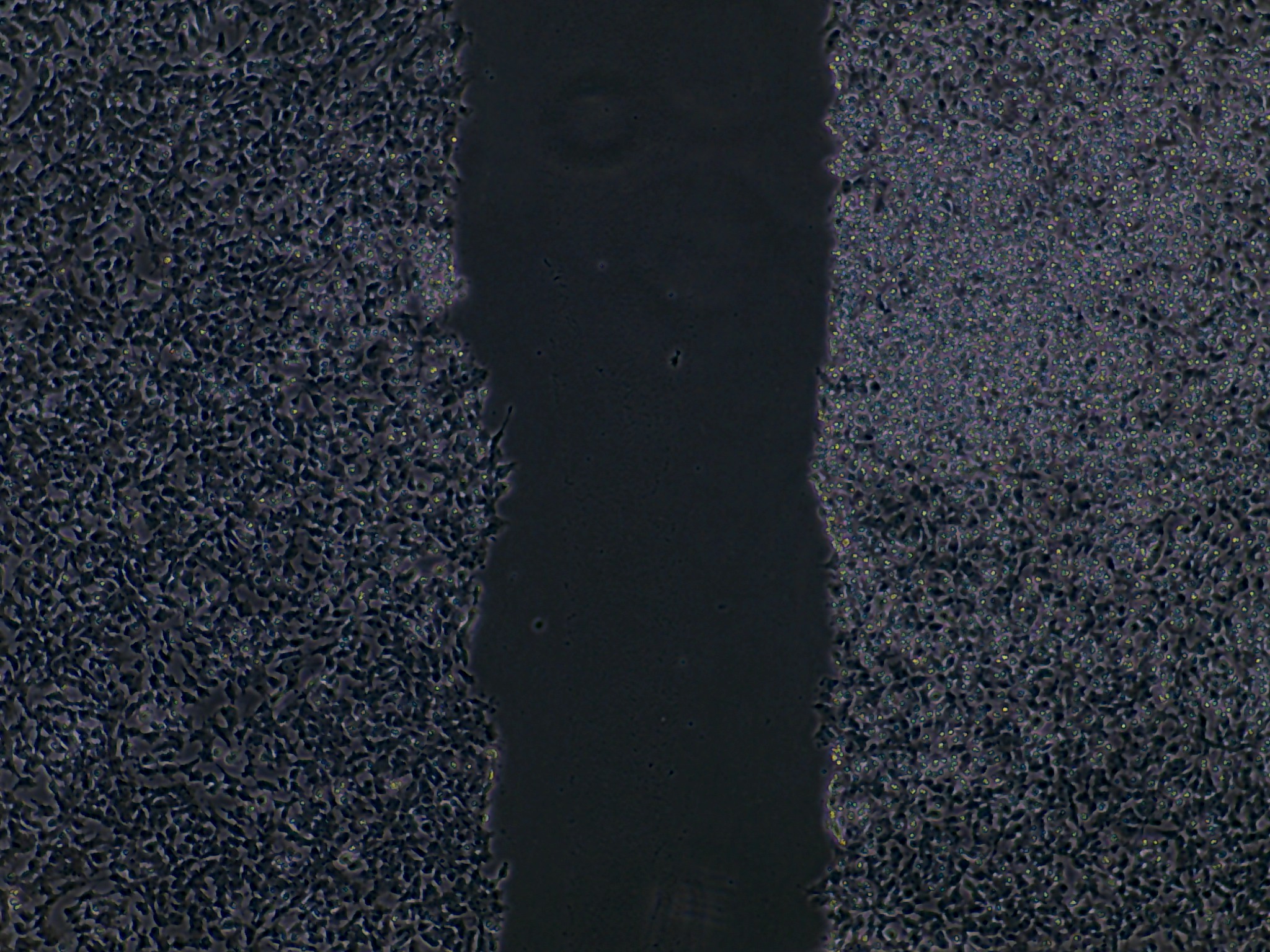

Supplement: Supplementary file 11 — Source data Fig. 4 [file 44320_2025_151_MOESM11_ESM.zip › FIGURE4/4D/250529-NCC-d5-SWA/NOG10-0h.jpg]

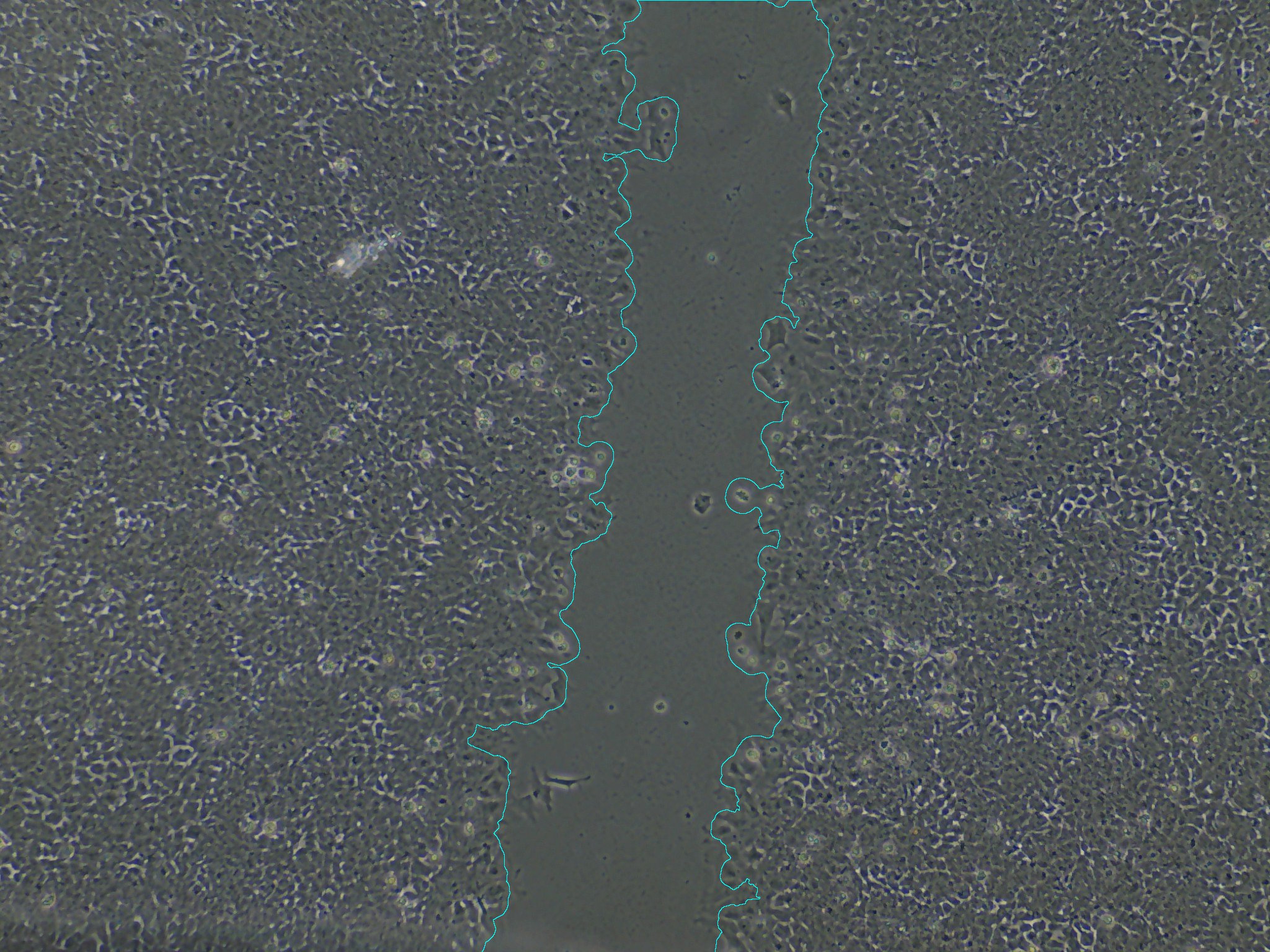

Supplement: Supplementary file 11 — Source data Fig. 4 [file 44320_2025_151_MOESM11_ESM.zip › FIGURE4/4D/250529-NCC-d5-SWA/HSTE4-8h-1-flatten.jpg]

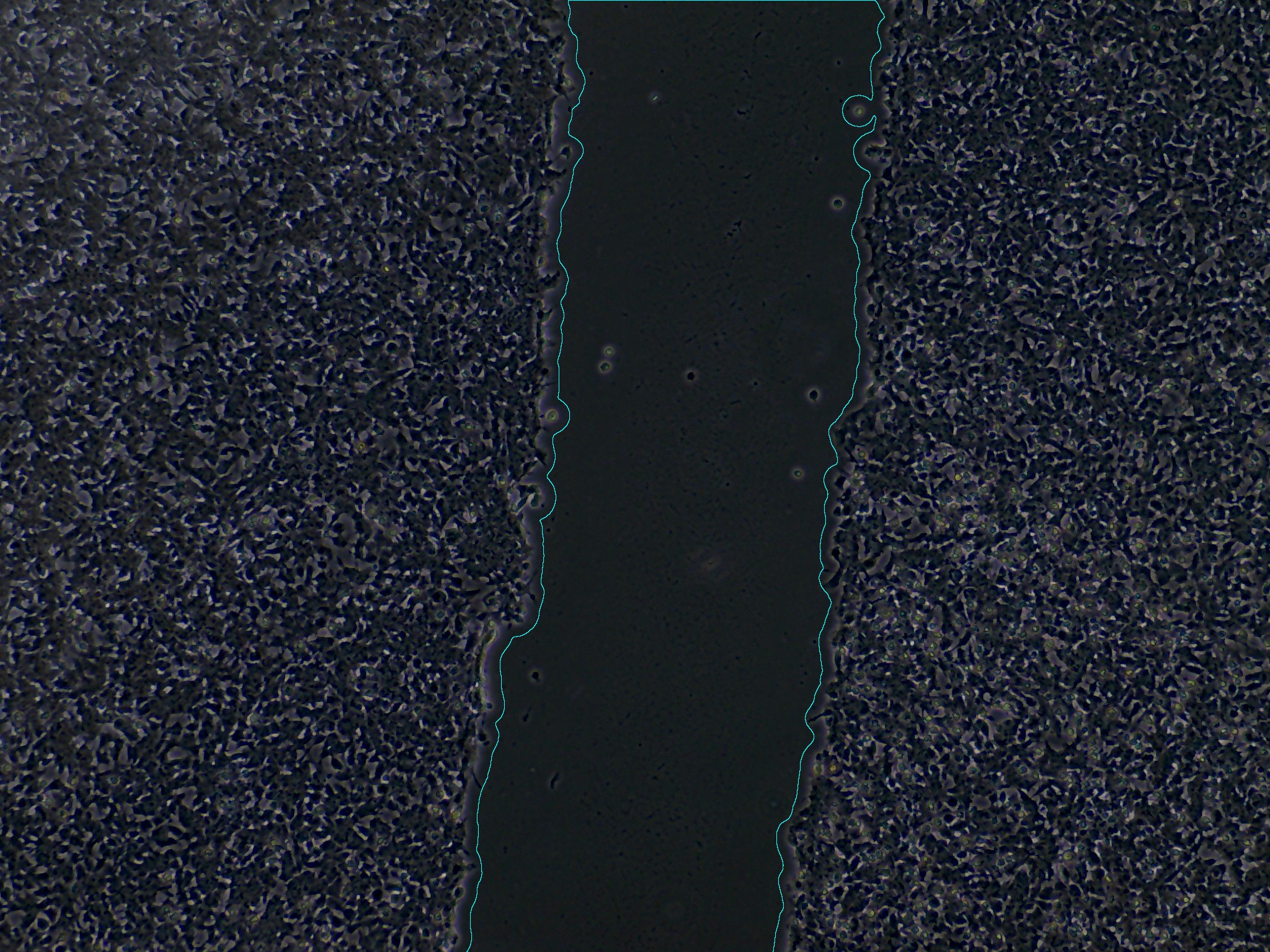

Supplement: Supplementary file 11 — Source data Fig. 4 [file 44320_2025_151_MOESM11_ESM.zip › FIGURE4/4D/250529-NCC-d5-SWA/HSTE4-0h-1-flatten.jpg]

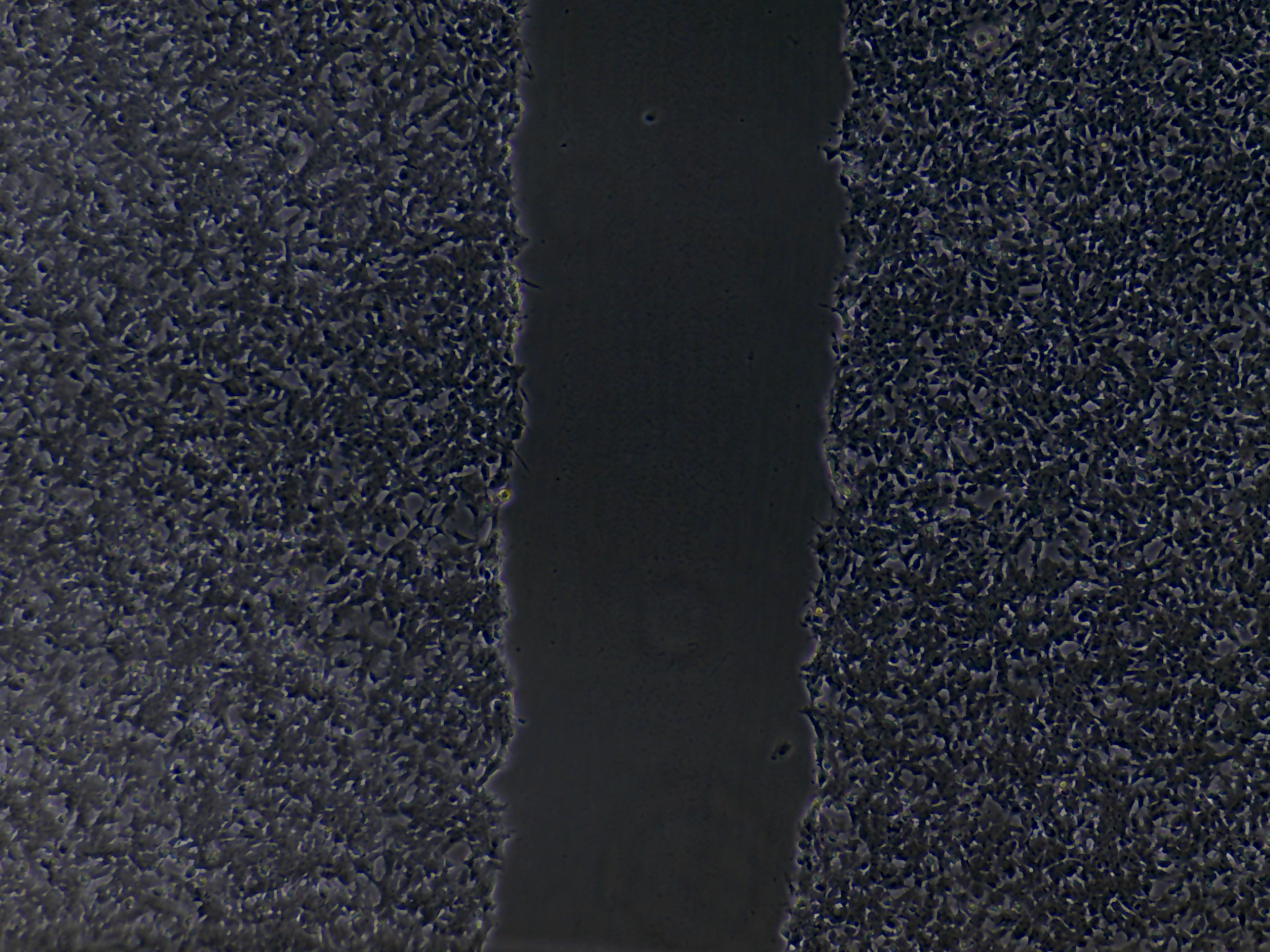

Supplement: Supplementary file 11 — Source data Fig. 4 [file 44320_2025_151_MOESM11_ESM.zip › FIGURE4/4D/250529-NCC-d5-SWA/HSTE1-0h.jpg]

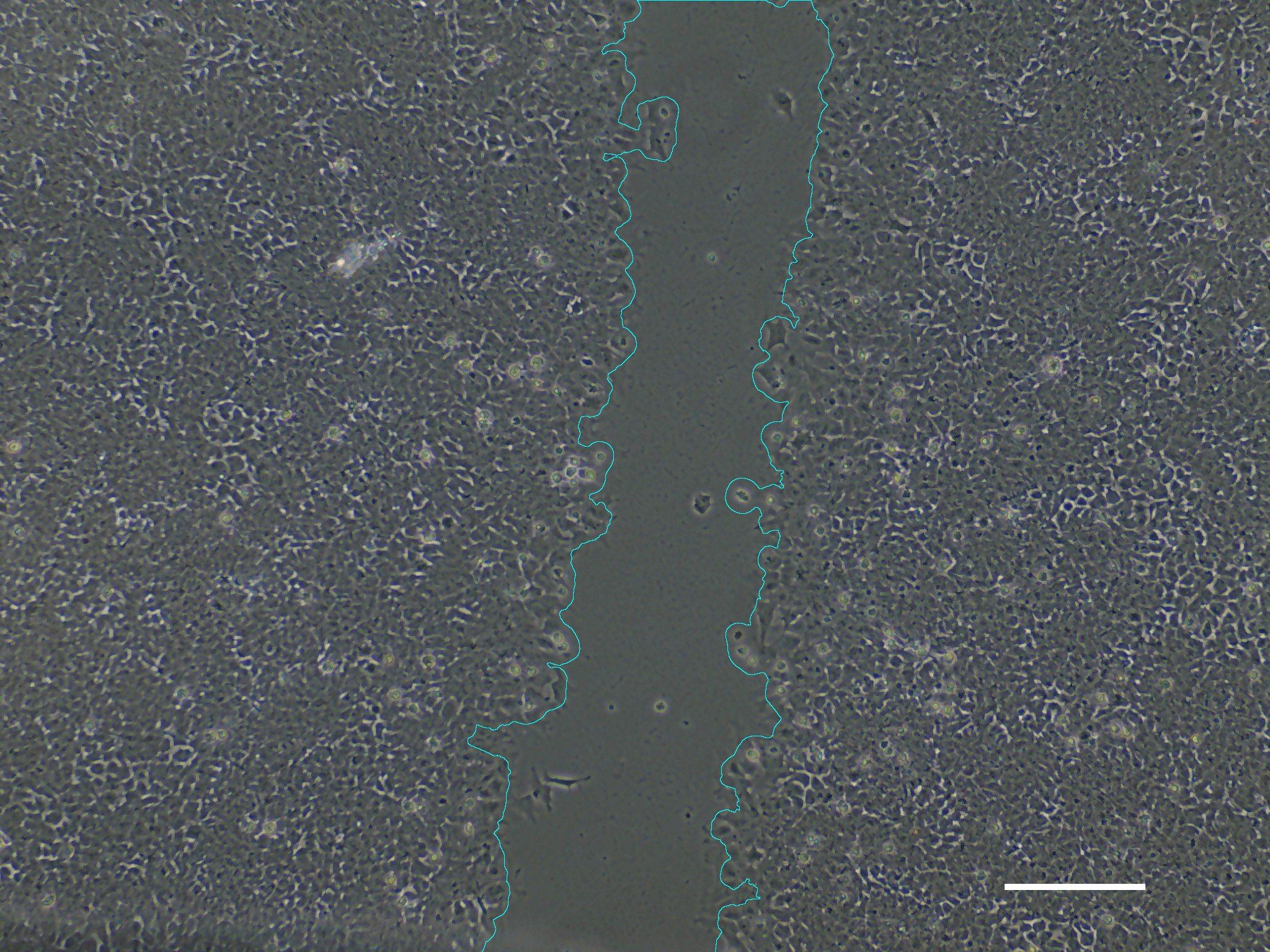

Supplement: Supplementary file 11 — Source data Fig. 4 [file 44320_2025_151_MOESM11_ESM.zip › FIGURE4/4D/250529-NCC-d5-SWA/HSTE4-8h-1-flatten-scalebar.jpg]

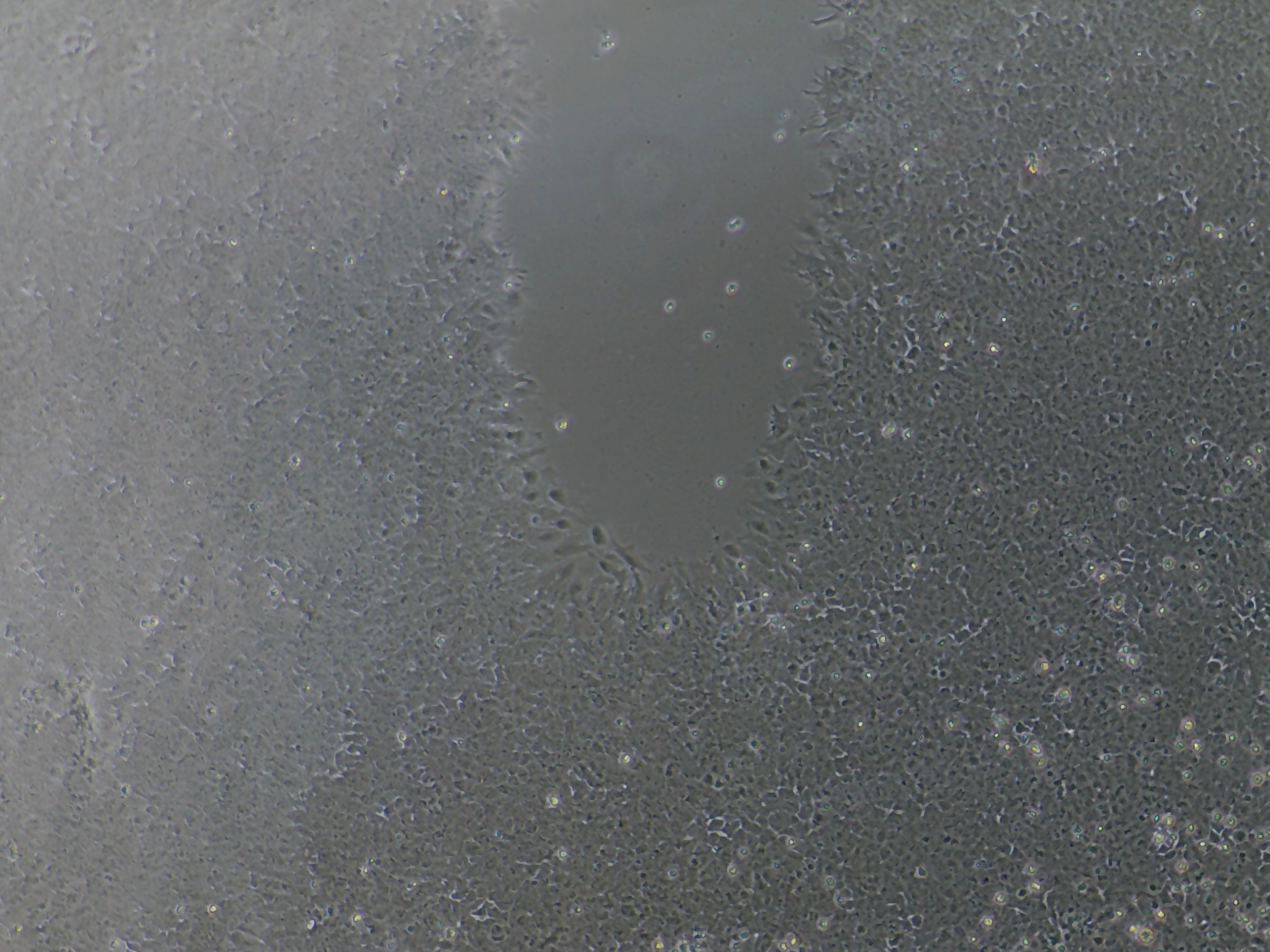

Supplement: Supplementary file 11 — Source data Fig. 4 [file 44320_2025_151_MOESM11_ESM.zip › FIGURE4/4D/250529-NCC-d5-SWA/HSTE12-8h.jpg]

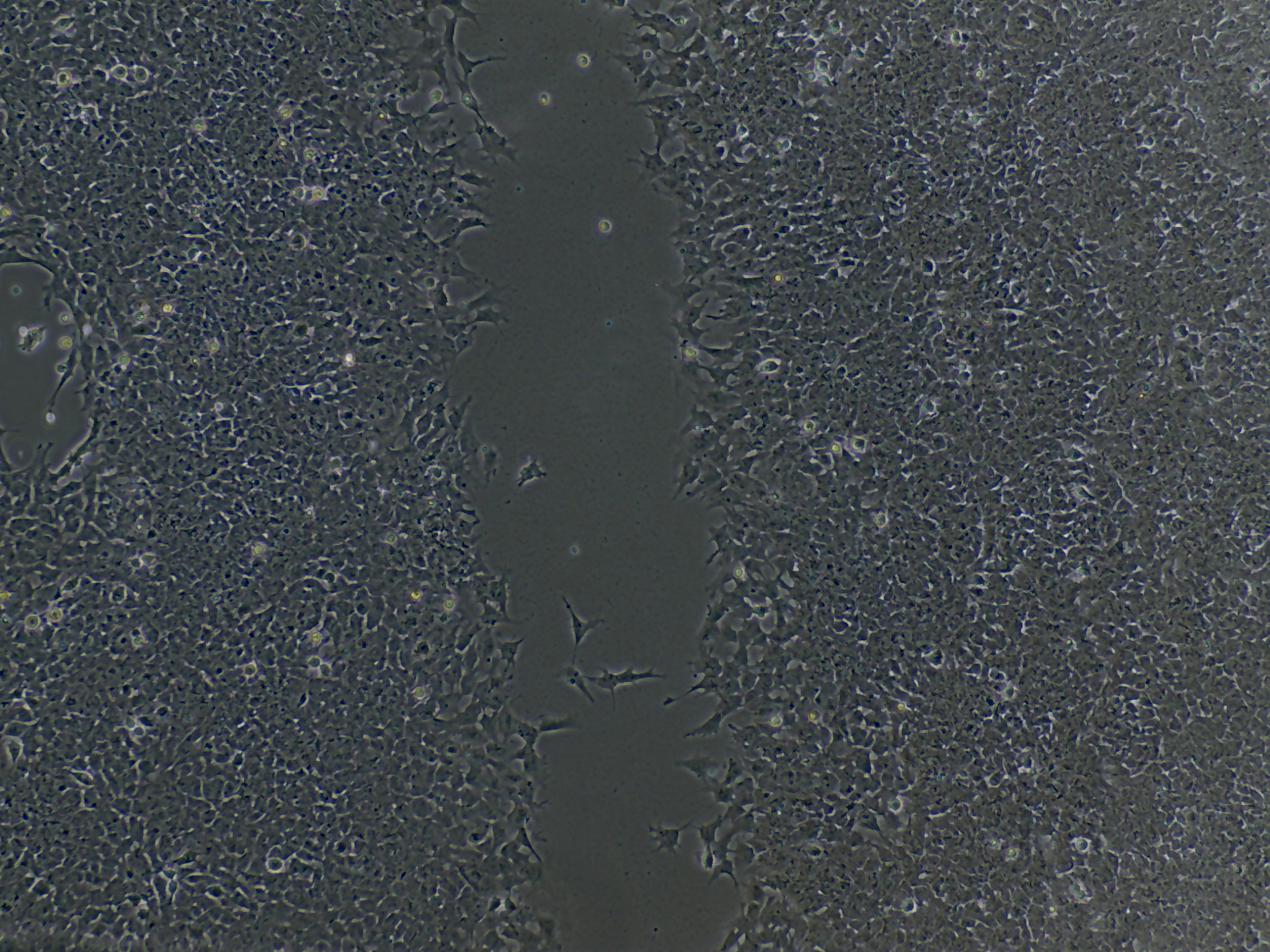

Supplement: Supplementary file 11 — Source data Fig. 4 [file 44320_2025_151_MOESM11_ESM.zip › FIGURE4/4D/250529-NCC-d5-SWA/NOG1-8h.jpg]

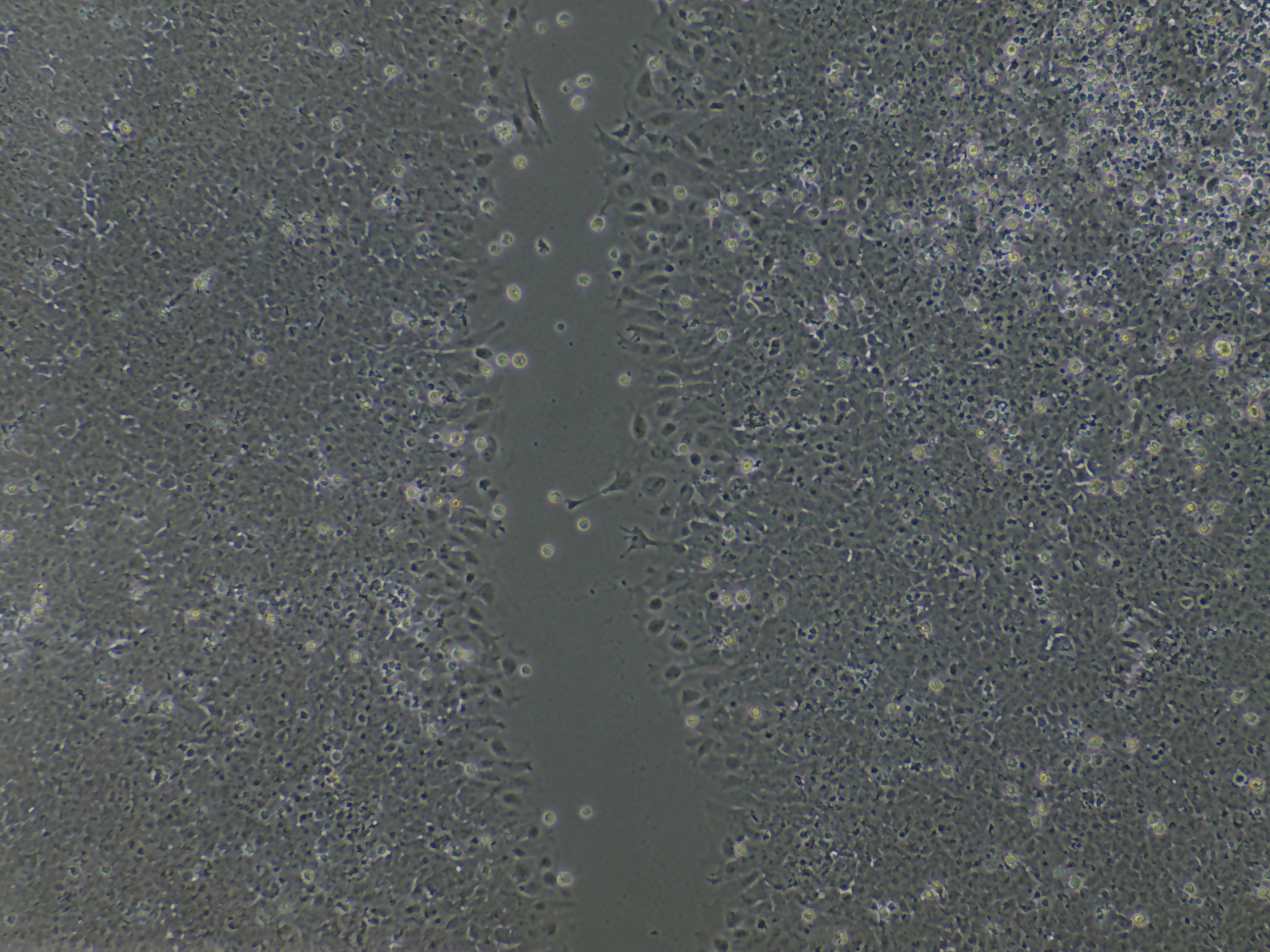

Supplement: Supplementary file 11 — Source data Fig. 4 [file 44320_2025_151_MOESM11_ESM.zip › FIGURE4/4D/250529-NCC-d5-SWA/HSTE10-8h.jpg]

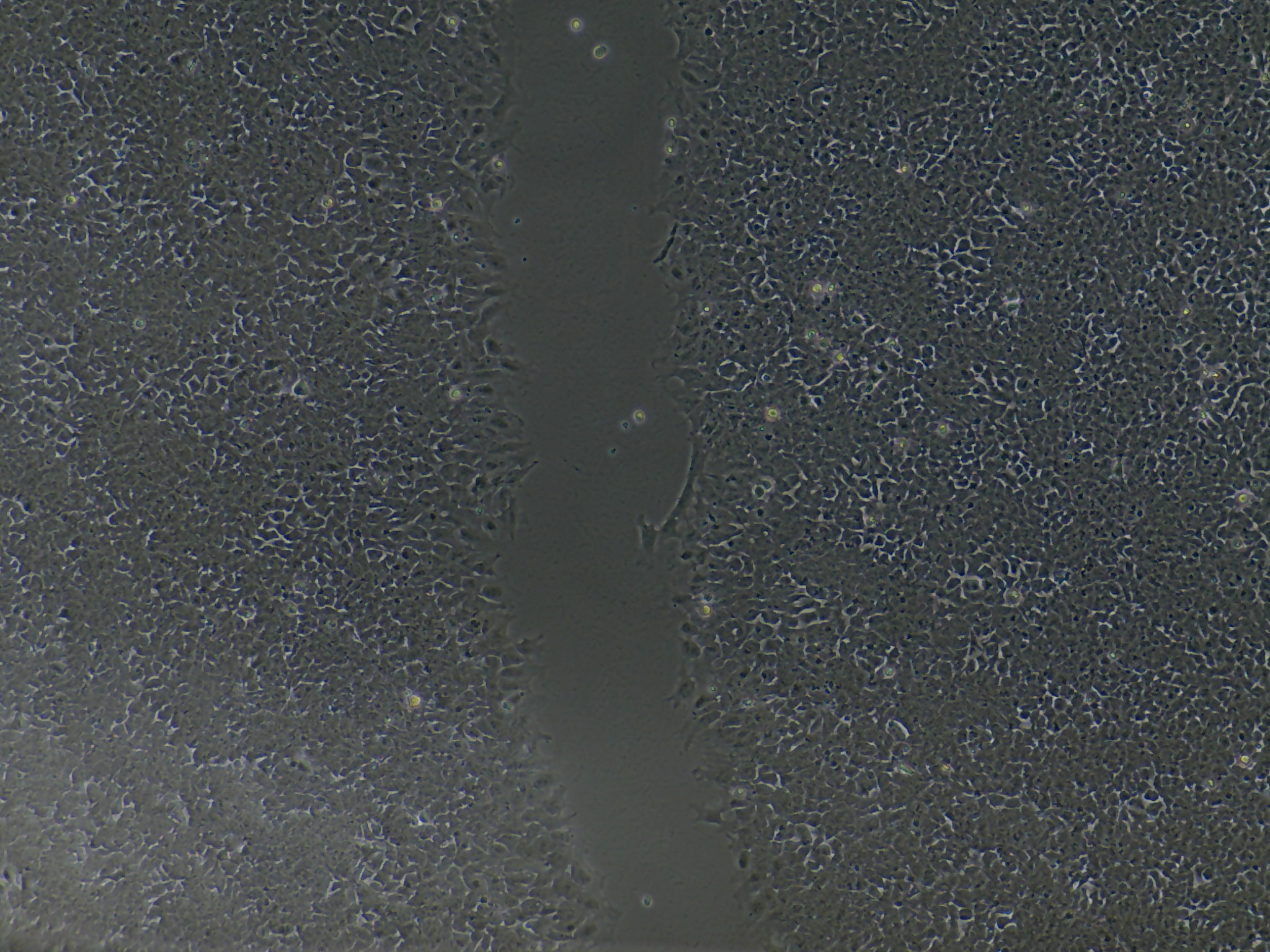

Supplement: Supplementary file 11 — Source data Fig. 4 [file 44320_2025_151_MOESM11_ESM.zip › FIGURE4/4D/250529-NCC-d5-SWA/HSTE2-8h.jpg]

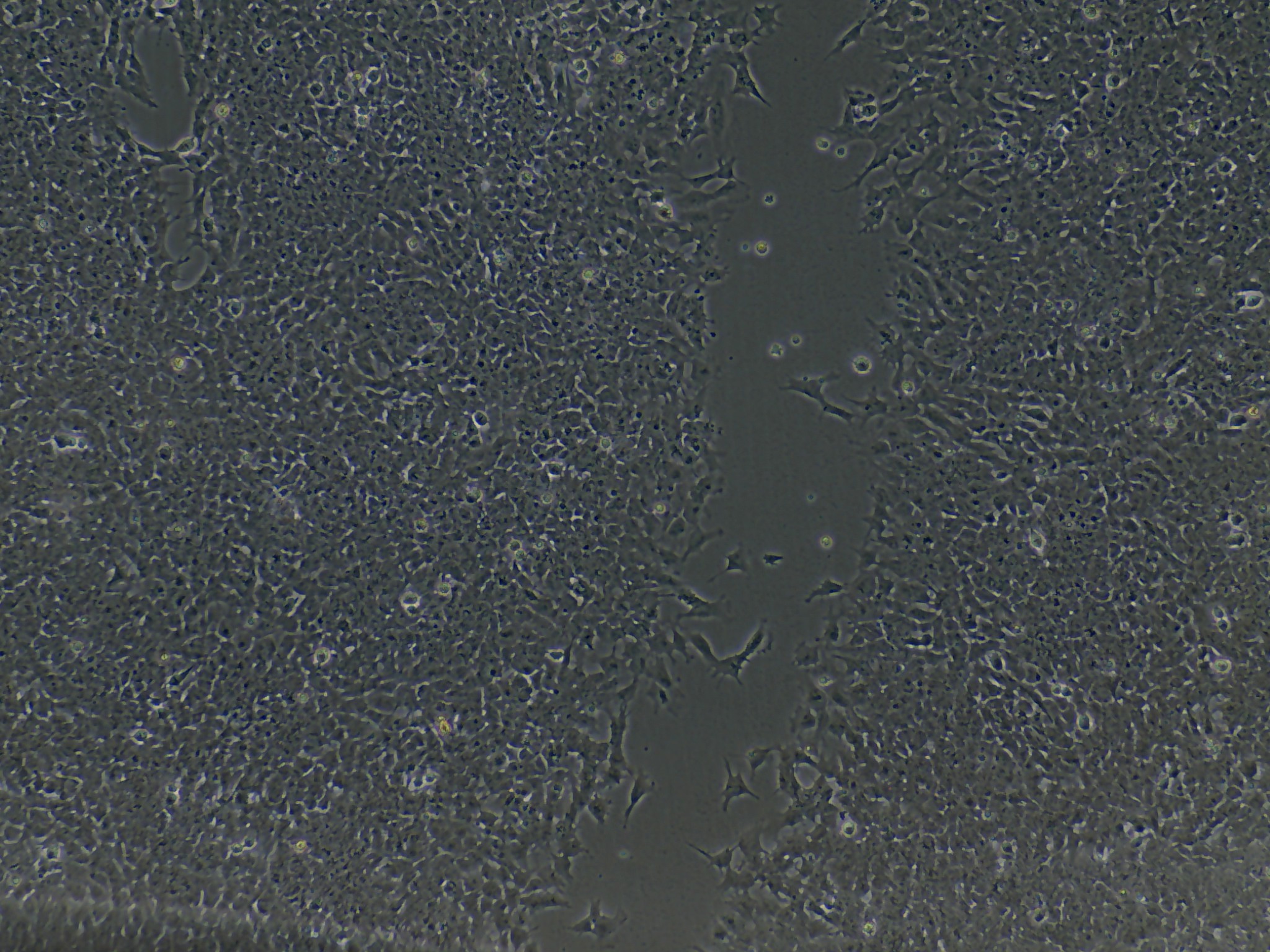

Supplement: Supplementary file 11 — Source data Fig. 4 [file 44320_2025_151_MOESM11_ESM.zip › FIGURE4/4D/250529-NCC-d5-SWA/NOG3-8h.jpg]

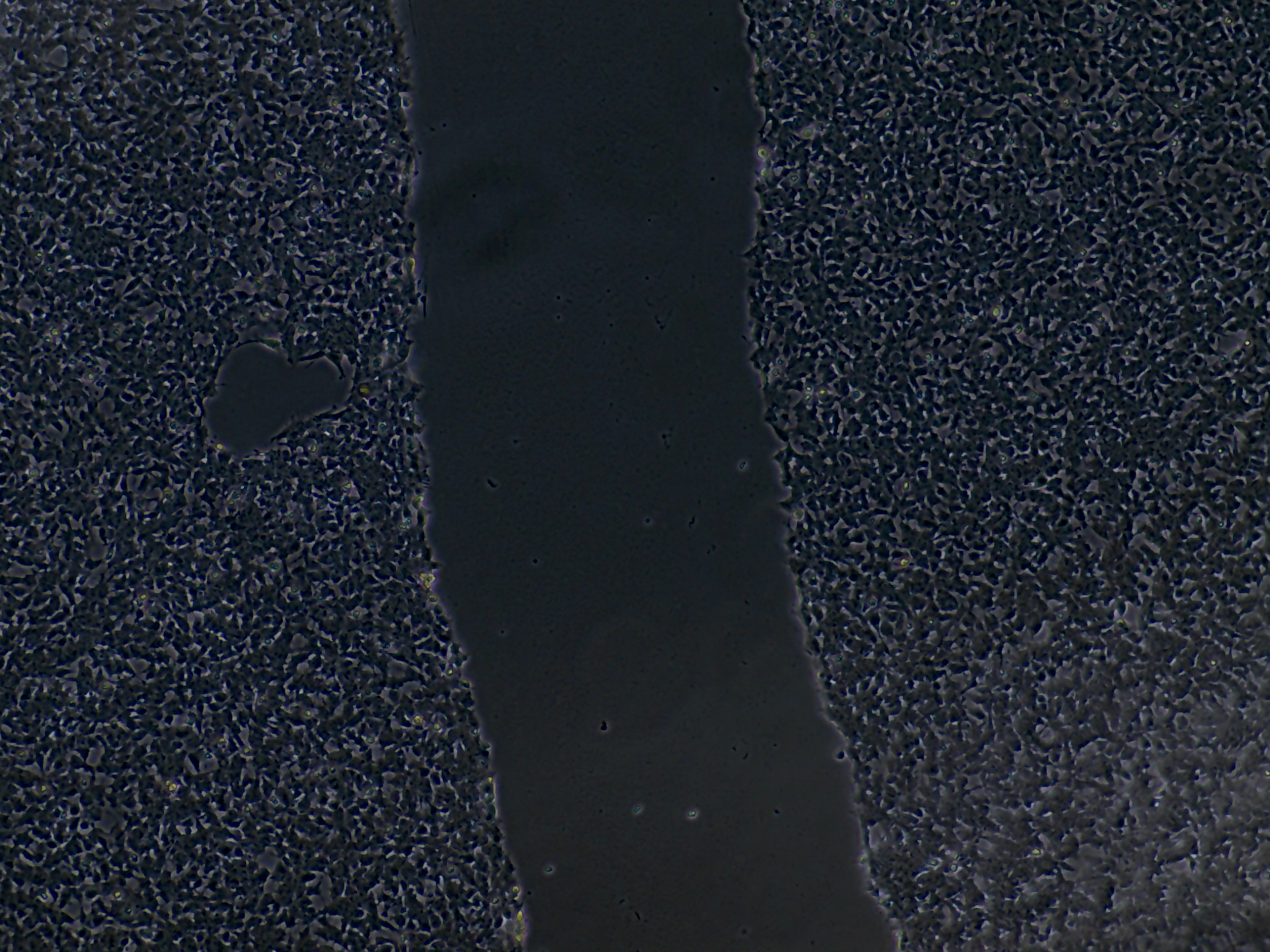

Supplement: Supplementary file 11 — Source data Fig. 4 [file 44320_2025_151_MOESM11_ESM.zip › FIGURE4/4D/250529-NCC-d5-SWA/HSTE3-0h.jpg]

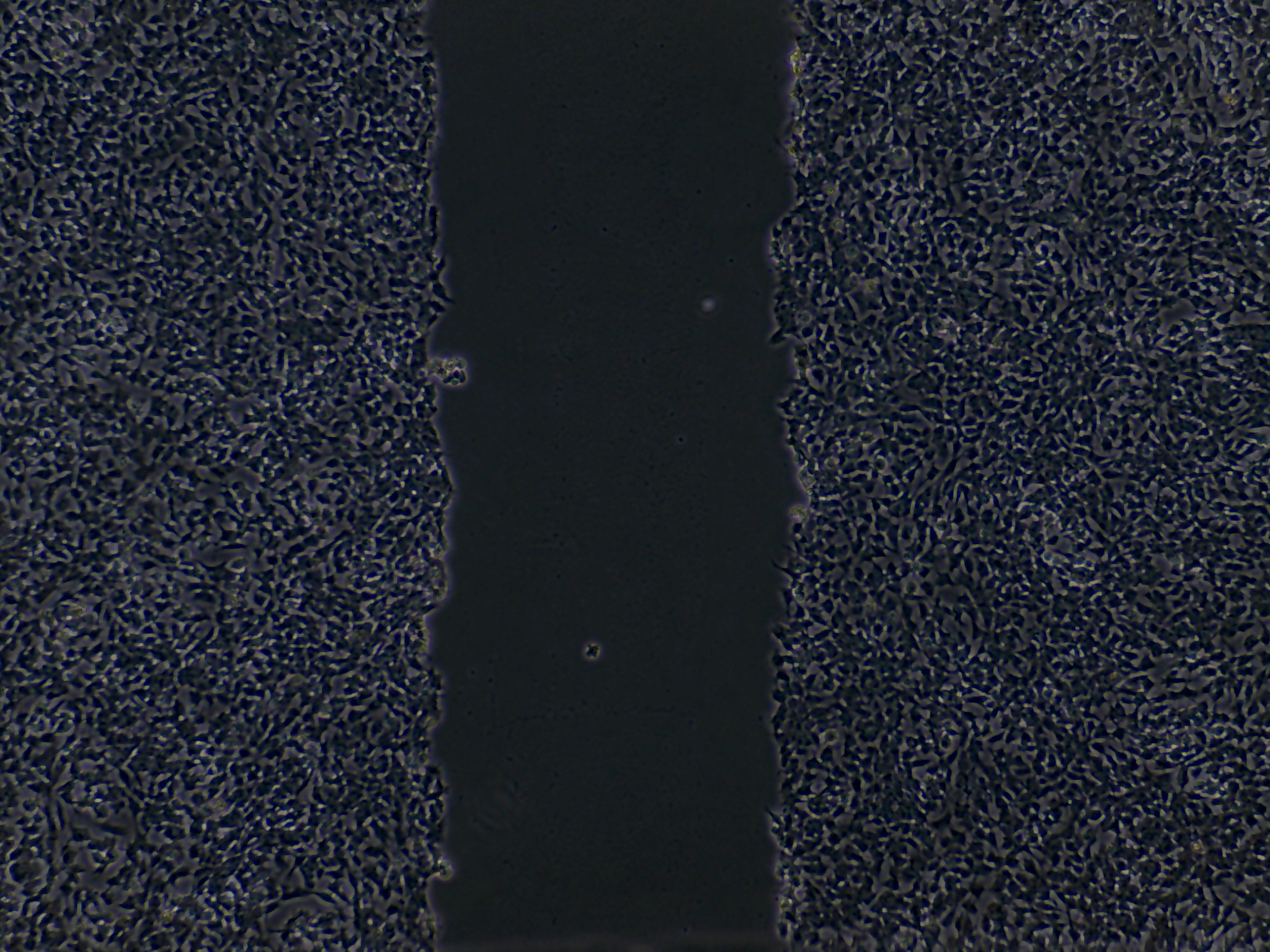

Supplement: Supplementary file 11 — Source data Fig. 4 [file 44320_2025_151_MOESM11_ESM.zip › FIGURE4/4D/250529-NCC-d5-SWA/NOG2-0h.jpg]
