# Supplementary material for: Hominoid-specific transposable elements reshaped neural crest migration in craniofacial development
Source: Mol Syst Biol. 2025 Sep 22;21(12):1731–47. doi: 10.1038/s44320-025-00151-z (PMC12673149; doi:10.1038/s44320-025-00151-z)
Supplement: Supplementary file 9 — Source data Fig. 2 [file 44320_2025_151_MOESM9_ESM.zip › FIGURE2/2D/knownResults.html]

/rds/general/user/ldeelen/home - Homer Known Motif Enrichment Results


# Homer Known Motif Enrichment Results (/rds/general/user/ldeelen/home)

Homer *de novo* Motif Results  
Gene Ontology Enrichment Results  
Known Motif Enrichment Results (txt file)  
Total Target Sequences = 187, Total Background Sequences = 1123

|  |  |  |  |  |  |  |  |  |  |  |  |
| --- | --- | --- | --- | --- | --- | --- | --- | --- | --- | --- | --- |
| Rank | Motif | Name | P-value | log P-pvalue | q-value (Benjamini) | # Target Sequences with Motif | % of Targets Sequences with Motif | # Background Sequences with Motif | % of Background Sequences with Motif | Motif File | SVG |
| 1 | C G A T C T A G A C G T A C G T A C G T C G T A A G C T C G A T A G C T C G T A C T A G T A G C | FoxD3(forkhead)/ZebrafishEmbryo-Foxd3.biotin-ChIP-seq(GSE106676)/Homer | 1e-12 | -2.866e+01 | 0.0000 | 61.0 | 32.62% | 120.1 | 10.69% | motif file (matrix) | svg |
| 2 | G C T A T C G A A C T G G T C A C G T A A C G T C G T A C G A T G C A T G A T C | KANADI1(Myb)/Seedling-KAN1-ChIP-Seq(GSE48081)/Homer | 1e-10 | -2.356e+01 | 0.0000 | 81.0 | 43.32% | 227.0 | 20.21% | motif file (matrix) | svg |
| 3 | T C G A C T A G G C T A C G T A A C G T G T C A A C G T C G A T G A T C A G C T G C A T G C T A | At5g29000(G2like)/col-At5g29000-DAP-Seq(GSE60143)/Homer | 1e-10 | -2.334e+01 | 0.0000 | 67.0 | 35.83% | 166.0 | 14.77% | motif file (matrix) | svg |
| 4 | C T A G A C T G T G C A C G A T C G T A C T G A A T C G A C T G G A C T A G C T | At1g49010(MYBrelated)/col-At1g49010-DAP-Seq(GSE60143)/Homer | 1e-10 | -2.308e+01 | 0.0000 | 117.0 | 62.57% | 420.0 | 37.38% | motif file (matrix) | svg |
| 5 | C G T A C G T A C T G A A C T G C G T A C G T A A C G T G T C A A C G T G C A T A G T C G A C T | At2g03500(G2like)/col-At2g03500-DAP-Seq(GSE60143)/Homer | 1e-9 | -2.296e+01 | 0.0000 | 68.0 | 36.36% | 172.0 | 15.30% | motif file (matrix) | svg |
| 6 | C G T A C G T A T C G A A C T G C G T A C G T A A C G T C G T A A C G T C G A T A G T C A G C T G C A T G C A T C G A T | AT2G20400(G2like)/colamp-AT2G20400-DAP-Seq(GSE60143)/Homer | 1e-9 | -2.238e+01 | 0.0000 | 66.0 | 35.29% | 165.3 | 14.72% | motif file (matrix) | svg |
| 7 | C G T A G A C T C G T A A C G T C A G T A G T C A G C T G A C T | KAN2(G2like)/colamp-KAN2-DAP-Seq(GSE60143)/Homer | 1e-9 | -2.126e+01 | 0.0000 | 97.0 | 51.87% | 320.8 | 28.55% | motif file (matrix) | svg |
| 8 | C T G A C T G A A G T C T A G C G A C T G C A T C T G A A G C T A G T C A G T C | At5g08520(MYBrelated)/colamp-At5g08520-DAP-Seq(GSE60143)/Homer | 1e-9 | -2.083e+01 | 0.0000 | 135.0 | 72.19% | 545.6 | 48.56% | motif file (matrix) | svg |
| 9 | C T A G C T A G G C A T C G A T C T G A G T C A G C T A A T G C C G T A C A G T G A C T C G T A T G C A | Hnf1(Homeobox)/Liver-Foxa2-Chip-Seq(GSE25694)/Homer | 1e-9 | -2.081e+01 | 0.0000 | 122.0 | 65.24% | 464.3 | 41.32% | motif file (matrix) | svg |
| 10 | C T A G A C G T G C A T T C G A G T C A G C A T A T C G C G T A C A G T A G C T C T G A T G C A | HNF1b(Homeobox)/PDAC-HNF1B-ChIP-Seq(GSE64557)/Homer | 1e-8 | -2.053e+01 | 0.0000 | 120.0 | 64.17% | 454.0 | 40.41% | motif file (matrix) | svg |
| 11 | C G A T T C G A A C T G G T C A C G T A C G A T G T A C G A C T | At3g04030(G2like)/col-At3g04030-DAP-Seq(GSE60143)/Homer | 1e-8 | -2.041e+01 | 0.0000 | 73.0 | 39.04% | 206.8 | 18.40% | motif file (matrix) | svg |
| 12 | G C A T G C A T G C T A G C A T G A T C C G A T A C G T C G T A A C G T A G T C G A C T G C A T G C A T G C A T G C A T | At5g58900(MYBrelated)/colamp-At5g58900-DAP-Seq(GSE60143)/Homer | 1e-8 | -1.997e+01 | 0.0000 | 120.0 | 64.17% | 458.6 | 40.81% | motif file (matrix) | svg |
| 13 | C T A G C G T A G T C A C G T A A G T C G A T C A G C T C T A G C G T A A C G T G T C A G A T C | Six2(Homeobox)/NephronProgenitor-Six2-ChIP-Seq(GSE39837)/Homer | 1e-8 | -1.965e+01 | 0.0000 | 96.0 | 51.34% | 325.0 | 28.93% | motif file (matrix) | svg |
| 14 | G T A C C G T A C G T A A C G T C G T A C G T A A C G T C G A T | ATHB53(HB)/col-ATHB53-DAP-Seq(GSE60143)/Homer | 1e-8 | -1.943e+01 | 0.0000 | 33.0 | 17.65% | 51.4 | 4.57% | motif file (matrix) | svg |
| 15 | A T G C T C G A T A C G A C G T A T G C A G T C A C G T A G T C A G T C G A T C | Znf263(Zf)/K562-Znf263-ChIP-Seq(GSE31477)/Homer | 1e-8 | -1.923e+01 | 0.0000 | 88.0 | 47.06% | 286.4 | 25.49% | motif file (matrix) | svg |
| 16 | C A T G G C T A C T A G T A C G C G T A T C A G C G T A A C T G C G T A C A T G C T G A C G T A | BPC1(BBRBPC)/colamp-BPC1-DAP-Seq(GSE60143)/Homer | 1e-8 | -1.912e+01 | 0.0000 | 109.0 | 58.29% | 400.3 | 35.62% | motif file (matrix) | svg |
| 17 | A G T C A G T C C G T A C G T A A C G T C G T A C G T A A C G T | ATHB21(HB)/colamp-ATHB21-DAP-Seq(GSE60143)/Homer | 1e-8 | -1.855e+01 | 0.0000 | 29.0 | 15.51% | 41.6 | 3.71% | motif file (matrix) | svg |
| 18 | G A C T G A T C G A C T A C G T C G T A A C G T A G T C A G T C C G T A G C A T G C T A G C A T | At1g74840(MYBrelated)/col100-At1g74840-DAP-Seq(GSE60143)/Homer | 1e-7 | -1.810e+01 | 0.0000 | 128.0 | 68.45% | 521.8 | 46.44% | motif file (matrix) | svg |
| 19 | G C A T G C A T C G A T C G T A C T G A C T A G A C T G C T A G G A T C G C T A G A C T G C A T G C A T G C A T G A C T | AT2G33550(Trihelix)/colamp-AT2G33550-DAP-Seq(GSE60143)/Homer | 1e-7 | -1.790e+01 | 0.0000 | 101.0 | 54.01% | 364.6 | 32.45% | motif file (matrix) | svg |
| 20 | C T A G C T G A C G T A C G T A C G T A C G T A A C T G A C G T C T A G G T C A | COG1(C2C2dof)/col-COG1-DAP-Seq(GSE60143)/Homer | 1e-7 | -1.760e+01 | 0.0000 | 72.0 | 38.50% | 217.4 | 19.35% | motif file (matrix) | svg |
| 21 | C G A T C G T A G C A T C T A G A C T G C G T A A C G T G T C A C G T A C T A G C T A G G C A T | At1g19000(MYBrelated)/colamp-At1g19000-DAP-Seq(GSE60143)/Homer | 1e-7 | -1.748e+01 | 0.0000 | 63.0 | 33.69% | 176.9 | 15.74% | motif file (matrix) | svg |
| 22 | G C T A C G T A A C G T C A T G C G T A A C G T A C G T C T A G | ATHB5(HB)/colamp-ATHB5-DAP-Seq(GSE60143)/Homer | 1e-7 | -1.744e+01 | 0.0000 | 36.0 | 19.25% | 67.9 | 6.04% | motif file (matrix) | svg |
| 23 | A G T C G T C A G C T A C G A T T G C A C G T A C G A T C A G T A T C G C T A G | ATHB6(Homeobox)/Arabidopsis-HB6-ChIP-Seq(GSE80564)/Homer | 1e-7 | -1.744e+01 | 0.0000 | 36.0 | 19.25% | 67.4 | 6.00% | motif file (matrix) | svg |
| 24 | T A G C G T A C C A T G A C T G C T G A C G T A G C T A G C A T G A C T T G A C G T A C A C T G A T C G C G T A C G T A | LBD19(LOBAS2)/colamp-LBD19-DAP-Seq(GSE60143)/Homer | 1e-7 | -1.730e+01 | 0.0000 | 96.0 | 51.34% | 341.1 | 30.36% | motif file (matrix) | svg |
| 25 | C G T A C G T A G A C T C T A G A C T G C G T A A C G T T C G A C T G A C A T G C T A G G C A T | AT4G00250(GeBP)/col-AT4G00250-DAP-Seq(GSE60143)/Homer | 1e-7 | -1.730e+01 | 0.0000 | 61.0 | 32.62% | 168.3 | 14.98% | motif file (matrix) | svg |
| 26 | C G T A G A C T C G A T A T C G G T A C G C A T C A T G C G T A T A C G G C A T G T A C C G T A C A T G A T G C G C T A C T A G G C A T G C A T G C A T G A C T | MafB(bZIP)/BMM-Mafb-ChIP-Seq(GSE75722)/Homer | 1e-7 | -1.674e+01 | 0.0000 | 96.0 | 51.34% | 345.3 | 30.73% | motif file (matrix) | svg |
| 27 | C A G T G A C T C G T A G C T A G T A C G A T C G T A C G A C T A G C T A C G T T G A C C G T A C A G T A C G T A T G C | ZNF652/HepG2-ZNF652.Flag-ChIP-Seq(Encode)/Homer | 1e-7 | -1.661e+01 | 0.0000 | 96.0 | 51.34% | 346.5 | 30.84% | motif file (matrix) | svg |
| 28 | A C T G A T C G A G T C A C G T C G T A A G T C A G T C A C G T A C T G C G T A | Zelda(Zf)/Embryo-zld-ChIP-Seq(GSE65441)/Homer | 1e-7 | -1.659e+01 | 0.0000 | 27.0 | 14.44% | 40.3 | 3.59% | motif file (matrix) | svg |
| 29 | A G T C C G T A C G A T A G T C G T C A A G T C A C G T C T G A | Unknown2/Drosophila-Promoters/Homer | 1e-7 | -1.657e+01 | 0.0000 | 165.0 | 88.24% | 794.0 | 70.67% | motif file (matrix) | svg |
| 30 | G A T C G A T C G C T A C G T A A C G T C G T A C G T A A C G T C A G T C A T G | ATHB40(HB)/col-ATHB40-DAP-Seq(GSE60143)/Homer | 1e-7 | -1.612e+01 | 0.0000 | 35.0 | 18.72% | 68.5 | 6.09% | motif file (matrix) | svg |
| 31 | G T C A C G T A A C G T C G A T C G T A A C G T A C G T C T A G | LMI1(HB)/colamp-LMI1-DAP-Seq(GSE60143)/Homer | 1e-7 | -1.612e+01 | 0.0000 | 35.0 | 18.72% | 68.3 | 6.08% | motif file (matrix) | svg |
| 32 | G C T A C G T A A C G T A T C G C G T A A C G T A C G T C T A G | ATHB6(Homeobox)/col-ATHB6-DAP-Seq(GSE60143)/Homer | 1e-6 | -1.592e+01 | 0.0000 | 37.0 | 19.79% | 76.5 | 6.81% | motif file (matrix) | svg |
| 33 | G C A T C T A G C T A G C G T A A G C T C G T A C T G A C A T G C T A G G C A T | AT5G56840(MYBrelated)/colamp-AT5G56840-DAP-Seq(GSE60143)/Homer | 1e-6 | -1.526e+01 | 0.0000 | 69.0 | 36.90% | 217.2 | 19.33% | motif file (matrix) | svg |
| 34 | C G A T A C G T C G T A C G T A A C G T A G C T T G C A C T G A C G T A A C G T A C G T C G T A | Phox2b(Homeobox)/CLBGA-PHOX2B-ChIP-Seq(GSE90683)/Homer | 1e-6 | -1.521e+01 | 0.0000 | 116.0 | 62.03% | 472.0 | 42.01% | motif file (matrix) | svg |
| 35 | G C T A C G A T C G A T A C G T G C A T C G T A G A T C G A T C A C T G A G C T | GTL1(Trihelix)/colamp-GTL1-DAP-Seq(GSE60143)/Homer | 1e-6 | -1.492e+01 | 0.0000 | 110.0 | 58.82% | 438.5 | 39.03% | motif file (matrix) | svg |
| 36 | G A T C G C A T C T G A C G T A A G C T A G C T C G T A T C G A C T G A A C G T G C A T C G T A | Phox2a(Homeobox)/Neuron-Phox2a-ChIP-Seq(GSE31456)/Homer | 1e-6 | -1.486e+01 | 0.0000 | 116.0 | 62.03% | 474.1 | 42.20% | motif file (matrix) | svg |
| 37 | G T C A C T A G A G C T T C G A T G C A C A G T A G C T C G T A C T G A A C G T | ATHB33(ZFHD)/col-ATHB33-DAP-Seq(GSE60143)/Homer | 1e-6 | -1.478e+01 | 0.0000 | 44.0 | 23.53% | 108.8 | 9.69% | motif file (matrix) | svg |
| 38 | G T A C G C A T A C G T C G T A A C G T A G T C A G T C C T G A | At5g47390(MYBrelated)/col-At5g47390-DAP-Seq(GSE60143)/Homer | 1e-6 | -1.469e+01 | 0.0000 | 67.0 | 35.83% | 211.9 | 18.86% | motif file (matrix) | svg |
| 39 | A G C T G C A T G T C A C G A T T A G C C G T A A C G T G C T A | CRC(C2C2YABBY)/col-CRC-DAP-Seq(GSE60143)/Homer | 1e-6 | -1.445e+01 | 0.0000 | 65.0 | 34.76% | 203.0 | 18.07% | motif file (matrix) | svg |
| 40 | G A T C G C A T A C G T C G T A A C G T A G T C A G T C C G T A | AT5G61620(MYBrelated)/colamp-AT5G61620-DAP-Seq(GSE60143)/Homer | 1e-6 | -1.436e+01 | 0.0000 | 68.0 | 36.36% | 218.2 | 19.42% | motif file (matrix) | svg |
| 41 | C G T A C T A G A C T G A C T G G A C T C T A G C A G T C T A G C A T G G A T C | KLF5(Zf)/LoVo-KLF5-ChIP-Seq(GSE49402)/Homer | 1e-6 | -1.408e+01 | 0.0000 | 154.0 | 82.35% | 730.4 | 65.01% | motif file (matrix) | svg |
| 42 | G T A C C G T A C G T A A C G T G C T A C G T A A C G T C A G T | ATHB13(Homeobox)/col-ATHB13-DAP-Seq(GSE60143)/Homer | 1e-6 | -1.396e+01 | 0.0000 | 36.0 | 19.25% | 80.4 | 7.15% | motif file (matrix) | svg |
| 43 | A C G T A G T C A G C T A G T C C G T A G T A C A G T C C G A T C G T A G T C A | MYB41(MYB)/col-MYB41-DAP-Seq(GSE60143)/Homer | 1e-5 | -1.339e+01 | 0.0000 | 65.0 | 34.76% | 210.3 | 18.72% | motif file (matrix) | svg |
| 44 | G T C A C G T A A C G T A T C G C G T A A C G T A C G T C T A G | ATHB7(Homeobox)/col-ATHB7-DAP-Seq(GSE60143)/Homer | 1e-5 | -1.323e+01 | 0.0000 | 29.0 | 15.51% | 57.4 | 5.11% | motif file (matrix) | svg |
| 45 | A T C G G A C T C G A T C G T A C G T A C A G T G A T C G A T C G A T C A G C T | Otx2(Homeobox)/EpiLC-Otx2-ChIP-Seq(GSE56098)/Homer | 1e-5 | -1.295e+01 | 0.0001 | 167.0 | 89.30% | 839.3 | 74.70% | motif file (matrix) | svg |
| 46 | G C T A G A C T G A C T T G C A C G T A A G T C C G T A T A G C G A T C G A C T | Eomes(T-box)/H9-Eomes-ChIP-Seq(GSE26097)/Homer | 1e-5 | -1.230e+01 | 0.0001 | 161.0 | 86.10% | 798.5 | 71.07% | motif file (matrix) | svg |
| 47 | T G A C C T G A C T A G T C G A C T G A A T G C C G T A A C T G G C A T G T A C G C A T A T C G G C A T A G C T G A T C | PR(NR)/T47D-PR-ChIP-Seq(GSE31130)/Homer | 1e-5 | -1.216e+01 | 0.0001 | 166.0 | 88.77% | 838.5 | 74.63% | motif file (matrix) | svg |
| 48 | A G C T A G T C A G T C A C G T C T A G A C G T A C G T A C G T C G T A A G T C G A T C C G T A | FOXP1(Forkhead)/H9-FOXP1-ChIP-Seq(GSE31006)/Homer | 1e-5 | -1.209e+01 | 0.0001 | 68.0 | 36.36% | 235.0 | 20.91% | motif file (matrix) | svg |
| 49 | C G T A C G T A C T G A A C T G C G T A C G T A A C G T C T A G C G A T C G A T | AT2G38300(G2like)/col-AT2G38300-DAP-Seq(GSE60143)/Homer | 1e-5 | -1.199e+01 | 0.0001 | 157.0 | 83.96% | 771.8 | 68.69% | motif file (matrix) | svg |
| 50 | G T A C A C T G A C G T T C A G G C A T C G T A C G A T G C A T C G T A A G T C C G T A T G A C C A T G G A C T G C T A | ANAC083(NAC)/col-ANAC083-DAP-Seq(GSE60143)/Homer | 1e-5 | -1.195e+01 | 0.0001 | 34.0 | 18.18% | 81.4 | 7.25% | motif file (matrix) | svg |
| 51 | T A G C G T A C C G T A C T A G A C T G T G C A C G T A A T G C C G T A A T C G | AR-halfsite(NR)/LNCaP-AR-ChIP-Seq(GSE27824)/Homer | 1e-5 | -1.194e+01 | 0.0001 | 172.0 | 91.98% | 889.1 | 79.13% | motif file (matrix) | svg |
| 52 | G T A C T G A C A T G C A G T C A C T G G A T C A T C G G A T C | SUT1?/SacCer-Promoters/Homer | 1e-5 | -1.172e+01 | 0.0002 | 177.0 | 94.65% | 935.4 | 83.25% | motif file (matrix) | svg |
| 53 | C T G A C G A T C A T G A T C G G C A T C A T G G C T A A G T C | ASHR1(ND)/col-ASHR1-DAP-Seq(GSE60143)/Homer | 1e-5 | -1.165e+01 | 0.0002 | 159.0 | 85.03% | 789.6 | 70.28% | motif file (matrix) | svg |
| 54 | T C G A C G T A C G T A T C G A A C T G G T A C C G T A A G C T G T C A G C A T | At3g24120(G2like)/col-At3g24120-DAP-Seq(GSE60143)/Homer | 1e-4 | -1.149e+01 | 0.0002 | 169.0 | 90.37% | 868.9 | 77.34% | motif file (matrix) | svg |
| 55 | C G T A C T G A A C T G A G C T A G T C G A T C G A T C G C A T C T G A C T A G C T A G T A C G T C G A T G C A G C A T | EBF2(EBF)/BrownAdipose-EBF2-ChIP-Seq(GSE97114)/Homer | 1e-4 | -1.138e+01 | 0.0002 | 99.0 | 52.94% | 406.8 | 36.20% | motif file (matrix) | svg |
| 56 | A T G C G A C T A C G T C T A G A C G T A C G T A C G T C T G A G A T C G C T A A G C T C G T A | Foxa2(Forkhead)/Liver-Foxa2-ChIP-Seq(GSE25694)/Homer | 1e-4 | -1.137e+01 | 0.0002 | 55.0 | 29.41% | 176.8 | 15.74% | motif file (matrix) | svg |
| 57 | T A C G T A G C G C T A C G A T C T A G A C G T C A G T C A G T G C T A A G T C G T C A G C A T | FOXK2(Forkhead)/U2OS-FOXK2-ChIP-Seq(E-MTAB-2204)/Homer | 1e-4 | -1.134e+01 | 0.0002 | 105.0 | 56.15% | 441.4 | 39.28% | motif file (matrix) | svg |
| 58 | A C T G C A G T A C G T C G T A C G T A A C G T A C T G C T G A | Nkx6.1(Homeobox)/Islet-Nkx6.1-ChIP-Seq(GSE40975)/Homer | 1e-4 | -1.122e+01 | 0.0002 | 101.0 | 54.01% | 419.9 | 37.38% | motif file (matrix) | svg |
| 59 | A G T C C G A T C T G A C G T A A C G T C A G T T C A G T G A C | Isl1(Homeobox)/Neuron-Isl1-ChIP-Seq(GSE31456)/Homer | 1e-4 | -1.116e+01 | 0.0002 | 166.0 | 88.77% | 847.4 | 75.42% | motif file (matrix) | svg |
| 60 | T C A G A G C T G T A C C G T A A C G T C G T A C G T A C G T A G C T A G A C T | Cdx2(Homeobox)/mES-Cdx2-ChIP-Seq(GSE14586)/Homer | 1e-4 | -1.112e+01 | 0.0002 | 73.0 | 39.04% | 267.8 | 23.83% | motif file (matrix) | svg |
| 61 | T C G A C T G A C G T A C G T A C G T A C G T A A C T G A C G T C G A T C T G A | BBX31(Orphan)/col-BBX31-DAP-Seq(GSE60143)/Homer | 1e-4 | -1.110e+01 | 0.0002 | 83.0 | 44.39% | 320.9 | 28.56% | motif file (matrix) | svg |
| 62 | A T G C A G T C G C A T A G C T A C G T T C A G C G A T A G C T G A T C A T C G | Sox10(HMG)/SciaticNerve-Sox3-ChIP-Seq(GSE35132)/Homer | 1e-4 | -1.108e+01 | 0.0003 | 165.0 | 88.24% | 840.7 | 74.82% | motif file (matrix) | svg |
| 63 | T C G A T G C A A C T G G T C A A G T C A G T C A G T C A G T C A G C T T G A C | LRF(Zf)/Erythroblasts-ZBTB7A-ChIP-Seq(GSE74977)/Homer | 1e-4 | -1.103e+01 | 0.0003 | 74.0 | 39.57% | 273.6 | 24.35% | motif file (matrix) | svg |
| 64 | A T G C C G T A C G T A C G T A C G T A C G T A A C T G A C G T C G A T C T G A | dof43(C2C2dof)/colamp-dof43-DAP-Seq(GSE60143)/Homer | 1e-4 | -1.102e+01 | 0.0003 | 81.0 | 43.32% | 310.9 | 27.67% | motif file (matrix) | svg |
| 65 | C G A T T A C G T G A C G A C T C A T G C G T A T A C G A C G T G T A C C T G A | Bach2(bZIP)/OCILy7-Bach2-ChIP-Seq(GSE44420)/Homer | 1e-4 | -1.100e+01 | 0.0003 | 107.0 | 57.22% | 456.7 | 40.65% | motif file (matrix) | svg |
| 66 | A G C T A C G T A C T G A T G C A G T C C G T A C T G A T A C G | NF1-halfsite(CTF)/LNCaP-NF1-ChIP-Seq(Unpublished)/Homer | 1e-4 | -1.087e+01 | 0.0003 | 72.0 | 38.50% | 264.5 | 23.54% | motif file (matrix) | svg |
| 67 | A G C T G A T C G A T C C G T A G T A C A G T C C G A T C T G A G T A C G A T C C G T A G A C T | ATY19(MYB)/col-ATY19-DAP-Seq(GSE60143)/Homer | 1e-4 | -1.079e+01 | 0.0003 | 64.0 | 34.22% | 224.2 | 19.96% | motif file (matrix) | svg |
| 68 | C T G A T C A G G T A C T G C A A G T C C G T A A G T C A C T G A C G T A C T G | MNT(bHLH)/HepG2-MNT-ChIP-Seq(Encode)/Homer | 1e-4 | -1.073e+01 | 0.0003 | 166.0 | 88.77% | 851.6 | 75.79% | motif file (matrix) | svg |
| 69 | C A T G A C G T C T A G A C G T C A G T A C G T C T A G A G T C | PHA-4(Forkhead)/cElegans-Embryos-PHA4-ChIP-Seq(modEncode)/Homer | 1e-4 | -1.073e+01 | 0.0003 | 170.0 | 90.91% | 883.4 | 78.63% | motif file (matrix) | svg |
| 70 | C T A G A C T G A C G T C G T A A C T G A C T G A C G T T C A G C T G A T C G A | MYB107(MYB)/col-MYB107-DAP-Seq(GSE60143)/Homer | 1e-4 | -1.073e+01 | 0.0003 | 71.0 | 37.97% | 260.8 | 23.22% | motif file (matrix) | svg |
| 71 | C G T A A C T G C G T A A C G T C A G T A G T C A G C T G C A T G C T A C G A T | At2g01060(G2like)/colamp-At2g01060-DAP-Seq(GSE60143)/Homer | 1e-4 | -1.072e+01 | 0.0003 | 168.0 | 89.84% | 867.3 | 77.20% | motif file (matrix) | svg |
| 72 | A G C T C A T G G C A T G A T C T G C A C T A G G A T C A C G T | Tgif2(Homeobox)/mES-Tgif2-ChIP-Seq(GSE55404)/Homer | 1e-4 | -1.069e+01 | 0.0003 | 145.0 | 77.54% | 699.6 | 62.26% | motif file (matrix) | svg |
| 73 | T C A G C T A G C T A G T G C A C G A T C G A T C G T A C T A G | GSC(Homeobox)/FrogEmbryos-GSC-ChIP-Seq(DRA000576)/Homer | 1e-4 | -1.068e+01 | 0.0003 | 174.0 | 93.05% | 917.1 | 81.62% | motif file (matrix) | svg |
| 74 | T A C G T C A G G A T C G T A C T C G A G A C T G C T A G C T A G C T A C G T A G A T C G T C A | CDX4(Homeobox)/ZebrafishEmbryos-Cdx4.Myc-ChIP-Seq(GSE48254)/Homer | 1e-4 | -1.056e+01 | 0.0004 | 74.0 | 39.57% | 277.4 | 24.69% | motif file (matrix) | svg |
| 75 | C T G A A G T C C G A T A G C T A T G C G T A C A C G T A T C G C A G T G C A T | Elf4(ETS)/BMDM-Elf4-ChIP-Seq(GSE88699)/Homer | 1e-4 | -1.042e+01 | 0.0004 | 156.0 | 83.42% | 779.3 | 69.36% | motif file (matrix) | svg |
| 76 | C T A G C A T G G A C T C G T A C T A G A C T G A C G T C T A G C T A G T C A G | MYB17(MYB)/colamp-MYB17-DAP-Seq(GSE60143)/Homer | 1e-4 | -1.041e+01 | 0.0004 | 62.0 | 33.16% | 217.5 | 19.36% | motif file (matrix) | svg |
| 77 | A G C T T G A C C G A T C G A T C T A G A C G T C A G T C A G T G C T A A G T C | FOXK1(Forkhead)/HEK293-FOXK1-ChIP-Seq(GSE51673)/Homer | 1e-4 | -1.035e+01 | 0.0004 | 162.0 | 86.63% | 824.8 | 73.41% | motif file (matrix) | svg |
| 78 | C G T A C G A T C G A T C G T A C T G A G T C A A G T C A G T C A C G T C G T A C T G A G C A T C G A T C G A T G C T A | AT1G72740(MYBrelated)/colamp-AT1G72740-DAP-Seq(GSE60143)/Homer | 1e-4 | -1.033e+01 | 0.0004 | 91.0 | 48.66% | 371.6 | 33.07% | motif file (matrix) | svg |
| 79 | C T A G C A G T T G A C C G T A G A T C T C A G G A C T C A T G | BMAL1(bHLH)/Liver-Bmal1-ChIP-Seq(GSE39860)/Homer | 1e-4 | -1.029e+01 | 0.0004 | 167.0 | 89.30% | 863.0 | 76.81% | motif file (matrix) | svg |
| 80 | T C G A C T G A T A G C C T A G A C T G G T C A A C G T A G C T C G T A C T A G T A C G A C G T | bcd(Homeobox)/Embryo-Bcd-ChIP-Seq(GSE86966)/Homer | 1e-4 | -1.029e+01 | 0.0004 | 170.0 | 90.91% | 887.0 | 78.95% | motif file (matrix) | svg |
| 81 | C G T A G C T A C G A T C T A G A C G T G T C A C G T A C G T A A G T C C G T A T G C A T A C G | FoxL2(Forkhead)/Ovary-FoxL2-ChIP-Seq(GSE60858)/Homer | 1e-4 | -1.025e+01 | 0.0004 | 75.0 | 40.11% | 285.1 | 25.37% | motif file (matrix) | svg |
| 82 | C A G T T C G A A G T C A C G T A C G T T C A G C G A T G C T A G C T A C G T A G C A T A G T C C G T A T G C A A C T G | ANAC045(NAC)/col-ANAC045-DAP-Seq(GSE60143)/Homer | 1e-4 | -1.024e+01 | 0.0004 | 48.0 | 25.67% | 151.5 | 13.49% | motif file (matrix) | svg |
| 83 | A G C T G C A T C T G A T G C A A G T C G A T C T C G A C A G T C T G A T C A G | AT3G25990(Trihelix)/colamp-AT3G25990-DAP-Seq(GSE60143)/Homer | 1e-4 | -1.020e+01 | 0.0004 | 123.0 | 65.78% | 561.4 | 49.97% | motif file (matrix) | svg |
| 84 | A T C G G A T C G C A T C G T A G T C A A C G T A T G C A G T C | CRX(Homeobox)/Retina-Crx-ChIP-Seq(GSE20012)/Homer | 1e-4 | -1.016e+01 | 0.0005 | 173.0 | 92.51% | 913.0 | 81.26% | motif file (matrix) | svg |
| 85 | G A T C C T G A A G T C A G C T A C G T A C G T A C G T A C G T | At1g64620(C2C2dof)/colamp-At1g64620-DAP-Seq(GSE60143)/Homer | 1e-4 | -1.016e+01 | 0.0005 | 79.0 | 42.25% | 307.8 | 27.40% | motif file (matrix) | svg |
| 86 | A G T C C T A G C T A G A G T C G A T C G T A C A G T C C T A G A G T C A G T C A G T C G T A C | Sp2(Zf)/HEK293-Sp2.eGFP-ChIP-Seq(Encode)/Homer | 1e-4 | -1.016e+01 | 0.0005 | 137.0 | 73.26% | 651.4 | 57.98% | motif file (matrix) | svg |
| 87 | C T A G T C A G G A T C T G A C T C G A A C G T C T G A C G T A T G C A G C T A | caudal(Homeobox)/Drosophila-Embryos-ChIP-Chip(modEncode)/Homer | 1e-4 | -1.016e+01 | 0.0005 | 86.0 | 45.99% | 345.7 | 30.77% | motif file (matrix) | svg |
| 88 | A T C G A T C G A T C G A T C G A T C G A T C G A T C G A T C G A T C G A T C G | SeqBias: CG bias | 1e-4 | -1.015e+01 | 0.0005 | 182.0 | 97.33% | 996.0 | 88.65% | motif file (matrix) | svg |
| 89 | C T A G A C T G T G C A G T C A A T G C C G T A A T C G A T G C A G T C C T A G | ZNF341(Zf)/EBV-ZNF341-ChIP-Seq(GSE113194)/Homer | 1e-4 | -1.011e+01 | 0.0005 | 14.0 | 7.49% | 18.2 | 1.62% | motif file (matrix) | svg |
| 90 | G C T A T G A C G A T C C G A T G A C T A T G C C T G A A T C G G C A T A C G T | JGL(C2H2)/col-JGL-DAP-Seq(GSE60143)/Homer | 1e-4 | -1.003e+01 | 0.0005 | 118.0 | 63.10% | 532.2 | 47.36% | motif file (matrix) | svg |
| 91 | C T A G C T A G A G T C T C A G A C T G A C G T A C G T C T G A | MYB(HTH)/ERMYB-Myb-ChIPSeq(GSE22095)/Homer | 1e-4 | -1.001e+01 | 0.0005 | 159.0 | 85.03% | 805.6 | 71.70% | motif file (matrix) | svg |
| 92 | G C T A G C A T G C A T C A G T A C G T G C A T G T C A G T A C G A T C T A C G | AT5G47660(Trihelix)/colamp-AT5G47660-DAP-Seq(GSE60143)/Homer | 1e-4 | -1.000e+01 | 0.0005 | 109.0 | 58.29% | 478.1 | 42.55% | motif file (matrix) | svg |
| 93 | A T G C G A T C C G A T A C G T A C G T A C T G C A G T A G C T | Sox3(HMG)/NPC-Sox3-ChIP-Seq(GSE33059)/Homer | 1e-4 | -9.967e+00 | 0.0005 | 168.0 | 89.84% | 874.5 | 77.83% | motif file (matrix) | svg |
| 94 | C T A G T C A G C T G A T C A G T G C A A C T G T C G A T C A G | Trl(Zf)/S2-GAGAfactor-ChIP-Seq(GSE40646)/Homer | 1e-4 | -9.967e+00 | 0.0005 | 168.0 | 89.84% | 874.3 | 77.82% | motif file (matrix) | svg |
| 95 | G T A C G T A C G T C A G C T A C G T A C G T A C G T A C T A G C T A G C T A G | SEP3(MADS)/Arabidoposis-Flower-Sep3-ChIP-Seq/Homer | 1e-4 | -9.934e+00 | 0.0005 | 58.0 | 31.02% | 201.9 | 17.97% | motif file (matrix) | svg |
| 96 | T C A G G A C T A C G T C G T A A C T G A C T G A C T G A G T C C G T A G T C A | TBP3(MYBrelated)/col-TBP3-DAP-Seq(GSE60143)/Homer | 1e-4 | -9.925e+00 | 0.0005 | 145.0 | 77.54% | 707.7 | 62.99% | motif file (matrix) | svg |
| 97 | A G T C G A T C A G C T C G T A G T A C A G T C G C A T C T G A G T A C G A T C | AT4G26030(C2H2)/col-AT4G26030-DAP-Seq(GSE60143)/Homer | 1e-4 | -9.919e+00 | 0.0005 | 63.0 | 33.69% | 226.3 | 20.14% | motif file (matrix) | svg |
| 98 | G A C T G C A T C T A G C G A T G A T C T C G A C A T G G A T C | Tgif1(Homeobox)/mES-Tgif1-ChIP-Seq(GSE55404)/Homer | 1e-4 | -9.874e+00 | 0.0005 | 143.0 | 76.47% | 694.3 | 61.80% | motif file (matrix) | svg |
| 99 | G C A T C T A G A C T G A C G T C G T A A C T G A C T G C G A T C T A G T C G A T C G A G C T A | MYB40(MYB)/col-MYB40-DAP-Seq(GSE60143)/Homer | 1e-4 | -9.795e+00 | 0.0006 | 60.0 | 32.09% | 213.0 | 18.96% | motif file (matrix) | svg |
| 100 | T C A G A C T G A C G T C G T A A C T G A C T G A C G T C T A G | MYB51(MYB)/col-MYB51-DAP-Seq(GSE60143)/Homer | 1e-4 | -9.795e+00 | 0.0006 | 62.0 | 33.16% | 222.1 | 19.77% | motif file (matrix) | svg |
| 101 | G C A T G C A T C T G A A C G T C T G A A C G T C G T A C G T A C G T A A G T C G T C A G T C A | Foxf1(Forkhead)/Lung-Foxf1-ChIP-Seq(GSE77951)/Homer | 1e-4 | -9.740e+00 | 0.0006 | 162.0 | 86.63% | 830.7 | 73.93% | motif file (matrix) | svg |
| 102 | T C G A C T G A T A G C T G A C T C A G T C A G C G T A C G T A T C A G A G C T | ETV1(ETS)/GIST48-ETV1-ChIP-Seq(GSE22441)/Homer | 1e-4 | -9.713e+00 | 0.0006 | 155.0 | 82.89% | 779.3 | 69.36% | motif file (matrix) | svg |
| 103 | C G A T C T A G A C G T G T C A C G T A C G T A A G T C C G T A | Foxo3(Forkhead)/U2OS-Foxo3-ChIP-Seq(E-MTAB-2701)/Homer | 1e-4 | -9.693e+00 | 0.0006 | 161.0 | 86.10% | 823.4 | 73.29% | motif file (matrix) | svg |
| 104 | A C T G C G T A A C G T C G T A C T G A A C T G T C A G G C A T | At3g11280(MYBrelated)/col-At3g11280-DAP-Seq(GSE60143)/Homer | 1e-4 | -9.515e+00 | 0.0007 | 76.0 | 40.64% | 297.7 | 26.49% | motif file (matrix) | svg |
| 105 | C T G A T G C A A G T C A C T G A C G T T C A G C G A T G C A T G C A T G A T C G C A T G A T C G T C A A G T C A C T G | ANAC094(NAC)/col-ANAC094-DAP-Seq(GSE60143)/Homer | 1e-4 | -9.447e+00 | 0.0008 | 38.0 | 20.32% | 111.9 | 9.96% | motif file (matrix) | svg |
| 106 | G C A T A C G T A G C T A G C T A G C T C G T A A G T C A C G T | At3g60580(C2H2)/col-At3g60580-DAP-Seq(GSE60143)/Homer | 1e-4 | -9.441e+00 | 0.0008 | 177.0 | 94.65% | 954.8 | 84.98% | motif file (matrix) | svg |
| 107 | G C T A A T C G G C T A G A C T G C T A T C G A T A G C T C G A | GATA3(Zf)/iTreg-Gata3-ChIP-Seq(GSE20898)/Homer | 1e-4 | -9.438e+00 | 0.0008 | 169.0 | 90.37% | 887.3 | 78.97% | motif file (matrix) | svg |
| 108 | C G T A A G T C T G A C A G C T A C G T C G T A A C G T A G T C | At5g05790(MYBrelated)/col-At5g05790-DAP-Seq(GSE60143)/Homer | 1e-4 | -9.409e+00 | 0.0008 | 76.0 | 40.64% | 298.4 | 26.56% | motif file (matrix) | svg |
| 109 | G A C T G A T C A G T C C G T A T G A C A G T C G C A T C T G A G T A C G A T C G C A T G A C T | MYB10(MYB)/col-MYB10-DAP-Seq(GSE60143)/Homer | 1e-4 | -9.311e+00 | 0.0008 | 60.0 | 32.09% | 216.3 | 19.25% | motif file (matrix) | svg |
| 110 | C G T A A T G C C G A T A C G T A G T C C G T A C G T A C G T A C T A G A T C G | TCFL2(HMG)/K562-TCF7L2-ChIP-Seq(GSE29196)/Homer | 1e-4 | -9.288e+00 | 0.0008 | 51.0 | 27.27% | 172.1 | 15.32% | motif file (matrix) | svg |
| 111 | G A T C A G T C G A C T G C T A G T A C A G T C G C A T G C T A G T A C G A T C | MYB61(MYB)/colamp-MYB61-DAP-Seq(GSE60143)/Homer | 1e-4 | -9.270e+00 | 0.0009 | 67.0 | 35.83% | 252.8 | 22.50% | motif file (matrix) | svg |
| 112 | T C G A T G C A C A G T T C G A G A T C A G T C C G T A C G T A A C T G A G T C C G T A C G T A T C A G C G A T A G T C | AT5G25475(ABI3VP1)/col-AT5G25475-DAP-Seq(GSE60143)/Homer | 1e-4 | -9.263e+00 | 0.0009 | 160.0 | 85.56% | 820.2 | 73.00% | motif file (matrix) | svg |
| 113 | T C A G A C G T A G T C T C G A A G T C T C A G G C A T C T A G C T A G A G C T | Usf2(bHLH)/C2C12-Usf2-ChIP-Seq(GSE36030)/Homer | 1e-4 | -9.263e+00 | 0.0009 | 139.0 | 74.33% | 674.8 | 60.06% | motif file (matrix) | svg |
| 114 | C G T A C G T A G C T A C G T A G A T C C T G A A C G T A C G T A G T C A G C T G C A T G C A T | AT2G40260(G2like)/colamp-AT2G40260-DAP-Seq(GSE60143)/Homer | 1e-3 | -9.184e+00 | 0.0009 | 166.0 | 88.77% | 866.0 | 77.08% | motif file (matrix) | svg |
| 115 | C T A G A C T G A G C T C G T A A C T G A C T G A C G T C T A G | MYB99(MYB)/colamp-MYB99-DAP-Seq(GSE60143)/Homer | 1e-3 | -9.089e+00 | 0.0010 | 62.0 | 33.16% | 228.8 | 20.37% | motif file (matrix) | svg |
| 116 | C A G T A C T G T C A G T G C A G C T A A T G C T C G A A T C G G T C A T G C A | ZNF189(Zf)/HEK293-ZNF189.GFP-ChIP-Seq(GSE58341)/Homer | 1e-3 | -8.957e+00 | 0.0011 | 162.0 | 86.63% | 838.8 | 74.65% | motif file (matrix) | svg |
| 117 | G C T A T C G A C G T A C T A G A G C T G T C A G T C A C G T A A G T C C G T A | FOXA1(Forkhead)/LNCAP-FOXA1-ChIP-Seq(GSE27824)/Homer | 1e-3 | -8.941e+00 | 0.0011 | 67.0 | 35.83% | 255.4 | 22.73% | motif file (matrix) | svg |
| 118 | T G A C C T A G T C A G G T C A C G T A T C A G C G A T T C A G T C G A T G C A C T G A T A G C | PU.1-IRF(ETS:IRF)/Bcell-PU.1-ChIP-Seq(GSE21512)/Homer | 1e-3 | -8.937e+00 | 0.0011 | 168.0 | 89.84% | 884.1 | 78.68% | motif file (matrix) | svg |
| 119 | C G T A A C T G C G T A A C G T C G T A C T G A T C A G T G C A A G C T T G A C | TRPS1(Zf)/MCF7-TRPS1-ChIP-Seq(GSE107013)/Homer | 1e-3 | -8.937e+00 | 0.0011 | 168.0 | 89.84% | 884.6 | 78.73% | motif file (matrix) | svg |
| 120 | C G T A C G T A C T G A C A G T T A G C C G T A G A T C C T A G G C A T C A T G G T A C G A C T | BIM2(bHLH)/col-BIM2-DAP-Seq(GSE60143)/Homer | 1e-3 | -8.882e+00 | 0.0012 | 160.0 | 85.56% | 824.2 | 73.36% | motif file (matrix) | svg |
| 121 | T A G C G C T A A C T G C G T A A C G T C G T A C G T A T A C G T C A G T C G A | Gata1(Zf)/K562-GATA1-ChIP-Seq(GSE18829)/Homer | 1e-3 | -8.852e+00 | 0.0012 | 61.0 | 32.62% | 225.4 | 20.07% | motif file (matrix) | svg |
| 122 | A G T C C G T A T G A C A T G C G C A T C T G A G T A C G A T C | MYB55(MYB)/colamp-MYB55-DAP-Seq(GSE60143)/Homer | 1e-3 | -8.852e+00 | 0.0012 | 61.0 | 32.62% | 225.0 | 20.03% | motif file (matrix) | svg |
| 123 | G T A C G C A T C T G A C G T A G A C T A G C T C A T G T G C A C T G A A C G T G A C T C G T A | Prop1(Homeobox)/GHFT1-PROP1.biotin-ChIP-Seq(GSE77302)/Homer | 1e-3 | -8.847e+00 | 0.0012 | 136.0 | 72.73% | 659.8 | 58.73% | motif file (matrix) | svg |
| 124 | G C T A G C A T G C T A G C A T G C A T C G T A C G T A A G T C A G T C A C T G G C A T G C A T C G T A G C T A G C T A | MYB73(MYB)/col-MYB73-DAP-Seq(GSE60143)/Homer | 1e-3 | -8.821e+00 | 0.0012 | 165.0 | 88.24% | 862.8 | 76.79% | motif file (matrix) | svg |
| 125 | C T A G T A C G G A C T T G C A T G C A C G A T T A C G C T G A T C G A C T G A | Hoxa10(Homeobox)/ChickenMSG-Hoxa10.Flag-ChIP-Seq(GSE86088)/Homer | 1e-3 | -8.683e+00 | 0.0014 | 138.0 | 73.80% | 674.5 | 60.03% | motif file (matrix) | svg |
| 126 | T G C A C T G A A G T C G T C A A C T G A C T G C G T A C G T A C T G A A G C T | EWS:FLI1-fusion(ETS)/SK\_N\_MC-EWS:FLI1-ChIP-Seq(SRA014231)/Homer | 1e-3 | -8.661e+00 | 0.0014 | 11.0 | 5.88% | 13.9 | 1.24% | motif file (matrix) | svg |
| 127 | G A T C G A T C G A T C C G T A G T A C A G T C G C A T C G T A G T A C G A T C | MYB58(MYB)/colamp-MYB58-DAP-Seq(GSE60143)/Homer | 1e-3 | -8.627e+00 | 0.0014 | 61.0 | 32.62% | 227.2 | 20.23% | motif file (matrix) | svg |
| 128 | A T G C A G T C A G C T A G C T A C G T A T C G C G T A C G A T T A G C G A C T | LEF1(HMG)/H1-LEF1-ChIP-Seq(GSE64758)/Homer | 1e-3 | -8.608e+00 | 0.0014 | 55.0 | 29.41% | 197.3 | 17.56% | motif file (matrix) | svg |
| 129 | A G C T A G T C C G T A A G T C C T A G A C G T A C T G T C A G C G A T C A T G | PIF5ox(bHLH)/Arabidopsis-PIF5ox-ChIP-Seq(GSE35062)/Homer | 1e-3 | -8.587e+00 | 0.0015 | 154.0 | 82.35% | 784.2 | 69.80% | motif file (matrix) | svg |
| 130 | C A T G G T A C G A T C A C T G A C G T A G C T C G T A C G T A | AT3G10030(Trihelix)/colamp-AT3G10030-DAP-Seq(GSE60143)/Homer | 1e-3 | -8.578e+00 | 0.0015 | 129.0 | 68.98% | 617.0 | 54.92% | motif file (matrix) | svg |
| 131 | T C G A A G T C C G T A A T C G T A G C A C G T A C T G A G C T A C G T A G T C | Ptf1a(bHLH)/Panc1-Ptf1a-ChIP-Seq(GSE47459)/Homer | 1e-3 | -8.520e+00 | 0.0015 | 169.0 | 90.37% | 896.8 | 79.82% | motif file (matrix) | svg |
| 132 | C T A G A C T G A C G T C G T A A C T G C A T G G C A T T C A G | MYB92(MYB)/colamp-MYB92-DAP-Seq(GSE60143)/Homer | 1e-3 | -8.517e+00 | 0.0015 | 61.0 | 32.62% | 228.9 | 20.38% | motif file (matrix) | svg |
| 133 | A G T C A C T G A C G T C A T G C T G A G C T A C G A T C G A T G A C T G A C T G T C A G T A C A C T G A C T G G A T C | ANAC042(NAC)/col-ANAC042-DAP-Seq(GSE60143)/Homer | 1e-3 | -8.503e+00 | 0.0015 | 53.0 | 28.34% | 188.5 | 16.77% | motif file (matrix) | svg |
| 134 | G A T C G C T A G T A C A G T C G C T A T G C A G T A C G A T C C G T A G A C T | MYB83(MYB)/colamp-MYB83-DAP-Seq(GSE60143)/Homer | 1e-3 | -8.471e+00 | 0.0016 | 65.0 | 34.76% | 249.3 | 22.19% | motif file (matrix) | svg |
| 135 | C G A T C T G A C G T A C A G T A G T C G A T C G A T C A C T G | Pitx1(Homeobox)/Chicken-Pitx1-ChIP-Seq(GSE38910)/Homer | 1e-3 | -8.455e+00 | 0.0016 | 176.0 | 94.12% | 954.5 | 84.95% | motif file (matrix) | svg |
| 136 | C T G A A T G C G C T A C G A T A T G C C G T A C G T A C G T A C T A G T A C G | Tcf3(HMG)/mES-Tcf3-ChIP-Seq(GSE11724)/Homer | 1e-3 | -8.393e+00 | 0.0017 | 52.0 | 27.81% | 184.3 | 16.41% | motif file (matrix) | svg |
| 137 | A C T G T G A C G T A C C G T A A G T C T A C G A C G T A C T G G T C A A G T C | NPAS2(bHLH)/Liver-NPAS2-ChIP-Seq(GSE39860)/Homer | 1e-3 | -8.389e+00 | 0.0017 | 162.0 | 86.63% | 844.2 | 75.14% | motif file (matrix) | svg |
| 138 | C T G A C T G A C A T G A T C G A G C T A T C G G A C T C A T G C T G A G T C A | Tbr1(T-box)/Cortex-Tbr1-ChIP-Seq(GSE71384)/Homer | 1e-3 | -8.295e+00 | 0.0018 | 128.0 | 68.45% | 614.2 | 54.67% | motif file (matrix) | svg |
| 139 | C G A T C G A T G C A T G C A T G T C A A G T C A G C T A C G T A C G T C G A T G A C T A C G T | OBP4(C2C2dof)/col-OBP4-DAP-Seq(GSE60143)/Homer | 1e-3 | -8.286e+00 | 0.0018 | 78.0 | 41.71% | 320.6 | 28.54% | motif file (matrix) | svg |
| 140 | T A C G A T G C G C T A A C T G C G T A A C G T C G T A C T G A T A C G T C G A | Gata4(Zf)/Heart-Gata4-ChIP-Seq(GSE35151)/Homer | 1e-3 | -8.242e+00 | 0.0019 | 163.0 | 87.17% | 853.6 | 75.98% | motif file (matrix) | svg |
| 141 | A G C T A G T C A T G C A G C T A C G T C G T A A C G T A G T C C G A T A T G C | Gata2(Zf)/K562-GATA2-ChIP-Seq(GSE18829)/Homer | 1e-3 | -8.205e+00 | 0.0019 | 95.0 | 50.80% | 416.1 | 37.04% | motif file (matrix) | svg |
| 142 | C G A T T G C A G T A C C G T A A G T C C T A G G A C T C A T G | NPAS(bHLH)/Liver-NPAS-ChIP-Seq(GSE39860)/Homer | 1e-3 | -8.169e+00 | 0.0020 | 161.0 | 86.10% | 839.7 | 74.74% | motif file (matrix) | svg |
| 143 | C T A G A C T G A C G T C G T A A C T G A C T G A G C T C T A G T C A G C T A G | MYB93(MYB)/colamp-MYB93-DAP-Seq(GSE60143)/Homer | 1e-3 | -8.136e+00 | 0.0021 | 64.0 | 34.22% | 247.9 | 22.06% | motif file (matrix) | svg |
| 144 | G A C T C G A T T C A G G A T C G A C T A G C T A G C T A G T C G A T C C G T A C T A G C T A G T C G A T C G A C T G A | Bcl6(Zf)/Liver-Bcl6-ChIP-Seq(GSE31578)/Homer | 1e-3 | -7.995e+00 | 0.0024 | 37.0 | 19.79% | 116.2 | 10.34% | motif file (matrix) | svg |
| 145 | C T G A A T G C G C T A G C A T A T G C C G T A T C G A C T G A C T A G T C A G T A C G G T C A | Tcf4(HMG)/Hct116-Tcf4-ChIP-Seq(SRA012054)/Homer | 1e-3 | -7.923e+00 | 0.0025 | 52.0 | 27.81% | 188.2 | 16.75% | motif file (matrix) | svg |
| 146 | C A G T G C T A G C A T T A C G C T G A C A G T T A G C C T G A | GATA15(C2C2gata)/col-GATA15-DAP-Seq(GSE60143)/Homer | 1e-3 | -7.781e+00 | 0.0029 | 163.0 | 87.17% | 858.3 | 76.39% | motif file (matrix) | svg |
| 147 | G A T C G C A T A G T C A G C T G A T C A G C T G A T C G A C T G A T C G A C T A G T C A C G T G A T C A G C T G A T C | GAGA-repeat/SacCer-Promoters/Homer | 1e-3 | -7.717e+00 | 0.0030 | 170.0 | 90.91% | 912.0 | 81.18% | motif file (matrix) | svg |
| 148 | G C A T C G T A G C T A G A C T C G A T G A C T A G T C C A G T A G T C A G T C A C T G C T A G G T A C C T A G C T G A | AT5G05550(Trihelix)/col-AT5G05550-DAP-Seq(GSE60143)/Homer | 1e-3 | -7.679e+00 | 0.0031 | 30.0 | 16.04% | 87.4 | 7.78% | motif file (matrix) | svg |
| 149 | A G C T A T G C G A C T G C A T C G T A A G C T G T A C C G A T A T C G A G T C | Gata6(Zf)/HUG1N-GATA6-ChIP-Seq(GSE51936)/Homer | 1e-3 | -7.606e+00 | 0.0034 | 95.0 | 50.80% | 423.8 | 37.72% | motif file (matrix) | svg |
| 150 | G C A T A C G T A T G C C T G A C T A G G A C T G A T C A C T G | Initiator/Drosophila-Promoters/Homer | 1e-3 | -7.454e+00 | 0.0039 | 146.0 | 78.07% | 742.4 | 66.08% | motif file (matrix) | svg |
| 151 | G T A C G T C A G T A C G T C A G T A C G T C A G T A C G T C A G T A C G T C A | SeqBias: CA-repeat | 1e-3 | -7.450e+00 | 0.0039 | 175.0 | 93.58% | 955.4 | 85.03% | motif file (matrix) | svg |
| 152 | G C T A C G T A A C T G C G T A C G A T A C G T A G T C A G C T | At3g12730(G2like)/colamp-At3g12730-DAP-Seq(GSE60143)/Homer | 1e-3 | -7.436e+00 | 0.0039 | 117.0 | 62.57% | 556.0 | 49.49% | motif file (matrix) | svg |
| 153 | A T G C G A T C G A C T A G C T C G A T C G A T G T C A C G A T T C G A A T C G T A G C T A G C | TATA-Box(TBP)/Promoter/Homer | 1e-3 | -7.413e+00 | 0.0040 | 79.0 | 42.25% | 335.5 | 29.86% | motif file (matrix) | svg |
| 154 | T C G A T G A C A G T C C G T A A G T C C T A G A C G T A C T G A C T G A G C T A G T C G C A T | Max(bHLH)/K562-Max-ChIP-Seq(GSE31477)/Homer | 1e-3 | -7.310e+00 | 0.0044 | 150.0 | 80.21% | 771.6 | 68.67% | motif file (matrix) | svg |
| 155 | G T A C A G C T T C G A G T A C A G T C C A G T C G T A G T C A G A T C G C A T | MYB62(MYB)/colamp-MYB62-DAP-Seq(GSE60143)/Homer | 1e-3 | -7.309e+00 | 0.0044 | 63.0 | 33.69% | 250.1 | 22.26% | motif file (matrix) | svg |
| 156 | G T A C C A T G A C T G C G T A C G T A G C A T G C A T G A C T T G A C G T A C A C T G T A C G | LBD18(LOBAS2)/colamp-LBD18-DAP-Seq(GSE60143)/Homer | 1e-3 | -7.288e+00 | 0.0044 | 153.0 | 81.82% | 792.4 | 70.53% | motif file (matrix) | svg |
| 157 | A T G C G T A C C G T A A G C T G C A T T A C G A G C T A G C T A G T C A G C T | Sox6(HMG)/Myotubes-Sox6-ChIP-Seq(GSE32627)/Homer | 1e-3 | -7.188e+00 | 0.0048 | 161.0 | 86.10% | 850.3 | 75.68% | motif file (matrix) | svg |
| 158 | G C A T G C A T G C A T C G T A C G T A A C G T A C T G A C T G A C T G A G T C A G T C C G A T C G T A C G A T C G A T | PCF/Arabidopsis-Promoters/Homer | 1e-3 | -7.178e+00 | 0.0049 | 17.0 | 9.09% | 37.0 | 3.30% | motif file (matrix) | svg |
| 159 | C G T A C G A T C G T A T C G A T C G A A C G T C G T A A C G T A G T C G C A T | LHY(Myb)/Seedling-LHY-ChIP-Seq(GSE52175)/Homer | 1e-3 | -7.134e+00 | 0.0050 | 35.0 | 18.72% | 113.9 | 10.14% | motif file (matrix) | svg |
| 160 | T A C G T G C A A G T C C G T A A C G T T G A C A C G T A C T G A C T G G C A T | TCF4(bHLH)/SHSY5Y-TCF4-ChIP-Seq(GSE96915)/Homer | 1e-3 | -7.038e+00 | 0.0055 | 155.0 | 82.89% | 809.8 | 72.08% | motif file (matrix) | svg |
| 161 | C G T A A T C G A T G C T A G C C T G A A G T C T C A G G C A T T C A G T C A G | PIF4(bHLH)/Seedling-PIF4-ChIP-Seq(GSE35315)/Homer | 1e-3 | -7.015e+00 | 0.0056 | 148.0 | 79.14% | 761.9 | 67.81% | motif file (matrix) | svg |
| 162 | G A C T C T A G A T G C A G T C G T C A T A C G A T G C A T C G | HIC1(Zf)/Treg-ZBTB29-ChIP-Seq(GSE99889)/Homer | 1e-3 | -6.997e+00 | 0.0057 | 71.0 | 37.97% | 296.6 | 26.40% | motif file (matrix) | svg |
| 163 | G A T C G T A C C G A T A C T G A C T G C G T A C G T A A C G T A C T G G A T C | TEAD(TEA)/Fibroblast-PU.1-ChIP-Seq(Unpublished)/Homer | 1e-3 | -6.977e+00 | 0.0058 | 140.0 | 74.87% | 708.7 | 63.08% | motif file (matrix) | svg |
| 164 | T C G A T G A C G C A T A G C T C A G T G A T C G C T A G A T C G A C T A C G T G C A T A G T C | PRDM1(Zf)/Hela-PRDM1-ChIP-Seq(GSE31477)/Homer | 1e-2 | -6.863e+00 | 0.0064 | 22.0 | 11.76% | 58.9 | 5.24% | motif file (matrix) | svg |
| 165 | G A C T A G T C G A T C C G T A G T A C A G T C G C A T C G T A G T C A G A T C | MYB67(MYB)/col-MYB67-DAP-Seq(GSE60143)/Homer | 1e-2 | -6.861e+00 | 0.0064 | 64.0 | 34.22% | 260.6 | 23.19% | motif file (matrix) | svg |
| 166 | T C A G A C G T T C G A T A G C A G T C C G T A A C T G G T A C A C G T A C T G A T C G A G T C | Atoh1(bHLH)/Cerebellum-Atoh1-ChIP-Seq(GSE22111)/Homer | 1e-2 | -6.841e+00 | 0.0065 | 149.0 | 79.68% | 770.5 | 68.57% | motif file (matrix) | svg |
| 167 | C G A T C T A G G A T C G C T A A G C T C T A G G A T C C G T A | RBFox2(?)/Heart-RBFox2-CLIP-Seq(GSE57926)/Homer | 1e-2 | -6.828e+00 | 0.0065 | 160.0 | 85.56% | 847.4 | 75.43% | motif file (matrix) | svg |
| 168 | T G C A G T A C C G T A A T C G A C T G A C G T C T A G C G A T T C G A A G T C | ZEB1(Zf)/PDAC-ZEB1-ChIP-Seq(GSE64557)/Homer | 1e-2 | -6.792e+00 | 0.0067 | 155.0 | 82.89% | 812.0 | 72.27% | motif file (matrix) | svg |
| 169 | C T A G T A C G A G T C C G T A A G T C A C G T A G T C T C G A C G T A T A C G | Nkx2.1(Homeobox)/LungAC-Nkx2.1-ChIP-Seq(GSE43252)/Homer | 1e-2 | -6.779e+00 | 0.0068 | 167.0 | 89.30% | 899.1 | 80.02% | motif file (matrix) | svg |
| 170 | C A G T G A C T G C A T T C G A A G T C A C G T A C G T A C G T C G A T G A C T | OBP3(C2C2dof)/col-OBP3-DAP-Seq(GSE60143)/Homer | 1e-2 | -6.716e+00 | 0.0072 | 141.0 | 75.40% | 718.9 | 63.99% | motif file (matrix) | svg |
| 171 | G A C T C T A G C T A G G T A C A G T C G A T C G A C T G A C T T A G C T C A G | NLP7(RWPRK)/col-NLP7-DAP-Seq(GSE60143)/Homer | 1e-2 | -6.715e+00 | 0.0072 | 157.0 | 83.96% | 827.3 | 73.63% | motif file (matrix) | svg |
| 172 | G C A T A C G T A C T G A C G T A G T C A C T G A T C G G T C A C G A T C G T A | ARF2(ARF)/col-ARF2-DAP-Seq(GSE60143)/Homer | 1e-2 | -6.650e+00 | 0.0076 | 168.0 | 89.84% | 908.9 | 80.90% | motif file (matrix) | svg |
| 173 | C A T G A C G T A G T C G A T C G A T C G A T C G C T A C T A G C T A G C T A G T C A G T C G A | EBF1(EBF)/Near-E2A-ChIP-Seq(GSE21512)/Homer | 1e-2 | -6.622e+00 | 0.0077 | 148.0 | 79.14% | 766.0 | 68.18% | motif file (matrix) | svg |
| 174 | T C A G C G T A C G A T C G A T A G T C T A G C T A C G C A G T G A C T C T G A | MYB105(MYB)/colamp-MYB105-DAP-Seq(GSE60143)/Homer | 1e-2 | -6.609e+00 | 0.0078 | 123.0 | 65.78% | 604.1 | 53.77% | motif file (matrix) | svg |
| 175 | A G T C G A C T A G C T C G A T A T C G G C T A C G A T A T C G C G A T A C T G T A C G A C G T | Tcf7(HMG)/GM12878-TCF7-ChIP-Seq(Encode)/Homer | 1e-2 | -6.605e+00 | 0.0078 | 57.0 | 30.48% | 226.9 | 20.19% | motif file (matrix) | svg |
| 176 | C A T G A C T G C A T G A C T G T A C G A T G C A G T C G T A C G T A C T G A C G A T C G A C T | TCP1(TCP)/col-TCP1-DAP-Seq(GSE60143)/Homer | 1e-2 | -6.574e+00 | 0.0080 | 60.0 | 32.09% | 242.1 | 21.54% | motif file (matrix) | svg |
| 177 | C T G A T G A C T G A C C G T A A C G T T G A C A G C T C T A G A C G T G A C T | Olig2(bHLH)/Neuron-Olig2-ChIP-Seq(GSE30882)/Homer | 1e-2 | -6.551e+00 | 0.0081 | 155.0 | 82.89% | 815.9 | 72.62% | motif file (matrix) | svg |
| 178 | C G T A C T A G C G A T G A T C C T G A A C T G C A G T G C A T C G T A A T C G | MYB33(MYB)/col-MYB33-DAP-Seq(GSE60143)/Homer | 1e-2 | -6.521e+00 | 0.0083 | 140.0 | 74.87% | 714.7 | 63.61% | motif file (matrix) | svg |
| 179 | A G T C A C G T A C T G A G C T A C G T A C G T G T C A A G T C | Foxo1(Forkhead)/RAW-Foxo1-ChIP-Seq(Fan\_et\_al.)/Homer | 1e-2 | -6.427e+00 | 0.0091 | 161.0 | 86.10% | 859.2 | 76.47% | motif file (matrix) | svg |
| 180 | T A G C T A G C G C A T C A T G A C T G G C T A C G T A A C G T A C T G G A T C | TEAD4(TEA)/Tropoblast-Tead4-ChIP-Seq(GSE37350)/Homer | 1e-2 | -6.405e+00 | 0.0092 | 139.0 | 74.33% | 709.7 | 63.17% | motif file (matrix) | svg |
| 181 | T C A G T G A C G T A C C G T A A C G T T G A C A C G T T C A G A G C T G A C T | NeuroD1(bHLH)/Islet-NeuroD1-ChIP-Seq(GSE30298)/Homer | 1e-2 | -6.298e+00 | 0.0102 | 149.0 | 79.68% | 777.5 | 69.20% | motif file (matrix) | svg |
| 182 | G A C T G C A T G C A T A G T C A G C T T C G A T A C G G C T A C G T A A C T G G T A C G C A T C G A T A G T C G A C T | HSF3(HSF)/colamp-HSF3-DAP-Seq(GSE60143)/Homer | 1e-2 | -6.295e+00 | 0.0102 | 10.0 | 5.35% | 16.0 | 1.43% | motif file (matrix) | svg |
| 183 | G A C T A C G T C G A T A G C T A G T C C G T A A C T G A C T G C G A T C T A G | NGA4(ABI3VP1)/col-NGA4-DAP-Seq(GSE60143)/Homer | 1e-2 | -6.283e+00 | 0.0103 | 150.0 | 80.21% | 784.8 | 69.85% | motif file (matrix) | svg |
| 184 | C A G T C A T G T G C A G T A C C G T A T C A G G T A C G A C T T C A G C T G A | bZIP18(bZIP)/colamp-bZIP18-DAP-Seq(GSE60143)/Homer | 1e-2 | -6.268e+00 | 0.0104 | 165.0 | 88.24% | 890.8 | 79.28% | motif file (matrix) | svg |
| 185 | A G C T C T A G T G A C C G T A A C G T C G A T A G T C A G T C C T G A C A T G | TEAD3(TEA)/HepG2-TEAD3-ChIP-Seq(Encode)/Homer | 1e-2 | -6.267e+00 | 0.0104 | 141.0 | 75.40% | 724.9 | 64.52% | motif file (matrix) | svg |
| 186 | C T A G A G T C T A C G T A C G T G A C C G T A A C T G T A G C G C A T C A T G A T G C A G C T | Ascl1(bHLH)/NeuralTubes-Ascl1-ChIP-Seq(GSE55840)/Homer | 1e-2 | -6.257e+00 | 0.0104 | 152.0 | 81.28% | 798.6 | 71.08% | motif file (matrix) | svg |
| 187 | T C A G A G C T A T G C C G T A A G C T T C A G C A G T A C T G C T G A A G T C | MITF(bHLH)/MastCells-MITF-ChIP-Seq(GSE48085)/Homer | 1e-2 | -6.248e+00 | 0.0104 | 153.0 | 81.82% | 805.4 | 71.68% | motif file (matrix) | svg |
| 188 | G C A T G C T A C G T A A G C T G C T A T G C A A G T C A C G T A C G T A C G T G C A T G C A T | At4g38000(C2C2dof)/col-At4g38000-DAP-Seq(GSE60143)/Homer | 1e-2 | -6.192e+00 | 0.0110 | 46.0 | 24.60% | 174.7 | 15.55% | motif file (matrix) | svg |
| 189 | A G T C G T A C A G C T C T A G A G T C C G A T A C T G C G T A A C T G G T C A | Zic(Zf)/Cerebellum-ZIC1.2-ChIP-Seq(GSE60731)/Homer | 1e-2 | -6.063e+00 | 0.0124 | 30.0 | 16.04% | 98.4 | 8.76% | motif file (matrix) | svg |
| 190 | G C T A C G T A A C T G C G T A C G A T A C G T A G T C A G C T | At1g25550(G2like)/colamp-At1g25550-DAP-Seq(GSE60143)/Homer | 1e-2 | -6.045e+00 | 0.0125 | 109.0 | 58.29% | 526.6 | 46.87% | motif file (matrix) | svg |
| 191 | C T G A C G A T C T A G T C A G G A T C C T G A T C A G G A T C C T G A A C T G A G T C G C T A A C G T A G T C G C A T | PRDM9(Zf)/Testis-DMC1-ChIP-Seq(GSE35498)/Homer | 1e-2 | -5.979e+00 | 0.0133 | 7.0 | 3.74% | 8.6 | 0.76% | motif file (matrix) | svg |
| 192 | G A C T T C A G C T A G A G T C A G T C G T A C A G T C C T G A A G T C A G T C A G T C G A C T A G T C A C T G A T G C | KLF3(Zf)/MEF-Klf3-ChIP-Seq(GSE44748)/Homer | 1e-2 | -5.963e+00 | 0.0135 | 10.0 | 5.35% | 17.1 | 1.52% | motif file (matrix) | svg |
| 193 | T G C A A G C T A C G T C T A G G A T C C T A G G A T C G T C A C T G A A G T C | CEBP(bZIP)/ThioMac-CEBPb-ChIP-Seq(GSE21512)/Homer | 1e-2 | -5.937e+00 | 0.0138 | 145.0 | 77.54% | 755.6 | 67.25% | motif file (matrix) | svg |
| 194 | T G A C A G T C C T G A T G A C C G T A A C G T A C G T A G T C A G T C C G T A | TEAD1(TEAD)/HepG2-TEAD1-ChIP-Seq(Encode)/Homer | 1e-2 | -5.936e+00 | 0.0138 | 140.0 | 74.87% | 722.5 | 64.31% | motif file (matrix) | svg |
| 195 | T G A C A G T C C G T A A C T G G T A C A C G T A C T G A C G T G A C T G A T C | Twist2(bHLH)/Myoblast-Twist2.Ty1-ChIP-Seq(GSE127998)/Homer | 1e-2 | -5.859e+00 | 0.0147 | 154.0 | 82.35% | 817.2 | 72.73% | motif file (matrix) | svg |
| 196 | C T G A T A C G A C G T C T A G C G T A T C G A C T G A C G A T | At5g04390(C2H2)/col200-At5g04390-DAP-Seq(GSE60143)/Homer | 1e-2 | -5.846e+00 | 0.0148 | 143.0 | 76.47% | 743.9 | 66.21% | motif file (matrix) | svg |
| 197 | A G T C G A T C G A T C C G T A G T C A A G T C A G C T C T G A G A C T G A C T | ATY13(MYB)/col-ATY13-DAP-Seq(GSE60143)/Homer | 1e-2 | -5.810e+00 | 0.0153 | 163.0 | 87.17% | 881.1 | 78.42% | motif file (matrix) | svg |
| 198 | C G T A C G T A G C A T A C T G C G T A A G C T C T G A C G T A T A C G C T G A | ELT-3(Gata)/cElegans-L1-ELT3-ChIP-Seq(modEncode)/Homer | 1e-2 | -5.806e+00 | 0.0153 | 54.0 | 28.88% | 219.5 | 19.54% | motif file (matrix) | svg |
| 199 | T A G C G C A T A G T C G A T C A T G C G A C T C T A G A C T G A C T G C T G A A C T G C T A G A G T C T G A C C G A T | GLIS3(Zf)/Thyroid-Glis3.GFP-ChIP-Seq(GSE103297)/Homer | 1e-2 | -5.793e+00 | 0.0154 | 153.0 | 81.82% | 811.3 | 72.21% | motif file (matrix) | svg |
| 200 | C G A T G C T A G C T A G C A T G C T A C G T A A G T C A C G T A C G T A C G T C G A T A G C T | At5g62940(C2C2dof)/col-At5g62940-DAP-Seq(GSE60143)/Homer | 1e-2 | -5.770e+00 | 0.0157 | 165.0 | 88.24% | 896.4 | 79.78% | motif file (matrix) | svg |
| 201 | A T C G A G T C A G T C G A C T A T G C C T G A C T A G A C T G T A C G G T A C C T G A C G A T | AP-2gamma(AP2)/MCF7-TFAP2C-ChIP-Seq(GSE21234)/Homer | 1e-2 | -5.744e+00 | 0.0160 | 126.0 | 67.38% | 635.2 | 56.53% | motif file (matrix) | svg |
| 202 | C G T A C G T A C G T A C G A T C A T G T A C G G A C T A T G C C G T A A G T C C T A G A C G T A C T G C A T G G T A C | bZIP48(bZIP)/colamp-bZIP48-DAP-Seq(GSE60143)/Homer | 1e-2 | -5.743e+00 | 0.0160 | 91.0 | 48.66% | 424.3 | 37.76% | motif file (matrix) | svg |
| 203 | C G T A G C T A C G A T C A T G G T A C A G T C C G T A A G T C A C T G A C G T T A G C C G T A A G T C G T A C C G T A | GBF6(bZIP)/colamp-GBF6-DAP-Seq(GSE60143)/Homer | 1e-2 | -5.743e+00 | 0.0160 | 91.0 | 48.66% | 424.5 | 37.78% | motif file (matrix) | svg |
| 204 | T C G A T G A C G T A C C G T A C A G T T G A C A C G T A C T G A G C T A G C T | NeuroG2(bHLH)/Fibroblast-NeuroG2-ChIP-Seq(GSE75910)/Homer | 1e-2 | -5.709e+00 | 0.0164 | 154.0 | 82.35% | 819.2 | 72.91% | motif file (matrix) | svg |
| 205 | G A C T C A G T G A T C G A T C A C G T G A T C C T G A T A C G C G T A G T C A | STAT6(Stat)/Macrophage-Stat6-ChIP-Seq(GSE38377)/Homer | 1e-2 | -5.676e+00 | 0.0168 | 6.0 | 3.21% | 6.3 | 0.56% | motif file (matrix) | svg |
| 206 | C G T A C G T A C G T A C G T A C G T A A C T G A G C T C T A G G T A C G C T A | AT1G69570(C2C2dof)/col-AT1G69570-DAP-Seq(GSE60143)/Homer | 1e-2 | -5.651e+00 | 0.0172 | 55.0 | 29.41% | 227.0 | 20.20% | motif file (matrix) | svg |
| 207 | G C T A G A C T A G T C T C G A T C A G T C G A A C G T A G T C G A C T T C A G | GATA14(C2C2gata)/col-GATA14-DAP-Seq(GSE60143)/Homer | 1e-2 | -5.634e+00 | 0.0174 | 103.0 | 55.08% | 496.9 | 44.23% | motif file (matrix) | svg |
| 208 | A C G T T A G C T G C A G A T C T C A G A C G T A C T G T G C A G A T C G A T C | Cbf1(bHLH)/Yeast-Cbf1-ChIP-Seq(GSE29506)/Homer | 1e-2 | -5.632e+00 | 0.0174 | 94.0 | 50.27% | 443.2 | 39.45% | motif file (matrix) | svg |
| 209 | G A C T A G T C C G T A C G T A A G T C A G C T A C T G G A C T G T A C A T G C | MYB77(MYB)/col-MYB77-DAP-Seq(GSE60143)/Homer | 1e-2 | -5.537e+00 | 0.0190 | 144.0 | 77.01% | 754.2 | 67.13% | motif file (matrix) | svg |
| 210 | T A C G C T A G T A C G A G T C C G T A A G T C A G T C A C G T A C T G A G T C G A T C T A G C | Slug(Zf)/Mesoderm-Snai2-ChIP-Seq(GSE61475)/Homer | 1e-2 | -5.513e+00 | 0.0193 | 113.0 | 60.43% | 558.4 | 49.70% | motif file (matrix) | svg |
| 211 | T A C G T C G A C G T A C G T A C G T A C T G A A C T G A C G T C G T A T C G A | AT2G28810(C2C2dof)/colamp-AT2G28810-DAP-Seq(GSE60143)/Homer | 1e-2 | -5.483e+00 | 0.0198 | 137.0 | 73.26% | 709.2 | 63.12% | motif file (matrix) | svg |
| 212 | C T A G G C A T G A T C C G T A A G T C T C A G G A C T C T A G | CLOCK(bHLH)/Liver-Clock-ChIP-Seq(GSE39860)/Homer | 1e-2 | -5.431e+00 | 0.0208 | 103.0 | 55.08% | 499.7 | 44.47% | motif file (matrix) | svg |
| 213 | C T G A C T A G G C A T A T G C C G T A A G T C A C T G A C G T T A C G C T G A | HY5(bZIP)/colamp-HY5-DAP-Seq(GSE60143)/Homer | 1e-2 | -5.397e+00 | 0.0214 | 97.0 | 51.87% | 464.5 | 41.34% | motif file (matrix) | svg |
| 214 | G C A T T C A G C T G A A T C G A C T G C G A T G A T C C T G A | THRb(NR)/Liver-NR1A2-ChIP-Seq(GSE52613)/Homer | 1e-2 | -5.396e+00 | 0.0214 | 161.0 | 86.10% | 872.1 | 77.62% | motif file (matrix) | svg |
| 215 | T G C A C T G A A T G C G T C A A C G T A T G C A C G T A C T G A C T G T G C A | ZBTB18(Zf)/HEK293-ZBTB18.GFP-ChIP-Seq(GSE58341)/Homer | 1e-2 | -5.392e+00 | 0.0214 | 12.0 | 6.42% | 26.3 | 2.34% | motif file (matrix) | svg |
| 216 | T G C A C G T A G T C A A G C T A G T C G C T A T A G C C G A T C T A G G A T C | Gfi1b(Zf)/HPC7-Gfi1b-ChIP-Seq(GSE22178)/Homer | 1e-2 | -5.355e+00 | 0.0220 | 135.0 | 72.19% | 698.9 | 62.21% | motif file (matrix) | svg |
| 217 | A G T C C G T A A C G T A G T C A C G T A C T G | Tal1 | 1e-2 | -5.314e+00 | 0.0228 | 151.0 | 80.75% | 804.4 | 71.59% | motif file (matrix) | svg |
| 218 | A G T C C T G A A G T C C G A T C A G T G A T C A T G C A C T G A T C G G A C T | Fli1(ETS)/CD8-FLI-ChIP-Seq(GSE20898)/Homer | 1e-2 | -5.301e+00 | 0.0230 | 63.0 | 33.69% | 273.4 | 24.34% | motif file (matrix) | svg |
| 219 | C G A T C T A G C T G A A T G C C T G A T C G A C G T A C T G A T C G A T A G C A G T C C G T A A C T G T C G A A T G C | Hand2(bHLH)/Mesoderm-Hand2-ChIP-Seq(GSE61475)/Homer | 1e-2 | -5.292e+00 | 0.0231 | 16.0 | 8.56% | 42.1 | 3.75% | motif file (matrix) | svg |
| 220 | T G A C G C T A T G A C C G T A T C A G G A T C C G T A C A T G C A T G C T A G C T A G C T A G | Unknown-ESC-element(?)/mES-Nanog-ChIP-Seq(GSE11724)/Homer | 1e-2 | -5.285e+00 | 0.0232 | 25.0 | 13.37% | 81.1 | 7.22% | motif file (matrix) | svg |
| 221 | G C T A C G A T C A T G A T G C A G T C A G T C G A C T T A C G T C G A C T A G A C T G T A G C | AP-2alpha(AP2)/Hela-AP2alpha-ChIP-Seq(GSE31477)/Homer | 1e-2 | -5.277e+00 | 0.0232 | 122.0 | 65.24% | 617.5 | 54.96% | motif file (matrix) | svg |
| 222 | G A C T G A T C G A T C G C T A G T A C A G T C G C T A C T G A G T A C G A T C G C T A G A C T | MYB13(MYB)/col-MYB13-DAP-Seq(GSE60143)/Homer | 1e-2 | -5.254e+00 | 0.0237 | 48.0 | 25.67% | 194.0 | 17.27% | motif file (matrix) | svg |
| 223 | C T A G T A C G G A T C G T A C G C T A A G C T A G C T G T C A T C G A T A G C | Nanog(Homeobox)/mES-Nanog-ChIP-Seq(GSE11724)/Homer | 1e-2 | -5.245e+00 | 0.0238 | 161.0 | 86.10% | 874.6 | 77.85% | motif file (matrix) | svg |
| 224 | C T A G C T G A C T A G C T G A C T A G C T G A C T A G C T G A C T A G C T G A | SeqBias: GA-repeat | 1e-2 | -5.218e+00 | 0.0243 | 175.0 | 93.58% | 978.6 | 87.10% | motif file (matrix) | svg |
| 225 | T C G A C T G A C T G A A T G C G T A C G T C A A G C T A G C T C G T A T C A G | Barx1(Homeobox)/Stomach-Barx1.3xFlag-ChIP-Seq(GSE69483)/Homer | 1e-2 | -5.217e+00 | 0.0243 | 71.0 | 37.97% | 318.9 | 28.39% | motif file (matrix) | svg |
| 226 | G C A T A G C T A C G T A C G T A C T G A C G T G A T C A C G T A C G T A G C T C G A T G C A T A G T C G A C T C A G T | IDD5(C2H2)/colamp-IDD5-DAP-Seq(GSE60143)/Homer | 1e-2 | -5.195e+00 | 0.0247 | 134.0 | 71.66% | 694.6 | 61.82% | motif file (matrix) | svg |
| 227 | C G A T C T A G A C T G A G C T C T G A A C T G A C G T A C G T C T A G C T A G | MYB96(MYB)/colamp-MYB96-DAP-Seq(GSE60143)/Homer | 1e-2 | -5.183e+00 | 0.0249 | 49.0 | 26.20% | 200.1 | 17.81% | motif file (matrix) | svg |
| 228 | G T C A T A C G C G A T C T A G C T G A G C A T C G A T C A T G T C G A A G T C C G T A A G T C A C T G A C G T A C T G | bHLH34(bHLH)/colamp-bHLH34-DAP-Seq(GSE60143)/Homer | 1e-2 | -5.179e+00 | 0.0249 | 92.0 | 49.20% | 438.4 | 39.02% | motif file (matrix) | svg |
| 229 | C G A T A C G T A C G T A C G T C G T A A G C T C A G T C T A G A T C G A C T G | HOXB13(Homeobox)/ProstateTumor-HOXB13-ChIP-Seq(GSE56288)/Homer | 1e-2 | -5.148e+00 | 0.0255 | 141.0 | 75.40% | 740.5 | 65.90% | motif file (matrix) | svg |
| 230 | C G T A C G T A C G T A C G T A A G T C A G T C C T A G A T C G C G A T G C T A | AT1G76870(Trihelix)/col-AT1G76870-DAP-Seq(GSE60143)/Homer | 1e-2 | -5.144e+00 | 0.0255 | 116.0 | 62.03% | 582.4 | 51.84% | motif file (matrix) | svg |
| 231 | A G T C G A T C A G T C C G T A A T C G C A G T A G T C G T A C C T G A A C T G T C A G A G C T A G C T A G C T A G C T | PRDM15(Zf)/ESC-Prdm15-ChIP-Seq(GSE73694)/Homer | 1e-2 | -5.143e+00 | 0.0255 | 23.0 | 12.30% | 73.4 | 6.53% | motif file (matrix) | svg |
| 232 | C A T G G T A C C G T A A G T C C T A G A C G T A C T G G T A C A G T C A G C T | bHLHE40(bHLH)/HepG2-BHLHE40-ChIP-Seq(GSE31477)/Homer | 1e-2 | -5.132e+00 | 0.0256 | 97.0 | 51.87% | 468.8 | 41.72% | motif file (matrix) | svg |
| 233 | C A T G G A T C C T G A G T A C C T A G C T G A G C T A G C A T G A T C G A T C A G T C C T A G C G T A C A T G C T A G | AIL7(AP2EREBP)/colamp-AIL7-DAP-Seq(GSE60143)/Homer | 1e-2 | -5.127e+00 | 0.0256 | 7.0 | 3.74% | 11.0 | 0.98% | motif file (matrix) | svg |
| 234 | C G A T C G T A C G A T C G A T A G T C C T G A G A T C C T G A G A T C C T A G G C A T C T A G G A C T T C A G G C T A | At4g36780(BZR)/col-At4g36780-DAP-Seq(GSE60143)/Homer | 1e-2 | -5.075e+00 | 0.0269 | 96.0 | 51.34% | 463.9 | 41.29% | motif file (matrix) | svg |
| 235 | C T G A T C A G G T A C G C T A A C T G T G A C G C A T C A T G | SCL(bHLH)/HPC7-Scl-ChIP-Seq(GSE13511)/Homer | 1e-2 | -5.043e+00 | 0.0276 | 164.0 | 87.70% | 898.1 | 79.94% | motif file (matrix) | svg |
| 236 | G A T C C G T A G A C T C T A G G A T C C T G A G A C T C T G A G A C T C T A G G A T C C T G A G A C T C T G A G A C T | OCT:OCT(POU,Homeobox)/NPC-OCT6-ChIP-Seq(GSE43916)/Homer | 1e-2 | -5.003e+00 | 0.0286 | 125.0 | 66.84% | 640.1 | 56.97% | motif file (matrix) | svg |
| 237 | A C T G A G T C C G A T A C T G A G T C C G A T A C T G A G T C C G A T A C T G A G T C C G A T | SeqBias: GCW-triplet | 1e-2 | -4.993e+00 | 0.0288 | 187.0 | 100.00% | 1091.9 | 97.18% | motif file (matrix) | svg |
| 238 | A G C T G A C T A C T G A C G T G T C A A G T C A C G T C G A T | SPL9(SBP)/colamp-SPL9-DAP-Seq(GSE60143)/Homer | 1e-2 | -4.920e+00 | 0.0308 | 142.0 | 75.94% | 750.3 | 66.78% | motif file (matrix) | svg |
| 239 | C G T A A C G T A C G T A C G T A C G T A G T C A G T C C T G A A G C T A G C T | NFAT(RHD)/Jurkat-NFATC1-ChIP-Seq(Jolma\_et\_al.)/Homer | 1e-2 | -4.920e+00 | 0.0308 | 43.0 | 22.99% | 172.4 | 15.34% | motif file (matrix) | svg |
| 240 | C G T A C A G T C A T G G T A C A G T C C G T A A T G C A T C G A C G T T A C G C G T A G A T C G T A C C G T A C G T A | AREB3(bZIP)/col-AREB3-DAP-Seq(GSE60143)/Homer | 1e-2 | -4.864e+00 | 0.0322 | 91.0 | 48.66% | 437.0 | 38.90% | motif file (matrix) | svg |
| 241 | G C A T C A T G T C A G G A C T A T G C C G T A A G T C A C T G A C G T A C T G A C T G G T A C G C T A C G T A G C T A | bZIP53(bZIP)/colamp-bZIP53-DAP-Seq(GSE60143)/Homer | 1e-2 | -4.864e+00 | 0.0322 | 91.0 | 48.66% | 437.3 | 38.92% | motif file (matrix) | svg |
| 242 | G A C T C T A G C T A G A G T C T G C A A C T G A C G T A C G T C T A G T C A G | AMYB(HTH)/Testes-AMYB-ChIP-Seq(GSE44588)/Homer | 1e-2 | -4.851e+00 | 0.0325 | 150.0 | 80.21% | 804.1 | 71.57% | motif file (matrix) | svg |
| 243 | C G T A C T G A G A C T G T C A C G T A A C G T C G A T C G T A C G T A A C G T C A T G G C T A | HDG1(Homeobox)/col100-HDG1-DAP-Seq(GSE60143)/Homer | 1e-2 | -4.850e+00 | 0.0325 | 127.0 | 67.91% | 655.5 | 58.34% | motif file (matrix) | svg |
| 244 | T A G C G T A C A G T C G T A C C G A T A G T C A G T C A G T C A G T C A G T C C G T A G A T C | Zfp281(Zf)/ES-Zfp281-ChIP-Seq(GSE81042)/Homer | 1e-2 | -4.824e+00 | 0.0331 | 10.0 | 5.35% | 21.3 | 1.89% | motif file (matrix) | svg |
| 245 | C G A T C G T A G C A T G T A C G T A C A C T G A C G T C G T A A G T C C T G A C G T A C G T A | SPL15(SBP)/colamp-SPL15-DAP-Seq(GSE60143)/Homer | 1e-2 | -4.822e+00 | 0.0331 | 141.0 | 75.40% | 745.8 | 66.38% | motif file (matrix) | svg |
| 246 | C G A T G C A T A G T C T C A G G A T C C G T A G A T C C T A G G C A T C T A G A G C T T C G A C G T A G C T A G C A T | At1g78700(BZR)/col-At1g78700-DAP-Seq(GSE60143)/Homer | 1e-2 | -4.812e+00 | 0.0332 | 97.0 | 51.87% | 473.2 | 42.12% | motif file (matrix) | svg |
| 247 | T C A G G T A C C A T G C A G T C T G A C G T A C G A T G C A T G A C T G T C A G T A C A C T G A G T C G A C T C G A T | LBD23(LOBAS2)/colamp-LBD23-DAP-Seq(GSE60143)/Homer | 1e-2 | -4.771e+00 | 0.0345 | 45.0 | 24.06% | 184.2 | 16.39% | motif file (matrix) | svg |
| 248 | G A T C G C A T T C G A A G T C A C G T A C G T A C G T C G A T A C G T A T C G | AT1G47655(C2C2dof)/colamp-AT1G47655-DAP-Seq(GSE60143)/Homer | 1e-2 | -4.744e+00 | 0.0353 | 146.0 | 78.07% | 779.3 | 69.36% | motif file (matrix) | svg |
| 249 | T G C A C G T A A C T G T C A G C A G T C A T G T C A G G A T C T A C G A G T C T G C A A C T G A C T G T G A C G T C A | ZNF165(Zf)/WHIM12-ZNF165-ChIP-Seq(GSE65937)/Homer | 1e-2 | -4.740e+00 | 0.0353 | 48.0 | 25.67% | 200.1 | 17.81% | motif file (matrix) | svg |
| 250 | G C A T T C G A A C G T C G T A A C T G A C T G A G C T G C A T G A C T G C T A | SRS7(SRS)/colamp-SRS7-DAP-Seq(GSE60143)/Homer | 1e-2 | -4.715e+00 | 0.0361 | 154.0 | 82.35% | 833.5 | 74.18% | motif file (matrix) | svg |
| 251 | T C G A T A G C T G A C C T G A A G T C A C T G G A C T C A T G | c-Myc(bHLH)/LNCAP-cMyc-ChIP-Seq(Unpublished)/Homer | 1e-2 | -4.688e+00 | 0.0369 | 97.0 | 51.87% | 475.2 | 42.29% | motif file (matrix) | svg |
| 252 | C G T A C G A T C A T G A T C G G A C T A T G C C G T A A G T C C T A G C G A T C T A G C A T G G T A C C G T A C G A T | bZIP3(bZIP)/col-bZIP3-DAP-Seq(GSE60143)/Homer | 1e-2 | -4.647e+00 | 0.0383 | 94.0 | 50.27% | 458.8 | 40.83% | motif file (matrix) | svg |
| 253 | C T G A T A G C T G A C T C A G C T A G G T C A C G T A T C A G A G C T T C A G | ETV4(ETS)/HepG2-ETV4-ChIP-Seq(ENCODE)/Homer | 1e-2 | -4.635e+00 | 0.0386 | 61.0 | 32.62% | 271.6 | 24.18% | motif file (matrix) | svg |
| 254 | G T A C C A G T A C T G A C T G A C T G G A T C A C T G A C G T A C T G A C T G A G T C G A T C | KLF6(Zf)/PDAC-KLF6-ChIP-Seq(GSE64557)/Homer | 1e-2 | -4.614e+00 | 0.0393 | 152.0 | 81.28% | 821.6 | 73.12% | motif file (matrix) | svg |
| 255 | C T A G T C A G C T G A C T G A C T A G C T G A C A T G C A T G C T G A C T A G C T A G C G T A C T A G C G T A G T C A | TF3A(C2H2)/col-TF3A-DAP-Seq(GSE60143)/Homer | 1e-2 | -4.606e+00 | 0.0394 | 144.0 | 77.01% | 768.3 | 68.38% | motif file (matrix) | svg |
